# Frailty assessment tools for preoperative evaluation of older adults: scoping review

**DOI:** 10.1093/bjsopen/zrag129

**Published:** 2026-10-23

**Authors:** Tyler R Chesney, Camilla L Wong, Andrea C Tricco, Duminda N Wijeysundera, Marisa Louridas, Kesikan Jayaraj, Claire Sethuram, Amanda Mac, Shawn Khan, Victoria B Anthes, Madeline McDonald, Betty Yibrehu, Amanda Poxon, Anas Samman, Valerie Kim, Karim S Ladha, Teruko Kishibe, Samuel Dubé, Martine Puts, Shabbir M H Alibhai, Julian F Daza

**Affiliations:** Department of Surgery, Division of General Surgery, University of Toronto, Toronto, Ontario, Canada; Li Ka Shing Knowledge Institute, St. Michael's Hospital, Unity Health Toronto, Toronto, Ontario, Canada; Li Ka Shing Knowledge Institute, St. Michael's Hospital, Unity Health Toronto, Toronto, Ontario, Canada; Division of Geriatric Medicine, St. Michael’s Hospital, Unity Health Toronto, Toronto, Ontario, Canada; Institute of Health Policy, Management, and Evaluation, University of Toronto, Toronto, Ontario, Canada; Knowledge Translation Program, Li Ka Shing Knowledge Institute, St Michael's Hospital, Unity Health Toronto, Toronto, Ontario, Canada; Institute of Health Policy, Management, and Evaluation, University of Toronto, Toronto, Ontario, Canada; Department of Anesthesiology and Pain Medicine, St. Michael’s Hospital and University of Toronto, Toronto, Ontario, Canada; Department of Surgery, Division of General Surgery, University of Toronto, Toronto, Ontario, Canada; Department of Anesthesiology, McMaster University, Hamilton, Ontario, Canada; Department of Internal Medicine, University of Ottawa, Ottawa, Ontario, Canada; Temerty Faculty of Medicine, University of Toronto, Toronto, Ontario, Canada; Department of Emergency Medicine, Cambridge Memorial Hospital, Cambridge, Ontario, Canada; Temerty Faculty of Medicine, University of Toronto, Toronto, Ontario, Canada; Department of Surgery, McMaster University, Hamilton, Ontario, Canada; Department of Surgery, Division of General Surgery, University of Toronto, Toronto, Ontario, Canada; Temerty Faculty of Medicine, University of Toronto, Toronto, Ontario, Canada; Temerty Faculty of Medicine, University of Toronto, Toronto, Ontario, Canada; Temerty Faculty of Medicine, University of Toronto, Toronto, Ontario, Canada; Institute of Health Policy, Management, and Evaluation, University of Toronto, Toronto, Ontario, Canada; Department of Anesthesiology and Pain Medicine, St. Michael’s Hospital and University of Toronto, Toronto, Ontario, Canada; Li Ka Shing Knowledge Institute, St. Michael's Hospital, Unity Health Toronto, Toronto, Ontario, Canada; Division of Gynecologic Oncology, Université de Montréal, Montreal, Quebec, Canada; Lawrence S. Bloomberg Faculty of Nursing, University of Toronto, Toronto, Ontario, Canada; Institute of Health Policy, Management, and Evaluation, University of Toronto, Toronto, Ontario, Canada; Department of Medicine, University Health Network and University of Toronto, Toronto, Ontario, Canada; Department of Surgery, Division of General Surgery, University of Toronto, Toronto, Ontario, Canada; Institute of Health Policy, Management, and Evaluation, University of Toronto, Toronto, Ontario, Canada

**Keywords:** geriatrics, surgery

## Abstract

**Background:**

Optimal frailty assessment in surgery remains unclear due to insufficient tool performance evaluations. This scoping review summarizes evidence on frailty tools in the preoperative setting, focusing on key performance metrics.

**Methods:**

Following JBI and PRISMA-ScR recommendations, the MEDLINE, Embase, Cochrane Central Register of Controlled Trials, and Cumulative Index to Nursing and Allied Health Literature databases were systematically searched (2005–May 2026), for studies evaluating performance metrics of frailty tools in adults aged ≥ 65 years undergoing major intracavitary surgery. Two reviewers conducted screening and data charting. Data were analysed on settings, surgical types, and six performance metrics across measurement properties, clinical impact, and pragmatic factors.

**Results:**

From 12 272 records, 160 unique studies on 35 frailty tools were included. Some tools were extensively investigated (up to 41 studies and 11 287 participants), whereas 40% of tools had been investigated in only one study. Predictive validity was commonly studied, whereas reliability, clinical impact, and pragmatic factors were less explored. The Clinical Frailty Scale was extensively studied, covering four of six performance metrics across diverse settings and surgical types. The Edmonton Frail Scale was the only tool investigated across all six metrics, although reliance on performance and cognitive testing may hinder implementation. The FRAIL scale, Geriatric 8, and Vulnerable Elders Survey-13 are simple questionnaires, with the FRAIL scale being the most extensively investigated (five of six metrics).

**Conclusion:**

Several frailty assessment tools show promise based on investigation extent and features. This review aids clinical teams in identifying tools that meet specific local needs. Further research should focus on clinical impact and pragmatic factors, as well as tool-specific meta-analyses of performance metrics to enhance tool selection and implementation.

## Teaser

Frailty assessment is crucial in geriatric surgical care but is underutilized. This review catalogues 35 preoperative frailty tools, noting limited investigations for most. Some tools show robust evaluations across key metrics and diverse settings. This review aids tool selection and guides future research to enhance the level of investigation and improve frailty assessment implementation.

## Introduction

Unlike normal ageing, frailty is a state of increased vulnerability to stressors due to multisystem decline, commonly operationalized as a syndrome of accumulated deficits or as a phenotype, affecting 10–30% of older adults^1–3^. Major surgery is an important stressor, and frailty is associated with increased postoperative complications, mortality, delayed recovery, and functional decline^1,4,5^. Identifying frailty is important due to its link to poor outcomes, but frailty assessment remains underutilized despite guidelines^6,7^. Frailty assessment tools serve important diagnostic, prognostic, and clinical decision purposes, facilitating risk stratification, shared decision-making, and targeted multidisciplinary interventions such as prehabilitation and co-management^8–11^.

However, practical implementation of frailty assessment tools before surgery is hindered by the huge variety of tools with inconsistent levels of development and validation, leading to uncertainty among clinicians about which tool to use and in which setting^7,12–14^. Previous evidence synthesis efforts have compiled data across multiple tools, limiting the understanding of specific tools for clinical application, or focused only on prognostic performance ignoring other essential performance metrics^5,15–19^. A single review^20^ has synthesized evidence by individual frailty tool, but many surgical types were compiled and diagnostic or treatment effect properties were not examined. Another review^21^ specifically synthesized all key performance metrics of frailty assessment tools, but solely within the non-surgical context. The absence of a comprehensive assessment of the performance of individual tools has impeded guideline endorsement of specific tools, and thus limited integration of frailty assessment into routine preoperative care^7,22–32^.

To address this gap, a scoping review was conducted to summarize evidence on frailty assessment tools for preoperative use by surgeons and perioperative clinicians without formal training in geriatric medicine. A scoping review methodology was chosen because this design addresses broad questions, examining the extent and characteristics of published literature. The aims of this study were to: catalogue preoperative frailty assessment tools; describe the extent of data for each tool on key performance metrics; and describe tool comparisons. Distinct from previous reviews that have typically combined diverse surgical and non-surgical populations and focused primarily on prognostic utility, this review specifically targets older adults undergoing major intracavitary surgery and extends the evidence summary to encompass all six key metrics of health assessment tool evaluation, including pragmatic factors and clinical impact.

## Methods

The JBI methods and PRISMA-ScR reporting recommendations were followed^33,34^. The completed PRISMA-ScR checklist can be found in *supplementary methods 1*. The protocol was prospectively registered in the Open Science Framework on 30 January 2022 (https://osf.io/ukhnr) and published^35^, with amendments described in *supplementary methods 2*.

### Definitions

Contemporary measurement science terminology was used (*supplementary methods 3*)^36–42^. The term ‘performance metrics’ refers to all relevant metrics assessing how health measurement tools function, grouped into three areas: measurement properties, clinical impact, and pragmatic factors. Measurement properties include validity and reliability^36,37^. Construct validity assesses how well a tool represents the intended construct. Concurrent criterion validity evaluates the degree to which a tool's diagnostic results match a ‘gold standard’ test at the same time point. Predictive validity measures the accuracy of a tool in predicting future outcomes. Reliability examines the consistency of a tool's measurements. Clinical impact evaluates how tools influence care decisions or expected treatment effects^43^. Pragmatic factors, defined by the PAPERS framework^41^, address practical implementation, grouped as acceptability, ease of use, compatibility, and usefulness.

Each performance metric's type of investigation was classified based on study design features. For predictive validity and concurrent criterion, exploratory *versus* confirmatory designs were noted, reflecting the certainty of conclusions (*supplementary methods 3*). For clinical impact and pragmatic factors, classification was based on the domain assessed, not conclusion certainty.

### Eligibility criteria

Detailed criteria are provided in *supplementary methods 4*, with elaboration tables in *supplementary methods 5*. Studies evaluating performance metrics of frailty assessment tools for adults aged ≥ 65 years undergoing major intracavitary non-cardiac surgery were included. This review focused on tools for diagnosis, prognosis, or clinical decision-making applied clinically in the preoperative setting by non-geriatrics-trained clinicians. Studies had to report on a performance metric. Exclusions were studies using frailty only as a covariate, assessing single domains (for example, grip strength), relying solely on laboratory/radiology values, or non-clinical applications (for example, administrative use). Studies without full-text were also excluded.

### Search strategy and information sources

A comprehensive search strategy was co-developed with an expert information specialist and peer reviewed (PRESS checklist). The MEDLINE, Embase, Cochrane Central Register of Controlled Trials (CENTRAL), and Cumulative Index to Nursing and Allied Health Literature (CINAHL) databases were searched initially from 2005 to 13 October 2021, with updates on 19 July 2023 and 18 May 2026, and no language restrictions. The 2005 start date reflects the first use of frailty as a title word in the surgical literature. Detailed strategies are in *supplementary methods 6*. The grey literature was not searched. Reference lists of included studies were screened.

### Selection of sources of evidence

The Covidence platform was used for two-stage citation screening (title/abstract and full-text) with dual independent reviewers (KJ, CS, AM, SK, VBA, MD, BY, AP, AS, and VK). A piloted form and calibration ensured consistency (*supplementary methods 7*). Disagreements were resolved through discussion or by a third reviewer (TRC). The selection process is reported in a PRISMA 2020 flow diagram (*Fig. S1*).

### Data charting

Two independent reviewers (KJ, CS, AM, SK, VBA, MD, BY, AP, AS, and VK) charted study/population characteristics, tool characteristics, performance metrics, and comparisons. For studies reported in multiple records, the most complete or recent information was charted. Piloted electronic forms with training and calibration ensured consistency. Discrepancies were resolved through discussion or by a third reviewer (TRC). The process is detailed in *supplementary methods 7*; detailed data charting tables are presented in *supplementary methods 8*.

### Synthesis of results

Frailty tools were catalogued in tables, quantified by the number of studies and participant counts and visualized by circle packing and stacked bar charts. Study setting (emergency, benign, oncology), surgery type (for example, colorectal, gynaecology, urology), and studied performance metrics were visualized with circular heat maps. For each performance metric, type of investigation was classified based on the definitions above and summarized in a tabular and regular heat map. Predicted outcomes were tabulated by study counts. Comparisons between tools, including metrics, reference standards, and statistical comparisons, were summarized (as detailed in *supplementary methods 9*). Tool content and administration characteristics (for example, delivery, language, frailty model) were collated in tables. To avoid overcrowding and aid interpretation, visual summaries emphasized the 12 most studied tools (≥ 4 studies or 5644 participants, covering 50% of the largest tool's participant count). A summary of key findings was produced as a descriptive synthesis drawing from the above outputs highlighting standout tools based on the extent of study, comparisons, and unique tool features.

## Results

### Selection of evidence sources

Database searches yielded 12 272 records, with no additional citations identified manually (*Fig. S1*). In all, 164 records describing 160 unique studies met the inclusion criteria. Four studies were each reported in two publications, which were consolidated. In addition, five systematic reviews with meta-analyses of relevant outcomes per tool were included; individual studies from these reviews were considered if eligible.

### Characteristics of individual sources of evidence

Study characteristics are summarized in *Table S1*. The median study size was 187 participants, with most studies being single-centre studies (74%) and using a prospective cohort design (68%). Five studies spanned multiple countries, with the US as the most common location (32 studies).

### Frailty assessment tools and extent of supporting data

Across 160 studies, 35 frailty assessment tools were investigated (*Table 1* and *Table S2* for individual studies). Study and participant counts for each tool are summarized in *Fig. 1*. Most tools (69%) appeared in three or fewer studies, with 40% of tools examined in only one study. The Clinical Frailty Score (CFS) had the most studies (41), with data from 9637 participants. Seven tools were studied in over 10 studies. The Risk Analysis Index–Clinical score (RAI-C) had the largest participant count (11 287) from three large studies.

**Figure 1:**
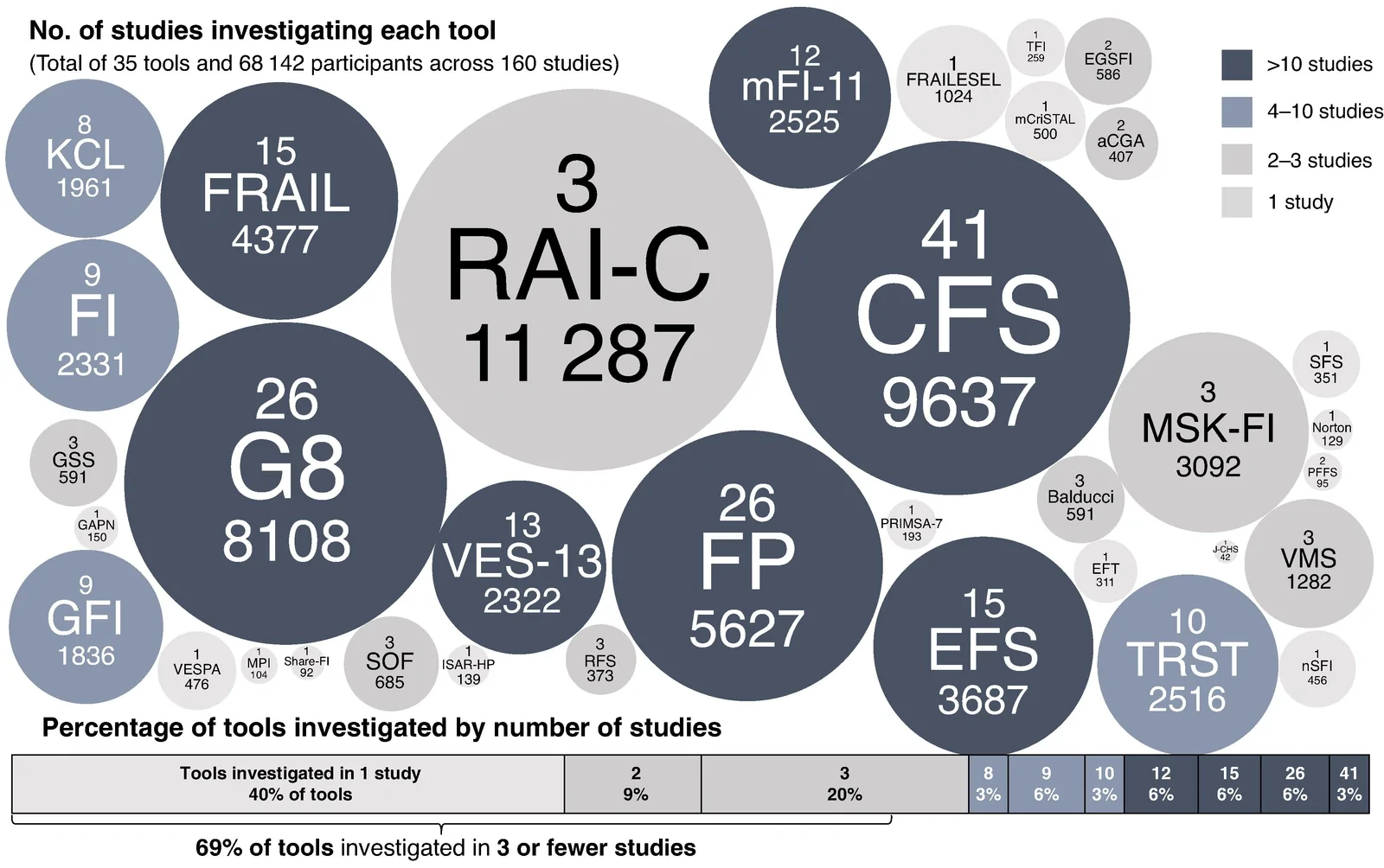
Frailty assessment tools with study and participant counts The number of studies and the number of participants are presented at the top and bottom, respectively, of the symbols for each tool. Colour categories emphasize the number of studies per tool and the circle area is proportional to the sum of participants for a tool across studies. Bar length corresponds to the percentage of tools investigated with a specific number of studies. Tool name abbreviations are provided; refer to *Table 1* for tool names in full.

**Table 1:** Frailty assessment tools with study and participant counts (*n* = 35 tools)

Frailty tool	Full name	No. studies	No. participants
aCGA	Abbreviated Comprehensive Geriatric Assessment	2	407
Balducci	Balducci frailty criteria	3	591
CFS	Clinical Frailty Scale	41	9637
EFS	Edmonton Frail Scale	15	3687
EFT	Essential Frailty Toolset	1	311
EGSFI	Emergency General Surgery Frailty Index	2	586
FI	Frailty Index	9	2331
FP	Fried Frailty Phenotype	26	5627
FRAIL	FRAIL Scale	15	4377
FRAILESEL	Frailty and Emergency Surgery in the Elderly	1	1024
G8	Geriatric 8	26	8108
GAPN	Geriatric Assessment in Partial Nephrectomy	1	150
GFI	Groningen Frailty Indicator	9	1836
GSS	Geriatric Status Scale	3	591
ISAR-HP	Identification of Seniors At Risk–Hospitalized Patients	1	139
J-CHS	Japanese version of the Cardiovascular Health Study	1	42
KCL	Kihon Checklist	8	1961
mCriSTAL	Modified Criteria for Screening and Triaging to Appropriate Alternative Care	1	500
mFI-11	Modified Frailty Index–11 item	12	2525
MPI	Multidimensional Prognostic Index	1	104
MSK-FI	Memorial Sloan Kettering–Frailty Index	3	3092
Norton	Norton Score	1	129
nSFI	New Surgical Frailty Index	1	456
PFFS	Pictorial Fit Frail Scale	2	95
PRISMA-7	PRISMA-7 questionnaire	1	193
RAI-C	Risk Analysis Index–Clinical	3	11 287
RFS	Robinson Frailty Score	3	373
SFS	Simplified Frailty Score	1	351
SHARE-FI	SHARE-FI	1	92
SOF	Study of Osteoporotic Fractures index	3	685
TFI	Tilburg Frailty Indicator	1	259
TRST	Triage Risk Screening Tool	10	2516
VES-13	Vulnerable Elders Survey	13	2322
VESPA	Vulnerable Elderly Surgical Pathways and Outcomes Assessment	1	476
VMS	Veiligheidsmanagementsysteem (Dutch national Safety Management System)	3	1282

Specific studies for each of the frailty assessment tools are listed in *Table S2*, with full publication details provided in the supplementary material.

### Frailty assessment tools across settings and surgical types

Tools were most studied in oncology (29 of 35 tools; *Fig. S2* and *Table S3*). In oncology, the CFS and Geriatric 8 (G8) had the most studies; in benign settings, the Frailty Phenotype (FP) had the most studies; and in emergency surgery, the CFS had the most studies.

For surgical types, general, gastrointestinal, or colorectal surgery had the greatest number of tools studied, each with 24 tools studied (*Fig. S2* and *Table S4*). Only the CFS, G8, FP, Modified Frailty Index–11 item, and Frailty Index had data across all surgical types.

### Performance metrics and type of investigation

Performance metrics assessed for each tool are presented in *Fig. 2* and *Table S5*, with the type of investigation for each performance metric shown in *Fig. 3* and *Table S6*.

**Figure 2:**
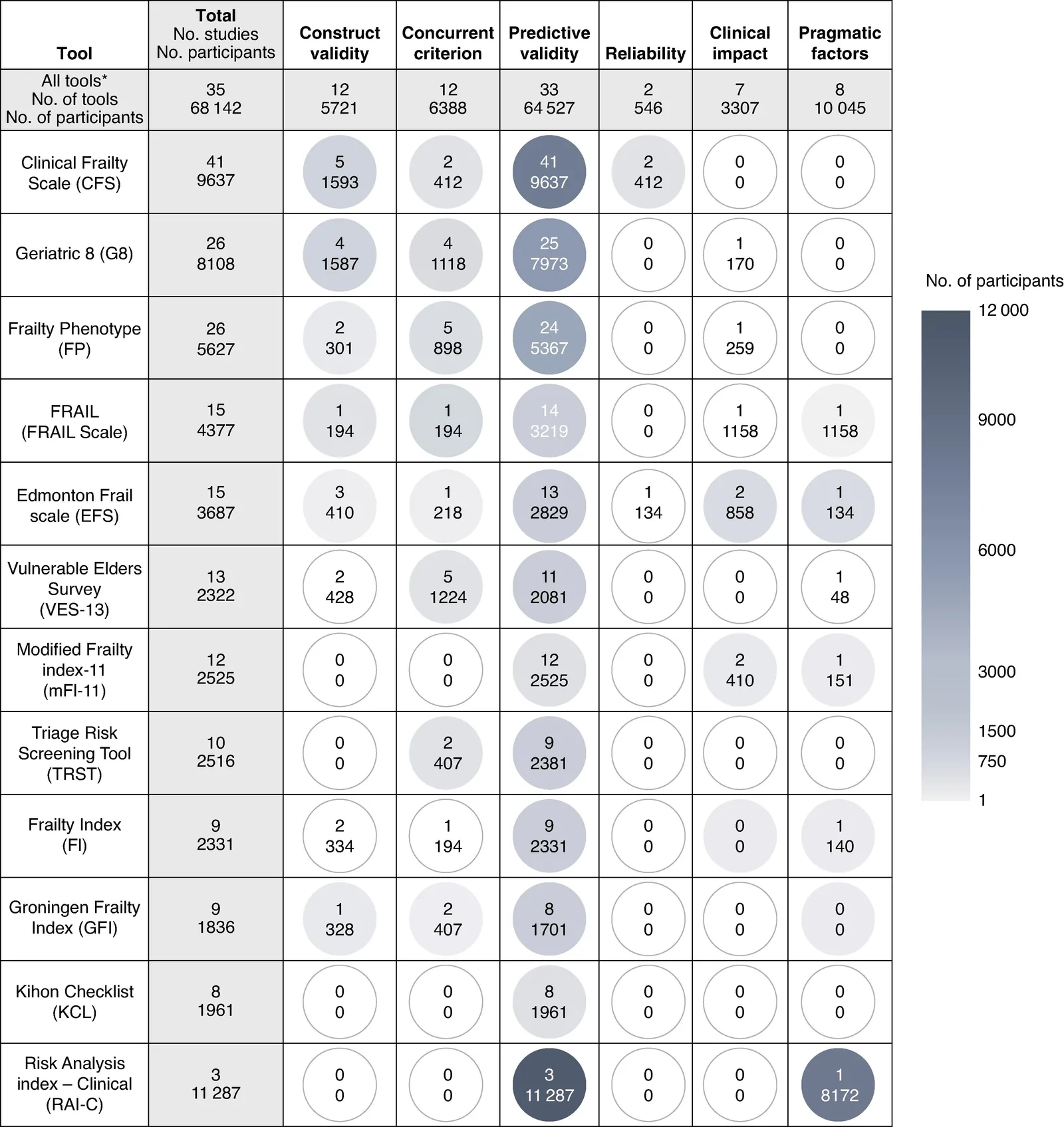
Performance metrics of frailty assessment tools Study and participant counts for each performance metric of the frailty assessment tools are presented (top and bottom of each cell, respectively). The colour scale indicates the number of participants, highlighting the extent of data available for each metric and tool. This visualization focuses on the 12 most extensively investigated tools; additional tools are provided in *Table S5*. *The all tools row includes the total number of tools and participants assessed within each metric, encompassing both visualized and excluded tools not meeting criteria for visualization.

**Figure 3:**
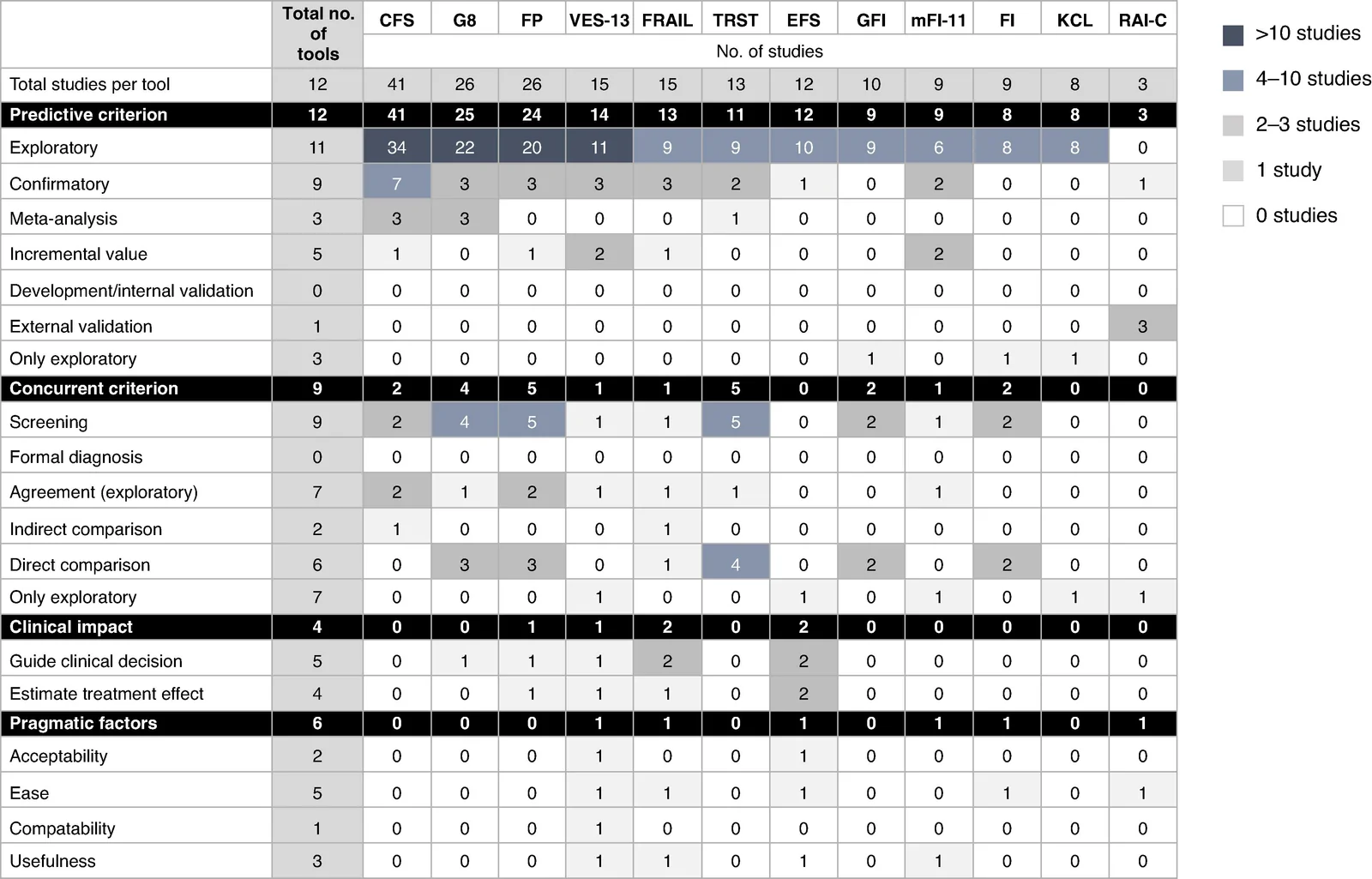
Type of investigation of performance metrics Study counts for frailty assessment tools categorized by type of investigation by performance metric. Colour categories represent the number of studies. This visualization focuses on the 12 most extensively investigated tools; additional tools are provided in *Table S6*. Tool name abbreviations are provided; refer to *Table 1* for tool names in full.

From these four figures and tables, when quantifying by performance metrics, predictive validity was most examined (33 of 35 tools). Among the 12 most investigated tools, nine used confirmatory designs, three included meta-analyses, five assessed incremental value (the added predictive contribution of the frailty tool beyond established risk factors), and one conducted external validation. The most common predicted outcomes were any complication (29 tools), death (26 tools), and readmission (18 tools); geriatric-specific outcomes (for example, delirium, function, mood, cognition) were assessed in only 12 tools (*Table S7*). Construct validity was studied in 12 tools. Concurrent criterion was assessed in 12 tools, with most studies for FP (5 studies), Vulnerable Elders Survey-13 (VES-13; 5 studies), and G8 (4 studies). All tools were used as screening tools, and only six tools underwent a direct comparison with a reference standard. Reliability was assessed in only two tools, and clinical impact was studied in seven tools, but none in randomized trials. Pragmatic factors were examined in eight tools, with ‘ease of use’ being the most common (studied for 5 tools).

From the same four figures and tables (*Figs 2*, *3* and *Tables S5* and *S6*), when assessed by tool, the CFS (a clinician-administered ordinal severity scale (1–9) based on the cumulative deficit model) has data on four of six performance metrics, including seven confirmatory studies, three meta-analyses, incremental validity, and reliability, but lacked data on pragmatic factors, and clinical impact. The EFS, a multidimensional nine-domain assessment yielding an ordinal frailty severity score (0–17), was the only tool with data on all six performance metrics. FRAIL, a brief, self-administered five-item phenotype-based questionnaire (scored 0–5), has data on five metrics, including confirmatory predictive validity, incremental value, clinical impact and pragmatic factors, but lacked reliability data. The G8 (an eight-item oncology-focused screening questionnaire scored 0–17), FP (a five-criterion phenotypic classification requiring dynamometry and timed walk), and the VES-13 (a self-administered 13-item vulnerability survey scored 0–10) were among the tools with the most studies, each with confirmatory designs for concurrent criterion. The G8 included three meta-analyses for predictive validity and the VES-13 had data on all four pragmatic factors. RAI-C, a composite frailty index derived from clinical or administrative records, had high participant counts but was limited to predictive validity and ease of use.

### Comparisons between frailty assessment tools

Of the 35 tools, 26 were compared with at least one other tool (*Table S8*). Twenty-two tools were compared on predictive validity, and 14 were compared for concurrent criterion. Direct comparisons were conducted for 14 tools, each involving one to four studies. G8 had the most direct comparisons (four studies). No comparisons included a reference standard by expert geriatrics clinicians.

### Characteristics of individual frailty assessment tools

This synthesis highlights the most studied tools (*Tables S9* and *S10*). Most are questionnaires, with three relying on physical performance and the CFS relying on clinical judgment. The CFS and EFS use ordinal scales to grade frailty, whereas most tools use thresholds for positive results. Four tools are available in languages other than English, and most were developed for non-surgical populations. Several tools lack a formal frailty conceptual model but cover multiple geriatric domains. Five tools are accessible online. The CFS and EFS require basic training, which is offered free online, and the EFS requires licencing. Although most tools lack specified administration times, some can be self-administered. The FP, EFS, and Frailty Index need special equipment, but a self-reported EFS version has been studied^44^.

The CFS is grounded in the cumulative deficit model of frailty and yields a nine-point ordinal scale (1–9), ranging from very fit to terminally ill, with scores ≥ 5 conventionally denoting frailty (*Table S9*). Assessment integrates clinician judgment across domains of mobility, energy, physical activity, daily function, cognition, and co-morbidity using structured prompting questions and a visual pictographic aid. It requires clinician administration and is not suitable for self-completion; free web-based training is available. Potential clinical utility includes ordinal frailty severity grading to support preoperative risk stratification, prognostic communication, and goals-of-care planning across diverse surgical settings.

The EFS evaluates nine geriatric domains: cognitive performance (clock-drawing test), general health status, functional independence in instrumental and basic activities of daily living, social support, medication use, nutrition, mood, continence, and functional mobility (Timed Up and Go Test; *Table S9*). Items are summed to a score of 0–17, categorized into five frailty severity strata ranging from not frail to severely frail. Administration requires in-person performance and cognitive testing by a trained assessor; a licence is required and free web-based training is available. A validated self-reported version omitting the performance-based domains has been described^44^. Potential clinical utility includes multidimensional frailty severity grading, the identification of specific modifiable deficits for targeted prehabilitation or co-management, and serial frailty monitoring.

The FRAIL scale operationalizes the phenotype model through five self-reported items, namely Fatigue, Resistance (difficulty climbing one flight of stairs), Ambulation (difficulty walking one block), Illness (≥ 5 co-morbidities), and Loss of weight (> 5% over the previous year), summed to a score of 0–5, with scores of ≥ 3 indicating frailty and scores of 1–2 indicating prefrailty (*Table S9*). No equipment or specialized training is required, and the FRAIL scale may be self-administered.

The G8 is an eight-item questionnaire comprising items on appetite, weight loss, mobility, neuropsychological problems, body mass index, number of medications, self-rated health, and age, summed to a score of 0–17, with scores ≤ 14 indicating a need for further comprehensive geriatric assessment (*Table S9*).

The VES-13 evaluates 13 items spanning age, self-rated health, physical function limitations, and difficulty with activities of daily living, yielding a score of 0–10, with scores ≥ 3 indicating vulnerable elder status (*Table S9*). Both the G8 and VES-13 are self-administered and require no specialized training; both are commonly used in oncology settings. All three tools (FRAIL scale, VES-13, and G8) yield binary frailty classifications rather than ordinal severity scores; potential clinical utility includes rapid screening and triage for further geriatric evaluation.

The FP is based on the Fried phenotype model and classifies frailty according to five criteria: unintentional weight loss, self-reported exhaustion, low physical activity (standardized questionnaire), slow gait speed (timed ∼4.5-m (15-foot) walk), and weak grip strength (handheld dynamometer; *Table S9*). Each criterion is dichotomously scored; the presence of three or more criteria indicates frailty, with the presence of one to two criteria indicating prefrailty. Assessment requires specialized equipment and a trained evaluator, posing practical barriers to implementation in routine clinical settings. Potential clinical utility includes use as a research-grade phenotypic reference standard and in comparative validity studies evaluating other tools.

The RAI-C is a composite frailty index derived from clinical or administrative records, assessing domains including functional status, cognitive impairment, co-morbidity burden, symptom burden, and weight loss (*Table S9*). Scores are summed to yield a continuous severity estimate, with higher values indicating greater frailty burden. Unlike most other tools in this review, the RAI-C does not require direct patient interaction and can be calculated from existing clinical data. Potential clinical utility includes large-scale preoperative risk stratification and integration with administrative data sets for population-level frailty surveillance.

## Discussion

This scoping review identified 160 studies investigating 35 frailty assessment tools used before major intracavitary non-cardiac surgery in older adults. This review provides a comprehensive summary of evaluated performance metrics and the type of investigation, along with individual characteristics of these frailty assessment tools, focusing the synthesis on the 12 most extensively studied tools by study and participant counts. Although some tools were well studied, with up to 41 studies and high participant counts, 40% were evaluated in only one study, and 69% were evaluated in fewer than three studies. Predictive validity was commonly assessed, but concurrent criterion and construct validity were studied in less than half the tools, and reliability, clinical impact, and pragmatic factors received little attention. Confirmatory studies were uncommon.

Five tools stood out based on robust investigation and unique features: the CFS, EFS, FRAIL, G8, and VES-13 (*Box 1*). The CFS has the second-highest participant count and the most extensive study count, covering four of six performance metrics, including confirmatory designs and meta-analyses of predictive validity, and is one of two tools with data on reliability. Predictive validity has been assessed for most outcomes, including geriatric-specific outcomes, and across diverse settings and surgical types. Comparisons with other tools exist in both concurrent and predictive validity, with two direct comparison studies. The CFS is based on the cumulative deficits model and yields an ordinal scale for grading frailty severity. It is clinician administered, requiring basic training and prompting questions, making it unsuitable for self-administration. Free web-based training is available. The lack of data on pragmatic factors and clinical impact is an important gap.

Box 1 Summary of Key Findings of Most Extensively Investigated Tools **Clinical Frailty Scale**

- Most studied by study count and second highest studied by participant count
- Investigated across four of six performance metrics, covering a wide range of outcomes, settings, and surgical types
- Operationalized as an ordinal scale to grade frailty severity
- Basic web-based training required; no special equipment
- Lack of data on clinical impact and pragmatic factors is a gap

**Edmonton Frail Scale**

- Only tool investigated across all six performance metrics; across all settings and surgical types (except thoracics)
- Important metrics of incremental value, clinical impact, and pragmatic factors assessed, but in smaller studies
- Operationalized as an ordinal scale to grade frailty severity
- Provides a multidimensional assessment, but relies on performance and cognitive testing, which may pose barriers in some environments
- Basic web-based training and license required
- A self-reported version has been developed, but requires further study

**FRAIL scale, Geriatric 8 (G8), and Vulnerable Elders Survey (VES-13)**

- User-friendly questionnaires suitable for self-administration; no training or special equipment needed
- Use threshold scoring, limiting ability to grade frailty severity
- FRAIL scale investigated across five of six performance metrics, including incremental value, clinical impact, and pragmatic factors; studied across all settings, and most surgical types
- G8 and VES-13 predominantly studied in oncology setting and, despite high study and participant counts, data on four of six performance metrics

**Frailty Phenotype and Risk Analysis Index—Clinical**

- High number of studies and participants
- Data limited to few performance metrics, representing an important gap
- Frailty Phenotype requires special equipment and training for administration.

The EFS is the only tool with data on all six metrics, including incremental value, clinical impact, and pragmatic factors, all of which are critical to meaningful clinical implementation. However, several metrics are assessed in single studies with few participants, indicating a need for further study. The EFS spans most settings and surgical types and provides a multidimensional assessment of geriatric domains, yielding an ordinal scale of frailty severity. Basic, free, web-based training resources are available, and a licence is needed. However, the reliance of the EFC on performance and cognitive testing may pose a barrier to its implementation, although a self-reported version shows promise to overcome this barrier pending further study^44^.

The FRAIL tool covers five of six metrics, including incremental value, clinical impact, and pragmatic factors, and has been studied across most settings and surgical types. It is a simple questionnaire that can be self-administered and is based on the phenotype model of frailty. Similarly, the G8 and VES-13 are straightforward questionnaires that can be self-administered and are commonly used in oncology, but they cover only three metrics. Although these three tools are easy to use, they provide a binary assessment rather than ranking frailty severity.

In contrast, despite high study and participant counts, the FP and RAI-C are limited to three and two metrics, respectively. The FP requires special equipment and trained clinicians, posing implementation barriers, whereas the RAI-C’s narrow scope of data warrants further study before implementation is considered.

This review provides a catalogue of frailty assessment tools relevant to perioperative care. Current guidelines often recommend frailty assessments but lack specific tool recommendations^6^. Perioperative teams can use this review to identify tools aligned with local needs and priorities, such as context-specific use (for example, emergency or thoracic surgery) or broad applicability across settings and surgical types. Teams seeking comprehensive tools may consider the CFS or EFS for their robust data across multiple metrics and settings, provided training resources are available. Simpler tools like the FRAIL scale offer easy self-administration and may assist in triaging patients for further assessment by geriatric specialists.

This scoping review also informs priority setting for researchers. With 35 existing tools, the development of new tools should be avoided unless addressing a specific gap. Instead, efforts should focus on advancing well-studied tools by addressing gaps such as confirmatory studies for predictive validity, direct comparisons for concurrent criterion, and providing data on reliability, clinical impact, and pragmatic factors. Clinical impact is critical because it directly influences patient care. Investigating clinical impact and pragmatic factors would directly address some barriers to implementing routine preoperative frailty assessment, such as confidence in a specific tool, time for administration, relevant skills and training needs, application in virtual/telemedicine, approach to responding to frailty assessment results, and strategies for acting on assessment results^7,12,13^. Methodological guidance, such as the COSMIN^37^ and STRATOS^42^ initiatives, TRIPOD^38^ and STARD^39^ guidance, PROGRESS series^40^, and PAPERS framework^41^, provides a pathway for higher-quality study designs to address these gaps^36–42^. Strong examples include assessment of incremental prognostic value of frailty tools, inter-rater reliability, and cross-cultural measurement properties; the use of validated tools for assessing feasibility; and prospective intervention studies examining clinical impact of frailty assessment^45–48^.

Further, this scoping review highlights a significant gap in tool-specific quantitative evidence synthesis across all performance metrics. Currently, only three tools (CFS, G8, and Triage Risk Screening Tool) have had tool-specific quantitative meta-analyses, all limited to predictive validity^49–51^. One meta-analysis^18^ examined tools individually but still focused solely on predictive validity. Another meta-analysis^52^ included subgroup analyses by tool but identified only 11 tools. A well-conducted meta-analysis of the FRAIL tool was conducted^53^, but again focused exclusively on predictive validity. In addition, many reviews reported statistics like pooled odds ratios, which are less informative for predictive validity than recommended metrics such as discrimination, calibration, and likelihood ratios^38^. Although meta-analysis of outcomes does not align with scoping review objectives, this review identifies tools amenable to quantitative evidence synthesis. Future efforts could prioritize tool-specific meta-analyses of performance metrics for the most extensively studied tools, with subgroup analyses by setting and surgical type. Candidates for such analysis include the CFS, EFS, FRAIL, G8, and VES-13.

Adhering to standard methodological guidelines for scoping reviews has maintained the quality of this study. Further, this scoping review is the first to include all relevant performance metrics, including clinical impact and pragmatic factors, and makes use of a novel framework for assessing pragmatic factors (PAPERS)^41^. In addition, by focusing specifically on major intracavitary non-cardiac surgery, this review examines a more clinically homogeneous population than previous work, which has typically combined diverse surgical and non-surgical contexts, thereby providing more directly actionable evidence for perioperative implementation efforts. However, limitations exist. Due to predefined eligibility criteria targeting clinically applied tools in the preoperative setting for major intracavitary non-cardiac surgeries, there is a possibility that potentially valuable frailty assessment tools, albeit unstudied in this context, were not captured. Given the objectives of scoping reviews, risk of bias assessment and quantitative meta-analysis are not performed; thus, this study cannot draw conclusions regarding summary effect sizes or risk of bias concerning specific performance metrics across tools. This review focused on surgical types with similar recovery trajectories, specifically major intracavitary non-cardiac surgery, so the findings cannot be applied to other surgery types.

This scoping review catalogues all clinically applied preoperative frailty assessment tools investigated in older adults undergoing major intracavitary non-cardiac surgeries, providing a comprehensive evaluation of their extent of investigation across all key performance metrics. Although 35 tools were identified, most had limited evaluation, with only a few demonstrating robust investigation. These findings offer a resource for perioperative teams to select tools aligned with local needs, and for research team to identify key priorities. Future research efforts should focus on high-quality confirmatory studies and tool-specific meta-analyses across all performance metrics, particularly clinical impact, to improve the implementation of frailty assessment tools in clinical practice.

## Supplementary Material

zrag129_Supplementary_Data

## Data Availability

All data supporting the findings of this study are included within the article and its supplementary files. Raw data extraction tables can be obtained from the corresponding author upon request.
