# Supplementary material for: Frailty assessment tools for preoperative evaluation of older adults: scoping review
Source: BJS Open. 2026 Oct 23;10(5):zrag129. doi: 10.1093/bjsopen/zrag129 (PMC13692690; doi:10.1093/bjsopen/zrag129)
Supplement: Supplementary file 1 [file zrag129_supplementary_data.docx]

**Frailty assessment tools for preoperative evaluation of older adults: scoping review**

Tyler R Chesney MD MSc^1,2,^; Camilla L Wong MD MHSc^2,3^; Andrea C Tricco PhD^4,5^; Duminda N Wijeysundera MD PhD^6,7^; Marisa Louridas MD PhD^1^; Kesikan Jayaraj BScN MD^8^; Claire Sethuram MD^9^; Amanda Mac^10^; Shawn Khan MD MEng^11^; Victoria B Anthes MD^10^; Madeline McDonald MD MSc^12^; Betty Yibrehu MD^1^; Amanda Poxon MD^10^; Anas Samman MD^10^; Valerie Kim MD^10^; Karim S Ladha MD MSc^6,7^; Teruko Kishibe MISt^2^; Samuel Dubé MD^13^; Martine Puts RN PhD^14^; Shabbir MH Alibhai MD MSc^7,15^; Julian F. Daza MD^1,7^

^1^Department of Surgery, Division of General Surgery, University of Toronto, Toronto, ON, Canada

^2^Li Ka Shing Knowledge Institute, St. Michael's Hospital, Unity Health Toronto, Toronto, ON, Canada

^3^Division of Geriatric Medicine, St. Michael’s Hospital, Unity Health Toronto, Toronto, ON, Canada

^4^Epidemiology Division & Institute of Health Policy, Management, and Evaluation, University of Toronto, Toronto, ON, Canada

^5^Knowledge Translation Program, Li Ka Shing Knowledge Institute, St Michael's Hospital, Unity Health Toronto, Toronto, ON, Canada

^6^Department of Anesthesiology and Pain Medicine, St. Michael’s Hospital and University of Toronto, Toronto, ON, Canada

^7^Institute of Health Policy, Management and Evaluation, University of Toronto, Toronto, ON, Canada

^8^Department of Anesthesiology, McMaster University, Hamilton, ON, Canada

^9^Department of Internal Medicine, University of Ottawa, Ottawa, ON, Canada

^10^Temerty Faculty of Medicine, University of Toronto, Toronto, ON, Canada

^11^Department of Emergency Medicine, Cambridge Memorial Hospital, Cambridge, ON, Canada

^12^Department of Surgery, McMaster University, Hamilton, ON, Canada

^13^Division of Gynecologic Oncology, Université de Montréal, Montreal, Quebec, Canada

^14^Lawrence S. Bloomberg Faculty of Nursing, University of Toronto, Toronto, ON, Canada

^15^Department of Medicine, University Health Network and University of Toronto, Toronto, ON, Canada

**Corresponding author.** Tyler R Chesney. St. Michael's Hospital, Cardinal Carter North, Room 16-064, 30 Bond St,Toronto, ON, M5B 1W8

**Supplementary Materials - Index**

| **Supplementary Methods** |  |
| --- | --- |
| 1. Preferred Reporting Items for Systematic reviews and Meta-Analyses extension for Scoping Reviews (PRISMA-ScR) Checklist | *pag. 4* |
| 2. Protocol Amendments | *pag. 6* |
| 3. Definitions of performance metrics and type of investigation. | *pag. 10* |
| 4. Detailed eligibility criteria | *pag. 14* |
| 5. Eligibility criteria explanation and elaboration tables | *pag. 15* |
| 6. Search Strategies | *pag. 19* |
| 7. Details on selection of sources of evidence and data charting | *pag. 26* |
| 8. Data charting form explanation and elaboration tables | *pag. 28* |
| 9. Details on synthesis of results for type of investigation and comparison | *pag. 36* |
| **Supplementary Figures and Tables** |  |
| Figure S1. PRISMA 2020 Flow Diagram for Record Identification | *pag. 37* |
| Table S1. Characteristic of Supporting Studies | *pag. 38* |
| Table S2. Frailty assessment tools and investigating studies | *pag. 40* |
| Figure S2. Frailty Assessment Tools by Setting and Surgical Type | *pag. 53* |
| Table S3. Frailty Assessment Tools by Setting | *pag. 54* |
| Table S4. Frailty Assessment Tools by Surgery Type | *pag. 55* |
| Table S5. Performance Metrics of Frailty Assessment Tools | *pag. 57* |
| Table S6. Type of Investigation of Measurement Properties | *pag. 59* |
| Table S7. Number of Studies Reporting Outcomes for Predictive validity | *pag. 62* |
| Table S8. Comparisons Between Frailty Assessment Tools | *pag. 66* |
| Table S9. Brief Description of Tool Characteristics | *pag. 67* |
| Table S10. Frailty assessment tool characteristics | *pag. 69* |
| **References** | *pag. 89* |

**Supplementary Methods**

# Supplement Methods 1. Preferred Reporting Items for Systematic reviews and Meta-Analyses extension for Scoping Reviews (PRISMA-ScR) Checklist

| **SECTION** | **ITEM** | **PRISMA-ScR CHECKLIST ITEM** | **REPORTED ON PAGE #** |
| --- | --- | --- | --- |
| **TITLE** | | | |
| Title | 1 | Identify the report as a scoping review. | 1 |
| **ABSTRACT** | | | |
| Structured summary | 2 | Provide a structured summary that includes (as applicable): background, objectives, eligibility criteria, sources of evidence, charting methods, results, and conclusions that relate to the review questions and objectives. | 2 |
| **INTRODUCTION** | | | |
| Rationale | 3 | Describe the rationale for the review in the context of what is already known. Explain why the review questions/objectives lend themselves to a scoping review approach. | 2 |
| Objectives | 4 | Provide an explicit statement of the questions and objectives being addressed with reference to their key elements (e.g., population or participants, concepts, and context) or other relevant key elements used to conceptualize the review questions and/or objectives. | 2 |
| **METHODS** | | | |
| Protocol and registration | 5 | Indicate whether a review protocol exists; state if and where it can be accessed (e.g., a Web address); and if available, provide registration information, including the registration number. | 2 |
| Eligibility criteria | 6 | Specify characteristics of the sources of evidence used as eligibility criteria (e.g., years considered, language, and publication status), and provide a rationale. | 5 |
| Information sources* | 7 | Describe all information sources in the search (e.g., databases with dates of coverage and contact with authors to identify additional sources), as well as the date the most recent search was executed. | 5 |
| Search | 8 | Present the full electronic search strategy for at least 1 database, including any limits used, such that it could be repeated. | 5 |
| Selection of sources of evidence† | 9 | State the process for selecting sources of evidence (i.e., screening and eligibility) included in the scoping review. | 5 |
| Data charting process‡ | 10 | Describe the methods of charting data from the included sources of evidence (e.g., calibrated forms or forms that have been tested by the team before their use, and whether data charting was done independently or in duplicate) and any processes for obtaining and confirming data from investigators. | 5 |
| Data items | 11 | List and define all variables for which data were sought and any assumptions and simplifications made. | 5 |
| Critical appraisal of individual sources of evidence§ | 12 | If done, provide a rationale for conducting a critical appraisal of included sources of evidence; describe the methods used and how this information was used in any data synthesis (if appropriate). | NA |
| Synthesis of results | 13 | Describe the methods of handling and summarizing the data that were charted. | 5 |
| **RESULTS** | | | |
| Selection of sources of evidence | 14 | Give numbers of sources of evidence screened, assessed for eligibility, and included in the review, with reasons for exclusions at each stage, ideally using a flow diagram. | 6 |
| Characteristics of sources of evidence | 15 | For each source of evidence, present characteristics for which data were charted and provide the citations. | 6 |
| Critical appraisal within sources of evidence | 16 | If done, present data on critical appraisal of included sources of evidence (see item 12). | NA |
| Results of individual sources of evidence | 17 | For each included source of evidence, present the relevant data that were charted that relate to the review questions and objectives. | 6 |
| Synthesis of results | 18 | Summarize and/or present the charting results as they relate to the review questions and objectives. | 6 |
| **DISCUSSION** | | | |
| Summary of evidence | 19 | Summarize the main results (including an overview of concepts, themes, and types of evidence available), link to the review questions and objectives, and consider the relevance to key groups. | 9 |
| Limitations | 20 | Discuss the limitations of the scoping review process. | 10 |
| Conclusions | 21 | Provide a general interpretation of the results with respect to the review questions and objectives, as well as potential implications and/or next steps. | 10 |
| **FUNDING** | | | |
| Funding | 22 | Describe sources of funding for the included sources of evidence, as well as sources of funding for the scoping review. Describe the role of the funders of the scoping review. | 11 |

JBI = Joanna Briggs Institute; PRISMA-ScR = Preferred Reporting Items for Systematic reviews and Meta-Analyses extension for Scoping Reviews.

* Where *sources of evidence* (see second footnote) are compiled from, such as bibliographic databases, social media platforms, and Web sites.

† A more inclusive/heterogeneous term used to account for the different types of evidence or data sources (e.g., quantitative and/or qualitative research, expert opinion, and policy documents) that may be eligible in a scoping review as opposed to only studies. This is not to be confused with *information sources* (see first footnote).

‡ The frameworks by Arksey and O’Malley (6) and Levac and colleagues (7) and the JBI guidance (4, 5) refer to the process of data extraction in a scoping review as data charting*.*

§ The process of systematically examining research evidence to assess its validity, results, and relevance before using it to inform a decision. This term is used for items 12 and 19 instead of "risk of bias" (which is more applicable to systematic reviews of interventions) to include and acknowledge the various sources of evidence that may be used in a scoping review (e.g., quantitative and/or qualitative research, expert opinion, and policy document).

*From:* Tricco AC, Lillie E, Zarin W, O'Brien KK, Colquhoun H, Levac D, et al. PRISMA Extension for Scoping Reviews (PRISMAScR): Checklist and Explanation. Ann Intern Med. 2018;169:467–473. [doi: 10.7326/M18-0850](http://annals.org/aim/fullarticle/2700389/prisma-extension-scoping-reviews-prisma-scr-checklist-explanation).

# Supplement Methods 2. Protocol Amendments

1. To enhance clarity and alignment with measurement science in medicine, several terminology clarifications were implemented in the final manuscript:^1–10^

- We chose the umbrella term “Performance metrics” to encompass all relevant aspects of health measurement tools used for diagnosis, prognosis, or clinical decisions including measurement properties, clinical impact, and pragmatic factors.
- Measurement properties include a range of assessments including validity and reliability.
- Tools used for diagnosis. When tools are used for diagnosis, criterion validity is assessed by the concurrent criterion, which is the degree by which the tool matches the results of a gold standard test at the same timepoint. As such, we used the term concurrent criterion when discussing tools used for diagnosis and measurement of diagnostic accuracy.
- Tools used for prognosis. When tools are used for prognosis, criterion validity is assessed by predictive validity, which is the accuracy with which the tool predicts a future outcome. As such, we used the term predictive validity when discussing tools used prognosis or predicting a future relevant outcome.
- Clinical impact replaced "Treatment effect" to emphasize the tool's impact rather than specific treatments, encompassing differential treatment effect (e.g., different magnitude of benefit from treatment based on degree of frailty), and guide clinical decision (or clinical decision rule).
- Usability items. This was changed to “Pragmatic Factors” to reflect new recommendations for this domain detailed in the PAPERS framework.^10^ This framework used a Delphi process to strengthen the assessment of pragmatic measures of tools which have previously been described by words like usability, feasibility, ease, etc. The PAPERS framework groups these into four categories: acceptability, ease of use, compatibility, and usefulness. Acceptability includes acceptability to stakeholders, relative advantages over other similar instruments, cost. Ease of use includes ease of completion, language accessibility, burden of training, burden of interpretation, instrument length. Compatibility includes appropriateness for stakeholders. Usefulness includes fit within organizational activities, clinical decision-making guidance. We updated our data charting and synthesis using this framework.

2. Objectives. Prior: *In this scoping review, we will systematically identify the published literature assessing frailty assessment tools in the preoperative clinical setting, and 1) Index the frailty assessment tools that have been developed or evaluated in the preoperative clinical setting, 2) Describe the level of investigation supporting each tool for diagnosis, prognosis or treatment effect estimation, 3) Describe useability in clinical practice of each tool, and 4) Describe direct comparisons between tools.*

- The objectives were rephrased to align with the terminology change indicated in amendment #1.

3. Eligibility criteria

- After piloting eligibility criteria, we added the following surgical types to the exclusions: transplant surgery, bariatric surgery. These were excluded as these are very unique populations which specific risk profiles not generalizable to intracavitary surgery as a whole.

4. Methods. Data Charting. Data Items.

- The geriatric domain “falls” was expanded to include an assessment of either falls or mobility. The domain “physical performance” was clarified to specifically include a physical performance test.
- We clarified the assessment of construct validity during data charting. A tool was considered to have construct validity if it was compared to another tool measuring a similar but distinct concept, such as physical performance or disability, in addition to the initial definition provided in the data charting manual
- Tool Characteristics. Given this information is intrinsic to the tools and not study-dependent, where this information was not available in the manuscripts of included studies, we identified missing information from original development and validations studies, or relevant updated resources.
- Tool Characteristics. We moved “conceptual framework” from the measurement properties section of data charting to tool characteristics. We added a category “multidimensional” to conceptual framework for tools that assessed multiple geriatric domains, but were not explicitly developed based on cumulative deficits or phenotype models.
- Tool Characteristics. Added “index” to scoring methods.
- Tool Characteristics. Added the following data items for data charting: Training required, Administration time, Mode of Administration, Special Equipment, Licence Needed

5. Synthesis of Results. To aid in synthesizing data, gastrointestinal and upper gastrointestinal were grouped together as gastrointestinal for ease of analysis.
6. Synthesis of Results. Type of investigation. The prior analysis plan was updated to clarify levels of investigation for different performance metrics.

- Previously: *Specifically, level of investigation will be ordered* *as development study, validation study, replication study, impact study and systematic review. Studies will be included if they only provide unadjusted or adjusted associations between preoperative frailty (measured using a frailty assessment tool) and a postoperative outcome, but will be ranked as the lowest level of investigation unless formal diagnostic test, prognostic, treatment effect estimate, impact analysis or measurement properties study methodology is used*.
- We changed the term “level of investigation” to “type of investigation” to reflect the study design features examining each performance metric.
- Diagnosis/Concurrent criterion. Based on recommendations for assessment of concurrent criterion or diagnostic test accuracy, tools were classified by purpose as either screening or formal diagnosis, and type of investigation was classified into three levels.^6,11–14^.
  - Agreement (Exploratory). The is an exploratory study design. This includes any of the following: agreement, concordance, correlation test (spearman, kappa, etc); association, univariable (no other variables were included in the statistical model); association, multivariable (other variables were included in the statistical model)
  - Indirect comparison: listing of statistical measures for individual tools, no direct comparison of these tools or no gold standard. This includes: sensitivity/specificity; positive predictive value/negative predictive value; accuracy (statistical measure of proportion of all tests that give the correct result over all results); likelihood ratio; AUC (area under the curve)/ROC (receiver operator characteristic)/AUROC
  - Direct comparison: use of real gold standard as reference standard, or direct comparison of 2 or more index tests (e.g., diagnostic OR); or any of the above with comparison to a gold standard (meaning CGA, Fried Phenotype, Rockwood FI)
- Prognosis/Predictive validity. Based on recommendations for assessment of predictive validity, prognosis tools or prediction models, type of investigation was classified into several levels based on if frailty tool was assessed as a single factor, or as a prognostic model.^5,7,15–17^

Prognostic factor

- Exploratory. This reflected exploratory study designs: no multivariable analysis; multivariable analysis, but using univariable screen or other statistical means for variable selection; multivariable analysis, but design used to filter multiple frailty tools, or variable selection not based on known prognostic factors
- Confirmatory. This reflected confirmatory study designs: frailty tool added to reference or base model
- Incremental value. This is a higher level study type designed to explicitly assess the value of a frailty assessment tool in addition to other known prognostic factors or models. This includes any direct assessment of model performance of a base/reference model compared to performance with addition of frailty tool (e.g., change in overall model fit, change in AUC/discrimination, change in calibration, change in overall predictive performance (e.g., R2, reclassification)).

Prognostic model

- This includes two levels of prognostic model development and validation: 1) model development and/or internal validation, and 2) external validation.
- Changes to Treatment Effect and Usability were reflected in amendment #1 above.
- Systematic reviews with a quantitative meta-analysis of a measurement property of a specific tool was added to the “Type of investigation” section as this represents a higher type of investigation, dependent on the quality of the meta-analysis.

7. Synthesis of Results. Comparisons. Previously: *We will summarise the outcomes of any direct comparisons between tools.*

- We altered this to chart and summarize data on any comparisons. We then grouped these based on comparisons on diagnosis/concurrent criterion, and prognosis/predictive validity. We then charted and summarized if comparisons were direct comparisons, and if a comparison was made to reference standard.
- Indirect comparison: a statistic or effect measure reported for each tool, but no direct statistical comparison between tools diagnostic or prognostic accuracy
- Direct comparison: a direct statistical comparison between two or more tools:
  - e.g., relative sensitivity, relative diagnostic odds ratio (rDOR), McNemar’s test of differences in sensitivity or specificity.^18,19^
  - e.g., comparison or discrimination, calibration, brier score, incremental value, net reclassification improvement.^5,7,15–17^
- Comparison to reference standard. Any assessment using a standard diagnostic process for frailty, documented explicitly to be assessed by a healthcare practitioner or team with geriatric expertise.

8. Synthesis of Results. Visual plots. Previously: *We will create a visual summary (eg, bubble plot) to visually summarise the number of studies and level of investi- gation supporting each tool, stratified by setting (overall, oncology, benign, emergency) and by surgery type.*

- For the final analysis, we chose several visual plots described in the “synthesis of results” resection including circle packing, 100% stacked bar charts, circular heat maps, and regular heat maps.
- For most visual plots, due to the large number of tools (35) the visual plots were too crowded for meaningful interpretation if all tools were included. Therefore, data on all tools were provided in tabular form, and to aid interpretation the visual plots were reserved for the 12 most extensively investigated tools. Given that the aim of the visual plots was to highlight the tools with the greatest extent of available data by study or participant counts, for inclusion in the visual summaries we selected the threshold of a tool having either more than 4 studies or a minimum of 5,644 included participants across studies (representing 50% of the participant count for the tool with the highest number of participants).

# Supplement Methods 3. Definitions of performance metrics and type of investigation.

In developing the research plan for this scoping review, the review team used contemporary measurement science terminology.^1–10^ In general, health measurement tools are any instrument used to measure a health construct, and can include a range of data types from lab values, physical performance measures, to history and physical exam findings, or questionnaires.

**Performance Metrics**

We chose the umbrella term “Performance metrics” to encompass all relevant metrics designed to assess the performance of health measurement tools used for diagnosis, prognosis, or clinical decisions. Performance metrics are assessments of how a health measurement tool functions. These metric can be grouped into three categories: measurement properties, clinical impact, and pragmatic factors.

*Measurement Properties*

Measurement properties assess the performance of a tool, broadly grouped as validity and reliability. Validity reflects how well the tool measures the construct of interest, and reliability reflects how free the tool is from measurement error.^2,3^ Responsiveness, which detects true change over time, was not investigated in this review as it focuses on single preoperative assessments.

Below are brief definitions of measurement properties investigated in this scoping review:

- Construct Validity. Evaluates the internal structure of a measurement tool and reflect how well it represents the construct it is intended to measure (e.g., frailty).
- Concurrent Criterion. When tools are used for diagnosis, criterion validity is assessed by the concurrent criterion, which is the degree by which the tool matches the results of a gold standard test at the same timepoint. As such, we used the term concurrent criterion when discussing tools used for diagnosis and measurement of diagnostic accuracy.
- Predictive Validity. When tools are used for prognosis, criterion validity is assessed by predictive validity, which is the accuracy with which the tool predicts a future outcome. As such, we used the term predictive validity when discussing tools used for prognosis or predicting a future outcome.
- Reliability. This is a statistical assessment of how much the differences in measurements by the tool reflect true differences between patients or differences in the same patient if a true change has occurred. There are several relevant measures of reliability. *Internal consistency* is a statistical assessment of the interrelatedness among items within the same measurement instrument. *Inter-rater and test-retest reliability* are statistical assessments of the degree to which the measurement is the same for patients who have not changed when measured by different raters, or at different times. *Measurement error* is the systematic and random error of scores not due to true changes in the measured construct.

*Clinical Impact*

Clinical impact is an assessment of how a health measurement tool impacts clinical care or outcomes through differential treatment effect or guiding clinical decisions.^17^ Evaluation requires a comparative effectiveness study design (e.g., randomized clinical trial, comparative cohort study).

- Differential Treatment Effect. Also known as differential treatment response. Different degrees or amounts of the construct being measured by the health measurement tool predict different magnitudes of treatment benefit. For example, those with a higher score may have a larger benefit that those with a lower score. Evaluation of differential treatment effect requires a comparative effectiveness study design, the gold standard for this is a randomized clinical trial, with a planned comparison by subgroups with interactions based on different levels of the measurement tool (e.g., comparing the effect of a treatment in those with high and low levels on the tool).
- Guide Clinical Decision. Also known as clinical decision rule. This occurs when a clinical decision, such as to initiate a treatment, is made based on a value or threshold on the health measurement tool. For example, a medication would be prescribed for a higher level on the tools, but not at a lower level. Evaluation of the effect of using a tool to guide a clinical decision requires a comparative effectiveness study design, the gold standard for this would be a randomized clinical trial, comparing use of the tool to guide the clinical decision vs standard practice (e.g., comparing the outcome of starting a medication based on a certain threshold on the tool vs starting the medication for all patients with a diagnosis).

*Pragmatic Factors*

The authors chose to use the term pragmatic factors to align with new recommendations for this metric detailed in the PAPERS framework.^10^ This framework used a Delphi process to strengthen the assessment of pragmatic measures of tools which have previously been described by words like usability, feasibility, ease, etc. These are the practical elements of implementing the tool in routine clinical settings. The PAPERS framework groups these into four categories: acceptability, ease of use, compatibility, and usefulness. Acceptability includes acceptability to stakeholders, relative advantages over other similar instruments, cost. Ease of use includes ease of completion, language accessibility, burden of training, burden of interpretation, instrument length. Compatibility includes appropriateness for stakeholders. Usefulness includes fit within organizational activities, and clinical decision-making guidance.

**Type of Investigation**

For each performance metric, we classified the type of investigation based on study design features. Type of investigation represents the domain in which a performance metric has been evaluated, and for some metrics the confidence/certainty in the findings of this evaluation. For each performance metric, types of investigation differ; these are described in detail here.

*Predictive Validity*

Based on recommendations for assessment of predictive validity, prognosis tools or prediction models, type of investigation was classified into several levels based on if frailty tool was assessed as a single prognostic factor, or as a prognostic model.^5,7,15–17^ Prognostic factors are a single value (could be created as a scale, sum, index, or other combination) that has an association with an outcome of interest, while prognostic models include frailty assessments within a statistic model that is used to estimate the risk of a future outcome. These levels reflect the certainty in conclusions that can be drawn from the study designs at each type of investigations.

Prognostic factor

- Exploratory. This reflected exploratory study designs, where conclusions of predictive validity are uncertain. Examples include no multivariable analysis; multivariable analysis, but using univariable screen or other statistical means for variable selection; multivariable analysis, but design used to filter multiple frailty tools, or variable selection not based on known prognostic factors
- Confirmatory. This reflected confirmatory study designs where conclusions are more certain. These studies are designed to evaluate the independent association of a specific prognostic factor in the context of other known prognostic factors. These are usually based on planned multivariable analyses including other known prognostic factors.
- Meta-Analysis. This is a study design that quantitatively pools the measures of predictive validity for a tool across many studies. This may increase confidence in results and conclusions.
- Incremental value. This is a higher-level study type designed to explicitly assess the value of a frailty assessment tool in addition to other known prognostic factors or models. This includes any direct assessment of model performance of a base/reference model compared to performance with addition of frailty tool (e.g., change in overall model fit, change in AUC/discrimination, change in calibration, change in overall predictive performance (e.g., R2, reclassification)).

Prognostic model

This includes two levels of prognostic model development and validation

- Development/internal validation. These studies aim to develop a novel statistical model to predict future outcomes using a development dataset. Internal validation uses the same dataset that a model was developed on by reassessing the model’s performance on a component of the dataset or using resampling techniques.
- External validation. These studies assess the performance of a known model using a dataset not used during model development. These studies may also update, adjust, or recalibrate the model. External validation increases the confidence in the assessment of a prognostic model’s performance.

We highlighted tools that have only been studied using exploratory study designs: exploratory for prognostic factors, and development/internal validation for prognostic models.

*Concurrent Criterion*

Based on recommendations for assessment of concurrent criterion or diagnostic test accuracy, tools were classified by purpose as either screening or formal diagnosis, and type of investigation was classified into three levels.^6,11–14^.

- Screening. Tests used for screening or triage are intended to identify a high proportion of those with a condition, but may falsely identify some without the condition. These are generally used on a diagnostic pathway, where a subsequent diagnostic test would be used to confirm the screening results.
- Formal Diagnosis. Tests used for formal diagnosis have more accurate performance in accurately classifying individuals with and without a condition.
- Agreement (Exploratory). The is an exploratory study design, where conclusions of diagnostic accuracy are uncertain. This includes any of the following statistical comparisons: agreement, concordance, correlation test (spearman, kappa, etc); association, univariable (no other variables were included in the statistical model); association, multivariable (other variables were included in the statistical model)
- Indirect comparison: This is where statistical measures for individual tools are listed and compared in magnitude, but there is no direct statistical comparison between these tools or to a gold standard. Conclusions about which test is better are uncertain. This includes measures of diagnostic test accuracy, but no statistical comparison between them: sensitivity/specificity; positive predictive value/negative predictive value; accuracy (statistical measure of proportion of all tests that give the correct result over all results); likelihood ratio; AUC (area under the curve)/ROC (receiver operator characteristic)/AUROC
- Direct comparison: use of real gold standard as reference standard (meaning CGA, Fried Phenotype, Rockwood FI in this scoping review), or a direct comparison of 2 or more index tests, and these comparisons use a direct statistical comparison of diagnostic accuracy measures (e.g., diagnostic OR). Conclusions about which test is better are more certain.

We highlighted tools that have only been studied using exploratory study designs: agreement (exploratory)

*Clinical Impact*

Type of investigation was classified by the type of clinical impact that was evaluated: differential treatment effect, or guide clinical decision.

*Pragmatic Factors*

Type of investigation was classified by which of the four categories of the PAPERS framework were evaluated: acceptability, ease of use, compatibility, and usefulness.

# Supplement Methods 4. Detailed eligibility criteria

**Eligibility Criteria**

The eligibility criteria were developed to adhere to scoping review methods recommending use of the population, concept, context, and study design framework for eligibility criteria. Further elaboration and explanation tables are available in **Supplement Methods 5** and detailed definitions for some elements are further elaborated in the data charting tables in **Supplement Methods 8**.

*Population*

We included studies involving older adults (65 years or older) undergoing major intracavitary, non-cardiac surgery.^20,21^ Noncavitary and cardiac surgeries were excluded due to distinct perioperative considerations and outcomes. We included studies that included only those aged 65 and older, with a mean or median age of 65 or older, with a majority of individuals 65 years and older by percentage, or where results are reported for a subgroup aged 65 and older.

*Concept*

We included studies reporting on the development or evaluation of a frailty assessment tool.^1–10^ Frailty is a state of vulnerability to stressors due to multisystem decline, operationalized through the cumulative deficit or phenotype models.^22–27^ Frailty assessment tools were considered as health measurement tools based on specific variables reflecting an established model of frailty.^28^ We excluded tools assessing a single domain or using laboratory/radiologic values alone. Studies that employed a different construct, only used frailty as an adjustment covariate, or lacked reporting on individual tool characteristics were excluded.

We included studies reporting on any performance metric of a frailty assessment tool used for diagnosis, prognosis, or clinical decisions. Performance metrics are defined in **Supplement Methods 3**.

*Context*

We included studies reporting on frailty assessment tools used in the clinical setting prior to surgery. We excluded tools not used in clinical practice, or not used preoperatively. This meant tools applied to administrative data or retrospectively on previously collected clinical data were excluded.

*Types of Evidence Sources*

We included randomized trials, cohort studies, diagnostic accuracy studies, measurement properties studies, prediction studies, usability studies, and systematic reviews. Qualitative studies were included if they evaluated the impact or clinical usability of frailty assessment tools. Studies without full-text publications were excluded.

# Supplement Methods 5. Eligibility criteria explanation and elaboration tables

| POPULATION – Does this study include older adults undergoing major intracavitary, noncardiac surgery? | |
| --- | --- |
| INCLUDE | EXCLUDE |
| Human participants  Older adults (age 65 years or older)   - Study includes only those aged 65 years or older - Mean or median age of 65 or older - Majority of individuals are 65 years or older - Results are reported for a subgroup of patients 65 years or older   Major intracavitary, noncardiac surgery   - Surgery conducted with either open, laparoscopic, or robotic technique within the abdomen, pelvis, or thorax - Studies with a mixed surgery types should include >80% intracavitary surgery, or present results for this subgroup - Elective or emergency surgery - Major surgery will be defined based on the definition in the study, or including surgery types that have an expected length of stay greater than 2 days. | - Nonhuman subjects - Not surgical patients - Patients younger than 65 years old - Cardiac surgery - Extremity, soft tissue, or breast surgery; orthopedic surgery, plastic surgery - Endovascular and extremity vascular surgery - Head and neck surgery - Neurosurgery or spine surgery; including anterior approach spine surgery - Trauma care (traumatic injury care and trauma surgery) - Transplant surgery - Bariatric surgery - Studies with mixed surgery types and less that 20% intracavitary surgery, or not reporting results for intracavitary surgery as a subgroup - Minor surgery or surgery types with an expected length of stay less than 2 days. - Mixed surgical and nonsurgical populations should be excluded; unless frailty tool assessment has been conducted and reported on the surgical subgroup separately.   NOTE* Studies that retrospectively derive their population from a large database of routinely collected data should be excluded unless they explicitly state that a clinically applied frailty tool was used. A common example of the is the “American College of Surgeons National Surgical Quality Improvement Program” or “NSQIP”. Studies that use this database should be excluded, unless they clearly use a clinical frailty tool, and use the NSQIP data only for outcomes assessments. This can be confusing because they talk of using the “mFI” or “Modified frailty Index”, but this is actually retrospectively computed based on data in the database, rather than an assessment done with the patient. If a study explicitly describes using the mFI prospectively, this could be included. |

| CONCEPT – Does this study assess a frailty assessment tool? | |
| --- | --- |
| INCLUDE | EXCLUDE |
| - Health measurement tools assessing frailty. Frailty is a state of vulnerability to stressors increasing the risk of adverse health outcomes due to multisystem decline in physiologic reserve and function. These tools typically are multicomponent tools developed to assess frailty based on an established model of frailty such as the phenotype model or the cumulative deficit model - Other terms that may be used for frailty are: vulnerability, physiologic age, functional age, accumulation of deficits | - Health measurement tools assessing constructs other than frailty (assessing other constructs such as disability, sarcopenia, comorbidity, function, chronologic age) - Tools assessing single domains such as physical performance alone, malnutrition alone, cognition alone, daily function alone, etc. - Single laboratory or radiologic values - Studies that base frailty assessment only on Comprehensive Geriatric Assessment or assessments that require an expert geriatrics team; that is, studies that do not assess frailty using an assessment tool that can be applied by a non-geriatrics expert. NOTE* that studies that compare another frailty assessment tool to Comprehensive Geriatric Assessment should be included. Also, sometimes "GA" or "geriatric assessment" is a term used, this can be included if conducted by non-geriatrician/geriatrics expert team - Studies using surgical risk scores that do not measure, assess, screen for, or diagnose frailty   NOTE* Disease-specific risk calculators (like RCRI, Revised Cardia Risk Index), the ASA (Anesthesia Society of America Physical Status assessment), and comorbidity indexes (like Charlson Comorbidity Index, CCI) should not be considered frailty assessment tools and should not be included. |

| CONCEPT – Does this study report on the performance metrics of a frailty assessment tool? | |
| --- | --- |
| INCLUDE | EXCLUDE |
| Studies that report on any of the following performance metrics of a frailty assessment tool:   - Validity (content/face validity, construct validity, structural validity, criterion validity) - Reliability (internal consistency, inter-rater reliability, test-retest reliability) - Measurement properties - Diagnostic test accuracy - Diagnostic performance - Sensitivity - Specificity - Positive predictive value/Negative predictive value - Accuracy - Likelihood ratio - Predictive performance - Prognostic performance - Overall performance (R^2^, Brier score) - Discrimination (Receiver operating curve, ROC, AUC, c statistic, concordance index, Integrated Discrimination Improvement) - Calibration (calibration plot, Hosmer-Lemeshow) - Reclassification (Net Reclassification Index) - Clinical usefulness, clinical impact, impact analysis, clinical decision rule, Difference in net benefit (NB), decision curve analysis (DCA) - Differential treatment effect - Decision curve analysis (DCA) - Utility - Prgamatic Factors. acceptability, ease of use, compatibility, and usefulness. Other terms to will be regroups into these four categories (e.g., useability, feasibility, acceptability, satisfaction, implementation, availability, language versions, practicality or training) - For systematic reviews, these will be only included if a relevant summary estimate of any of these outcomes is reported for individual frailty assessment tool(s). | - Studies that only include frailty as a variable in a multivariable model without reporting further characteristics of the frailty assessment on diagnosis, prognosis, measurement properties, or other outcomes - Studies developing novel prognostic or diagnostic models - Systematic reviews that report pooled outcomes across frailty assessment tools without outcomes for individual frailty assessment tools   Note. if frailty is used only in a multivariable model to adjust an analysis of another exposure (like surgical type, surgery vs no surgery, old vs young), this should be excluded. In contrast, if other variables are used to adjust for the effect of frailty, like what is the impact of frailty on mortality adjusted for age and comorbidity, this study could be included. |

| CONTEXT – Does this study assess a frailty assessment tool conducted clinically before surgery? | |
| --- | --- |
| INCLUDE | EXCLUDE |
| - Tool applied in the clinical setting either with patient-reported, caregiver-reported, or clinician-reported information using information derived in the clinical context including from the health record - Tool used before surgery | - Tools applied to administrative data (such as NSQIP), simulation models, computer models, laboratory setting, nonclinical research setting - Studies assessing frailty only after surgery |

| TYPE OF EVIDENCE SOURCE – Is this the right publication type? | |
| --- | --- |
| INCLUDE | EXCLUDE |
| Include all study designs that report of properties of a frailty assessment tool   - Randomized clinical trial (RCT) - Cohort Study (prospective or retrospective) - Prognosis study - Diagnostic accuracy study - Measurement properties study - Prediction study - Useability study (this may include feasibility, acceptability, satisfaction, implementation, or required training) - Systematic reviews of these types of studies - Qualitative studies only if evaluation of the impact or clinical useability of frailty assessment tools | - Narrative reviews - Clinical overviews - Literature reviews - Book chapters - Case reports - Editorials, letters to the editor, commentaries - Opinion pieces - Protocols - Studies without full text publications   NOTE* that narrative reviews, better termed clinical overviews, clinical summaries, or literature reviews are descriptive write-ups often styled like a book chapter, meaning they are not conducted as a study either using primary quantitative data or as a systematic review of the literature. Systematic reviews use explicit methods to systematically search the literature, extract data, and synthesis this (*note that sometimes narrative synthesis is used in a systematic review, but the study uses explicit methodology). Narrative reviews/Clinical overviews are often indicated when the abstract is simply a narrative block of text without any methods, objectives, or outcomes described. |

# Supplement Methods 6. Search Strategies

**Medline**

1	Frailty/di [Diagnosis]	1551

2	Frailty/ep [Epidemiology]	1475

3	Geriatric Assessment/	30072

4	frailty.ti.	8301

5	((frail or frailty or frailties) adj3 (diagnos* or assessment* or index or indices or score or scores or scale or scales or tool or tools or evaluat* or performance or instrument or instruments or analysis or analyses or questionnaire* or survey* or measure* or screen* or test or tests or testing)).tw,kf.	6846

6	geriatric assessment*.tw,kf.	4967

7	(prefrail or nonfrail).tw,kf.	902

8	physiologic age.tw,kf.	141

9	functional age.tw,kf.	173

10	"accumulation of deficit*".tw,kf.	111

11	(Balducci or Bern Scale or Columbia Scale or Essential Frailty Toolset or Frailty Phenotype or Fried or G8 or Geriatric-8 or Groningen Frailty Indicator or Risk Analysis Index or Rockwood or Triage Risk Screening Tool or Vulnerable Elderly Survey).tw,kf.	7808

12	1 or 2 or 3 or 4 or 5 or 6 or 7 or 8 or 9 or 10 or 11	45773

13	preoperative period/	8946

14	Preoperative Care/	64211

15	(preoperativ* or pre-operativ*).tw,kf.	350153

16	surg*.ti.	700454

17	"before surg*".kf,ab.	43153

18	13 or 14 or 15 or 16 or 17	1020021

19	"Predictive Value of Tests"/	215260

20	Prognosis/	550865

21	Risk Assessment/	289432

22	risk factors/	890626

23	"reproducibility of results"/	427661

24	(predict* or prognos* or risk or risks).tw,kf.	4277976

25	19 or 20 or 21 or 22 or 23 or 24	5135764

26	Postoperative Complications/	383329

27	Postoperative Period/	54483

28	(surg* or postoperativ* or post-operativ* or postsurg*).tw,kf.	2381606

29	26 or 27 or 28	2529224

30	25 and 29	679397

31	18 or 30	1449527

32	12 and 31	3376

33	limit 32 to yr="2005 -Current"	3043

34	limit 33 to "humans only (removes records about animals)"	3043

35	limit 34 to (case reports or comment or editorial or letter)	180

36	34 not 35	2863

**Cochrane Central**

1	Geriatric Assessment/	1564

2	frailty.ti.	656

3	((frail or frailty or frailties) adj3 (diagnos* or assessment* or index or indices or score or scores or scale or scales or tool or tools or evaluat* or performance or instrument or instruments or analysis or analyses or questionnaire* or survey* or measure* or screen* or test or tests or testing)).tw.	1112

4	geriatric assessment*.tw.	786

5	(prefrail or nonfrail).tw.	141

6	physiologic age.tw.	4

7	functional age.tw.	11

8	"accumulation of deficit*".tw.	9

9	(Balducci or Bern Scale or Columbia Scale or Essential Frailty Toolset or Frailty Phenotype or Fried or G8 or Geriatric-8 or Groningen Frailty Indicator or Risk Analysis Index or Rockwood or Triage Risk Screening Tool or Vulnerable Elderly Survey).tw.	758

10	1 or 2 or 3 or 4 or 5 or 6 or 7 or 8 or 9	3890

11	preoperative period/	348

12	Preoperative Care/	4362

13	(preoperativ* or pre-operativ*).tw.	44250

14	surg*.ti.	78976

15	"before surg*".ab.	208653

16	11 or 12 or 13 or 14 or 15	239445

17	"Predictive Value of Tests"/	7553

18	Prognosis/	14818

19	Risk Assessment/	9442

20	risk factors/	26240

21	"reproducibility of results"/	12120

22	(predict* or prognos* or risk or risks).tw.	348766

23	17 or 18 or 19 or 20 or 21 or 22	372425

24	Postoperative Complications/	18000

25	Postoperative Period/	4198

26	(surg* or postoperativ* or post-operativ* or postsurg*).tw.	262900

27	24 or 25 or 26	266761

28	23 and 27	56820

29	16 or 28	244795

30	10 and 29	373

31	limit 30 to yr="2005 -Current"	347

**Embase**

1	frailty/di [Diagnosis]	789

2	frailty/ep [Epidemiology]	612

3	clinical frailty scale/	396

4	geriatric assessment/	18442

5	frailty/ and clinical assessment tool/	121

6	((frail or frailty or frailties) adj3 (diagnos* or assessment* or index or indices or score or scores or scale or scales or tool or tools or evaluat* or performance or instrument or instruments or analysis or analyses or questionnaire* or survey* or measure* or screen* or test or tests or testing)).tw,kw.	11512

7	(prefrail or nonfrail).tw,kw.	1268

8	physiologic age.tw,kw.	213

9	functional age.tw,kw.	245

10	"accumulation of deficit*".tw,kw.	169

11	(Balducci or Bern Scale or Columbia Scale or Essential Frailty Toolset or Frailty Phenotype or Fried or G8 or Geriatric-8 or Groningen Frailty Indicator or Risk Analysis Index or Rockwood or Triage Risk Screening Tool or Vulnerable Elderly Survey).tw,kw.	11081

12	1 or 2 or 3 or 4 or 5 or 6 or 7 or 8 or 9 or 10 or 11	37655

13	preoperative evaluation/	143414

14	(preoperativ* or pre-operativ*).tw,kw.	506443

15	surg*.ti.	874204

16	"before surg*".kw,ab.	62552

17	13 or 14 or 15 or 16	1334128

18	exp Prediction/ and Forecasting/	1768

19	Risk Assessment/	635334

20	Risk Factors/	766633

21	reproducibility/	236539

22	(predict* or prognos* or risk or risks).tw,kw.	6013681

23	18 or 19 or 20 or 21 or 22	6541050

24	postoperative complication/	383145

25	postoperative period/	234949

26	(surg* or postoperativ* or post-operativ* or postsurg*).tw,kw.	3306241

27	24 or 25 or 26	3459451

28	23 and 27	931611

29	17 or 28	1933980

30	12 and 29	3964

31	limit 30 to yr="2005 -Current"	3792

32	limit 31 to "humans only (removes records about animals)"	3784

33	limit 32 to (editorial or letter)	61

34	32 not 33	3723

35	case report/	2767250

36	34 not 35	3638

37	limit 36 to embase	1717

**CINAHL Complete**

| S36 | S15 AND S34 | Limiters - Published Date: 20050101-20211231 | |
| --- | --- | --- | --- |
|  |  |  |  |
|  |  |  |  |
| S35 | S15 AND S34 | View Results (1,667) | |
|  |  |  |  |
|  |  |  |  |
| S34 | S21 OR S33 | View Results (482,528) | |
|  |  |  |  |
|  |  |  |  |
| S33 | S28 AND S32 | View Results (64,194) | |
|  |  |  |  |
|  |  |  |  |
| S32 | S29 OR S30 OR S31 | View Results (247,362) | |
|  |  |  |  |
|  |  |  |  |
| S31 | TI ( (surg* or postoperativ* or post-operativ* or postsurg*) ) OR TI ( (surg* or postoperativ* or post-operativ* or postsurg*) ) | View Results (187,083) | |
|  |  |  |  |
|  |  |  |  |
| S30 | (MH "Postoperative Period") | View Results (16,401) | |
|  |  |  |  |
|  |  |  |  |
| S29 | (MH "Postoperative Complications") | View Results (76,849) | |
|  |  |  |  |
|  |  |  |  |
| S28 | S22 OR S23 OR S24 OR S25 OR S26 OR S27 | View Results (1,321,666) | |
|  |  |  |  |
|  |  |  |  |
| S27 | TI ( (predict* or prognos* or risk or risks) ) OR AB ( (predict* or prognos* or risk or risks) ) | View Results (1,137,016) | |
|  |  |  |  |
|  |  |  |  |
| S26 | (MH "Reproducibility of Results") | View Results (67,629) | |
|  |  |  |  |
|  |  |  |  |
| S25 | (MH "Risk Factors") | View Results (190,294) | |
|  |  |  |  |
|  |  |  |  |
| S24 | (MH "Risk Assessment") | View Results (120,513) | |
|  |  |  |  |
|  |  |  |  |
| S23 | (MH "Prognosis") | View Results (89,528) | |
|  |  |  |  |
|  |  |  |  |
| S22 | (MH "Predictive Value of Tests") | View Results (54,240) | |
|  |  |  |  |
|  |  |  |  |
| S21 | S16 OR S17 OR S18 OR S19 OR S20 | View Results (471,897) | |
|  |  |  |  |
|  |  |  |  |
| S20 | AB "before surg*" | View Results (9,469) | |
|  |  |  |  |
|  |  |  |  |
| S19 | TI surg* OR AB surg* | View Results (442,560) | |
|  |  |  |  |
|  |  |  |  |
| S18 | TI ( (preoperativ* or "pre-operativ*") ) OR AB ( (preoperativ* or "pre-operativ*") ) | View Results (70,607) | |
|  |  |  |  |
|  |  |  |  |
| S17 | (MH "Preoperative Care") | View Results (17,914) | |
|  |  |  |  |
|  |  |  |  |
| S16 | (MH "Preoperative Period") | View Results (6,736) | |
|  |  |  |  |
|  |  |  |  |
| S15 | S1 OR S2 OR S3 OR S6 OR S7 OR S8 OR S9 OR S10 OR S11 OR S12 OR S13 OR S14 | View Results (25,090) | |
|  |  |  |  |
|  |  |  |  |
| S14 | TI ( (Balducci or Bern Scale or "Columbia Scale" or "Essential Frailty Toolset" or "Frailty Phenotype" or Fried or G8 or "Geriatric-8" or "Groningen Frailty Indicator" or "Risk Analysis Index" or Rockwood or "Triage Risk Screening Tool" or "Vulnerable Elderly Survey") ) OR AB ( (Balducci or Bern Scale or "Columbia Scale" or "Essential Frailty Toolset" or "Frailty Phenotype" or Fried or G8 or "Geriatric-8" or "Groningen Frailty Indicator" or "Risk Analysis Index" or Rockwood or "Triage Risk Scree ... | View Results (2,524) | |
|  |  |  |  |
|  |  |  |  |
| S13 | TI "accumulation of deficit*" OR AB "accumulation of deficit*" | View Results (30) | |
|  |  |  |  |
|  |  |  |  |
| S12 | TI "functional age" OR AB "functional age" | View Results (46) | |
|  |  |  |  |
|  |  |  |  |
| S11 | TI "physiologic age" OR AB "physiologic age" | View Results (31) | |
|  |  |  |  |
|  |  |  |  |
| S10 | TI ( (prefrail or nonfrail) ) OR AB ( (prefrail or nonfrail) ) | View Results (522) | |
|  |  |  |  |
|  |  |  |  |
| S9 | TI "geriatric assessment*" OR AB "geriatric assessment*" | View Results (2,098) | |
|  |  |  |  |
|  |  |  |  |
| S8 | TI ( ((frail or frailty or frailties) N3 (diagnos* or assessment* or index or indices or score or scores or scale or scales or tool or tools or evaluat* or performance or instrument or instruments or analysis or analyses or questionnaire* or survey* or measure* or screen* or test or tests or testing)) ) OR AB ( ((frail or frailty or frailties) N3 (diagnos* or assessment* or index or indices or score or scores or scale or scales or tool or tools or evaluat* or performance or instrument or instrum ... | View Results (4,025) | |
|  |  |  |  |
|  |  |  |  |
| S7 | TI frailty | View Results (5,195) | |
|  |  |  |  |
|  |  |  |  |
| S6 | S4 AND S5 | View Results (383) | |
|  |  |  |  |
|  |  |  |  |
| S5 | (MH "Clinical Assessment Tools") | View Results (175,066) | |
|  |  |  |  |
|  |  |  |  |
| S4 | (MH "Frailty Syndrome") | View Results (2,967) | |
|  |  |  |  |
|  |  |  |  |
| S3 | (MH "Geriatric Assessment+") | View Results (17,608) | |
|  |  |  |  |
|  |  |  |  |
| S2 | (MH "Frailty Syndrome/EP") | View Results (203) | |
|  |  |  |  |
|  |  |  |  |
| S1 | (MH "Frailty Syndrome/DI") | View Results (523) | |

# Supplement Methods 7. Details on selection of sources of evidence and data charting

**Selection of Sources of Evidence**

We used the Covidence web-based platform for systematic review management.^29^ Two levels of screening were conducted: Level One screened titles and abstracts, and Level Two screened full texts. Two independent reviewers assessed citations at both stages using a piloted selection form and accompanying explanation and elaboration documents based on the eligibility criteria (**Supplement Methods 4 and 5**). Citations selected in Level One screening by at least one reviewer proceeded to Level Two screening. Training and calibration exercises were conducted for consistency. In Level One screening, the acceptance criterion for agreement was set at no more than 20% of studies included by the review leads (TRC and JFD) being excluded by one or more reviewers. In Level Two screening, a review lead served as the second reviewer for each citation to ensure rigor. Exclusion reasons during full text screening were recorded. Search and selection results were reported using a PRISMA flow diagram. Authors were contacted via email for 5 studies for additional information needed to determine eligibility. Disagreements were resolved through discussion, involving a third reviewer if necessary.

**Data Charting**

Two independent reviewers charted data from each eligible record, prioritizing the record with the most complete or recent information in cases of multiple records for the same study. Electronic data charting forms were developed and pilot-tested using Covidence, accompanied by a detailed manual with additional details and definitions of the data items. Calibration exercises were conducted, discrepancies were discussed, and form refinements were made as needed. A review lead served as the second reviewer for each citation to ensure accuracy. Disagreements were resolved through discussion or involving a third reviewer when needed. Contacting study authors for additional information was not required.

Data items encompassed study and population characteristics, frailty assessment tool characteristics, performance metrics assessed, and comparisons between tools. If a record reported on more than one frailty assessment tool, we charted relevant data for each tool presented. Extensive detail of definitions and assumptions are provided in the explanation and elaboration tables in **Supplement Methods 8**. Detailed definitions of measurement properties, clinical impact, and pragmatic factors are provided in these tables. Criterion validity is the degree to which a tool reflects a “gold standard”.^2,3^ When tools are used for diagnosis, criterion validity is assessed by the concurrent criterion, which is the degree by which the tool matches the results of a gold standard test at the same timepoint. Data charting of measurement properties of tools used for diagnosis included a specific focus on concurrent criterion and was elaborated based on terminology used for diagnostic test accuracy (**Supplement Methods 8**).^5,18^ When tools are used for prognosis, criterion validity is assessed by predictive validity, which is the accuracy with which the tool predicts a future outcome. As such, we used the term predictive validity when discussing tools used for prognosis or predicting a future outcome. Data charting of measurement properties of tools used for prognosis included a specific focus on predictive validity and was elaborated based on terminology used for prognosis research (**Supplement Methods 8**).^5,7^ When tools are assessed for clinical impact, this is usually conducted using a prospective trial design including a control group (best assessed in an RCT, but could be assessed using other designs such a prospective comparative cohort study or before-and-after design).^7^ Clinical impact can include an assessment of differential treatment effect: those with and without frailty have a different magnitude of benefit from a specific treatment. Tools can also be used as a clinical decision rule to guide a clinical decision for or against a treatment or treatment pathway; evaluations of tools used for clinical decisions would assess patient outcomes in a trial assessing the tool clinically applied to make a treatment decision. Finally, due to varying language used to indicted usability, feasibility, acceptability; we adopted the PAPERS framework, which provided terminology and definitions based on a Delphi process, to chart pragmatic factors.^10^ This framework which groups pragmatic factors into four categories: acceptability, ease of use, compatibility, and usefulness. Acceptability includes acceptability to stakeholders, relative advantages over other similar instruments, cost. Ease of use includes ease of completion, language accessibility, burden of training, burden of interpretation, instrument length. Compatibility includes appropriateness for stakeholders. Usefulness includes fit within organizational activities, clinical decision-making guidance.

# Supplement Methods 8. Data charting form explanation and elaboration tables

| **Study and Population Items** | |
| --- | --- |
| This section includes items about the study and bibliometrics, the study population, study type, and setting. | |
| Publication type | - Randomized clinical trial (RCT) (original study or post-hoc analysis) - Cohort Study (prospective or retrospective) - Prognosis study - Diagnostic accuracy study - Measurement properties study - Prediction study - Useability study (this may include feasibility, acceptability, satisfaction, implementation, or training) - Systematic review - Qualitative study |
| Study site | Single centre or multicentre |
| Country of origin | List country or multinational |
| Study size | - Number of participants meeting criteria for older adult - If number of older adults not reported in a study including younger adults as well, list total number of participants |
| Age of study participants | Preferentially list in this order   - Age cutoff if 65 years or older - Mean or median age if no age cutoff of 65 or older - Percentage older adults (%) if no age cutoff or mean/median and majority of individuals are 65 years or older |
| Sex and gender of participants | List number or proportion (%) of each sex and gender reported. |
| Proportion rated as frail | List proportion (%) or participants rated as frail in the study |
| Follow-up | Mean/median  (not applicable for diagnosis studies) |
| Setting | Emergency, Benign, Oncology, mixed |
| Surgery Type | General surgery, colorectal, gynecology, etc, mixed |
| Type of tool assessment | List if this is an initial development study for the tool in the surgical population, a subsequent evaluation study of the properties of a tool previously developed, or a study that directly compares two or more assessment tools. Note that comparisons should be between two frailty assessment tools, not against a gold standard such as Comprehensive Geriatric Assessment (if this is the case, list as either development or validation/evaluation as appropriate).   - Development - Validation/Evaluation - Comparison - Pragmatic factors (list only if this is the only purpose) - Mixed (used for more than one purpose in this study) |

| **Frailty Assessment Tool Characteristics** | |
| --- | --- |
| This section includes items about the frailty assessment tool including its name, details of the tools, and the purpose of the tool.^22,23,27^ | |
| Name | Record the name of the frailty assessment tool including abbreviation |
| Conceptual framework | List the theoretical or operational definition of frailty that supports to the frailty assessment tool.   - Cumulative deficits model (see note below) - Phenotype model - Multidimensional - None   Frailty is a state of vulnerability to stressors due to multisystem decline, operationalized through the cumulative deficit or phenotype models. Content validity refers to the degree to which the content of a measurement tool reflects the construct being measured.^3^ Frailty assessment tools will be considered to reflect a conceptual framework of frailty if they are based on an accepted theoretical or operational definition of frailty. If the operational definition used to develop the frailty assessment tool is not reported explicitly in the study report, this will be sought from an original development study for the frailty assessment tool. If this does not exist, the study team will assign an operational definition if this can be discerned by comparison with other tools. This will be reported. If this is not possible, this will also be reported. If the tool assesses multiple geriatric domains, but were not explicitly developed based on cumulative deficits or phenotype models, this can be listed as “multidimensional”.  NOTE* There are standard procedures for creating a cumulative deficits frailty index.^30^ This includes selecting at least 30-40 variables that represent deficits associated with health status, more prevalent with increasing age but not be too common at younger ages, and cover a range of systems or domains. Indexes composed of around 10 or fewer variables are unstable. When these elements are not met, the frailty assessment tool will not be considered to align with the cumulative deficits model. |
| Tool type | Frailty assessment tools can be of several types based on the type of data used as input for the tool.^31^   - Questionnaire: based on a list of questions. These can be answered by a clinician, the patient, or a proxy. - Performance: based on a physical performance test - Judgement: uses an overall assessment by a clinician based on input data - Routine data: based on routine health data collected for other purposes, usually housed in administrative datasets. These are not clinically applied frailty tools, and have been excluded from this review - Mixed: includes more than one type |
| Geriatric Domains | List which geriatrics domains are included in the frailty assessment tool.^32,33^   - Functional independence - Physical performance (using a physical performance test) - Falls & mobility - Comorbidity - Nutrition - Polypharmacy - Social support - Cognition - Mood - Other |
| Scale | List the type of scale of the frailty assessment tool output   - Categorical - Ordinal - Continuous   Categorical scales have distinct groups, but are not in a particular order; this could include dichotomous scale with only two groups (e.g., frail, not frail). Ordinal scales have distinct groups, but the order of the groups is defined (e.g., frail, pre-frail, fit). Continuous scales do not have distinct groups, are ordered, and the difference between intervals is consistent (e.g., a scale from 0 to 100) |
| Scoring Method | How is the tool scored   - Sum - Mean - Weighted - Threshold - Index |
| Language Versions | List the languages that the tool is available in |
| Purpose of tool | List the purpose of the tool in this study   - Diagnosis - Prognosis - Estimating treatment effect - Clinical decision rule   Diagnosis: the tool is used for identification of frailty at a single timepoint (preoperatively in this case). This may be either a screening test to identify patients that should have further diagnostic assessment to confirm the diagnosis of frailty, or a more definitive diagnostic test  Prognosis: the tool is used for estimating the likelihood of a future outcome.  Estimating treatment effect: the tool is used to estimate the benefit from a given treatment  Clinical decision rule: tool results used to direct a treatment decision, and studies of this kind evaluate outcomes based on treatment decision guided by the tool/clinical decision rule |
| Tool development | Was the tool developed in a surgical population?   - Surgical - Non-surgical - Unknown   If this is not the initial development study, this may be described in the introduction of methods sections. If not described, mark as unknown |
| Access | List where the tool can be accessed (e.g., publication or website) |
| Training required | Indicate if training is needed to employ the tool |
| Administration time | List time to administer tool |
| Mode of administration | List if tool is administered by the patient/caregiver, clinician, or either |
| Special equipment | List any special equipment needed to administer the tool |
| Licence needed | List if a license is needed to administer the tool |

| **Measurement Properties of Frailty Assessment Tools** | |
| --- | --- |
| This section includes items on measurement properties that can be assessed across all measurement tools in medicine. | |
| Measurement properties of health measurement tools | List any other measurement properties that are reported about the frailty assessment tool. Health measurement tools can be assessed across multiple properties to document their performance including measures of validity and reliability. Examples include  Validity   - Construct validity: this is an evaluation of the internal structure of a measurement tool and includes, cross-cultural validity, and convergent or divergent (discriminant) validity, or extreme-groups/known-groups (discriminative) validity. E.g., evaluating a similar but difference construct (physical performance, sarcopenia, disability, multimorbidity). - Criterion validity (assessed separately in tables below for diagnosis and prognosis)   Reliability   - Internal consistency - Inter-rater reliability - Test-retest reliability - Measurement error   Responsiveness (this will not be relevant to tools used for a single preoperative assessment)  Structural validity: an assessment of the adequacy of the dimensionality of the tool for measuring the construct (e.g., frailty) and may involve factor analysis, or item response theory/Rasch analysis.  Internal consistency: an assessment of the interrelatedness among scale items, and may be assessed by Cronbach’s alpha.  Measurement invariance: also called cross-cultural validity, is an assessment of the degree to which the performance of a new population reflects the initial measurement performance. This can be assess by differential item functioning (DIF), or multigroup confirmatory factor analysis (MGCFA)  Convergent or discriminative validity: this involves hypothesis testing about how the tool compares to another reference standard of good quality, or how the tools differs between groups known to be different.  Criterion validity: an assessment of the degree the tool reflects a reference or criterion standard. The elements of this are described in more detail below separately for diagnosis (concurrent criterion), and prognosis (predictive validity)  Internal consistency: an assessment of the interrelatedness among scale items, and may be assessed by Cronbach’s alpha.  Reliability: an assessment of degree to which a the measurement is the same for patients who have not changed by different raters (inter-rater), and over time (test-retest). This may be assessed by interclass correlation (ICC), or kappa.  Responsiveness: generally considered an assessment of the ability to detect important changes; multiple approaches to assessing responsiveness exist.  Interpretability: ability to assign qualitative meaning with clinical or commonly understood connotations for a score or change in score. |

| **Measurement Properties of Tools Used for Diagnosis** | |
| --- | --- |
| For tools that are used for diagnosis, the items in this section should be charted. These items reflect important properties of diagnostic tools. | |
| Type of Diagnosis Study | - Screening or triage - Formal diagnosis |
| Reference standard | List the measure that the frailty assessment tool evaluated against |
| Diagnostic accuracy measures (criterion validity/concurrent criterion)  *use if reports only association or correlation reported | How well does the tool identify a current health condition (frailty)?  Some studies simply report on a tool-health condition association or correlation by comparing to another reference tools. This is the weakest measure of concurrent criterion validity.  Was the outcome reported for the diagnostic performance an association or correlation only?   - Yes, univariable association (no other variables were included in the statistical model) - Yes, multivariable association (other variables were included in the statistical model) - Yes, correlation (e.g., Spearman’s) - No, other measurement properties are reported   Studies reporting this will report only an effect estimate such as an odds ratio (OR), or a correlation coefficient. |
| Diagnostic accuracy measures (criterion validity/concurrent criterion)  *use if reports more than association or correlation reported | How well does the tool identify a current health condition (frailty)?  If the study reports more than an association or correlation alone, list these measurement properties here. Examples include   - Sensitivity - Specificity - Positive predictive value/Negative predictive value - Accuracy - Likelihood ratio |

| **Measurement Properties of Tools Used for Prognosis** | |
| --- | --- |
| For tools that are used for prognosis, the items in this section should be charted. These items reflect important properties of prognostic tools. | |
| Multivariable analysis? | Is this tool reported as a single factor for prognosis, or added to other prognosis factors? If added to other factors, is this a previously known reference model.   - Single factor - Added to other prognosis factors - Added to reference model |
| Other prognosis factors | If tool is added to other factors, list them here  If added to a reference model, list this here |
| Predicted outcome | Which outcome(s) has the frailty assessment tool reported to prognosticate/predict?  Note: prognosis is the prediction of a future outcome. |
| Purpose of prognosis study | List the type of prognosis study. Prognosis studies of single factors that provide just effect estimates can be exploratory, confirmatory, or replication studies. Prognosis studies of a prognostic model (which are statistical models that provide estimates (proportion or percentage) for the likelihood of a given outcome), can be development, internal validation, external validation, or impact analyses. Other studies can assess a single factor against other known prognosis factors or a known reference model for incremental value.^5,8,15–17^   - Exploratory - Confirmatory - Development - Internal Validation - External Validation - Impact analysis - Incremental value   If more than one type of study is reported in a single evidence source, list this as the highest type as per the order listed above.  Exploratory: these studies aim to identify potential prognostic factors out of a group of collected variables. These studies usually do not focus on one specific factor and its prognostic performance. Studies of this kind may include frailty along with many other candidate variables, rather than focus on the performance of frailty specifically.  Confirmatory: these studies are designed to evaluate the independent association of a specific prognostic factor in the context of other known prognostic factors. These are usually based on planned multivariable analyses including other known prognostic factors.  Development & Internal Validation: these studies aim to develop a novel statistical model to predict future outcomes using a development dataset. Internal validation uses the same dataset that a model was developed on by reassessing the models performance on a component of the dataset or using resampling techniques.  External validation: these studies assess the performance of a known model using a dataset not used during model development. These studies may also update, adjust, or recalibrate the model.  Incremental value: these studies are a special type of confirmatory study that are designed to evaluate the incremental value of a specific prognostic factor by adding to a known reference model (prognostic model), or to a model generated from routinely available prognostic factors. |
| Prognostic or predictive accuracy (criterion validity/predictive validity)  *use if reports association only | How well does the tool predict a future outcome?  Some studies simply report on a tool-outcome association. This is the weakest measure of criterion/predictive validity.  Was the outcome reported for the prognostic performance reported as an association only?   - Yes, univariable association (no other variables were included in the statistical model) - Yes, multivariable association (other variables were included in the statistical model) - No, other measurement properties are reported   Studies reporting this will report only an effect estimate such as an odds ratio (OR). |
| Prognostic or predictive accuracy (criterion validity/predictive validity)  *Use if reports more than association only | How well does the tool predict a future outcome?  If the study reports more than an association or correlation alone with prognosis/prediction of an outcome, list these measurement properties here. Examples include   - Sensitivity, specificity, PPV, NPV, likelihood ratios - Discrimination (Receiver operating curve, ROC, AUC, c statistic, concordance index) - Calibration (calibration plot, Hosmer-Lemeshow) - Overall performance (R^2^, Brier score) - Reclassification: Net Reclassification Index (NRI), integrated discrimination improvement (IDI) - Clinical usefulness, clinical impact, impact analysis, clinical decision rule, Difference in net benefit (NB), decision curve analysis (DCA) - Decision curve analysis (DCA) |

| **Tools Assessed for Clinical Impact** | |
| --- | --- |
| Type | What type of clinical impact is being assessed   - Differential treatment effect (those with and without frailty have a different magnitude of benefit from a specific treatment) - Guide a clinical decision for or against a treatment or treatment pathway |

| **Pragmatic Factors** | |
| --- | --- |
| Factor | List all pragmatic factors reported in the study.  These are based on the PAPERS framework which groups these into four categories: acceptability, ease of use, compatibility, and usefulness.^10^ Acceptability includes acceptability to stakeholders, relative advantages over other similar instruments, cost. Ease of use includes ease of completion, language accessibility, burden of training, burden of interpretation, instrument length. Compatibility includes appropriateness for stakeholders. Usefulness includes fit within organizational activities, clinical decision-making guidance.  The definitions across studies may differ. List any additional metrics encountered even if not listed here. Other terms used ay include: feasibility, completion time, required equipment, satisfaction, implementation, practicality or training. |

| **Items for Studies Comparing Two or More Tools** | |
| --- | --- |
| Tools included | List the frailty assessment tools |
| Method for comparison | List the method used for comparing the tools  List the outcomes that were used to compare the tools |

# Supplement Methods 9. Details on synthesis of results for type of investigation and comparisons

**Synthesis of Results**

*Comparisons*

We summarized the number of studies comparing two or more tools, as well as specific details regarding the comparison: the performance metric being compared, if a reference standard was used versus simply comparing screening tools, and if the comparison was a direct of indirect comparison. We classified comparisons as an indirect comparison if a statistic or effect measure was reported for individual tools without a direct statistical comparison of the performance between the tools. This would be a presentation of the statistic or effect measrure (e.g., OR, AUC, sensitivity, specificity) for each tool presenting the magnitude of the effect measures across tools without a statistical comparison between them. In contrast, direct comparisons should include a statistical test evaluating the superiority or non-inferiority of the performance metric between two or more individual tools. These more direct comparisons could include statistical tests such as relative sensitivity, relative diagnostic odds ratio (rDOR), McNemar’s test of differences in sensitivity or specificity, comparison of discrimination, calibration, brier score, incremental value, net reclassification improvement.^5,7,18,19^ Comparison to a reference standard was considered present if a frailty assessment tool was compared to a standard diagnostic process for frailty, documented explicitly to be assessed by a healthcare practitioner or team with geriatric expertise. Comparison to a reference standard is in contrast to comparison to another index tool.

# Supplementary Figures and Tables

# Supplementary Figure S1. PRISMA 2020 Flow Diagram for Record Identification

# Supplementary Table S1. Characteristic of Supporting Studies

|  | **N (%), or**  **Median (IQR)** |
| --- | --- |
| **Studies** | 160 (100%) |
| **Tools** | 35 (100%) |
| **Study Size** | 187 (114-294) |
| **Female (%)** | 47% (39 - 53%) |
| NR | 21 (17%) |
| **Frail (%)** | 32% (23 - 48%) |
| Multiple reported | 9 (6%) |
| NR | 25 (16%) |
| **Study Site** |  |
| Single centre | 118 (74%) |
| Multicentre | 33 (21%) |
| **Study Design** | |
| RCT | 0 (0%) |
| Prospective Cohort | 109 (68%) |
| Retrospective Cohort | 38 (24%) |
| Qualitative Study | 1 (1%) |
| **Country** |  |
| Multinational | 5 (3%) |
| Australia | 1 (1%) |
| Belgium | 2 (1%) |
| Canada | 3 (3%) |
| China | 12 (8%) |
| Finland | 3 (2%) |
| Germany | 2 (1%) |
| Italy | 13 (8%) |
| Japan | 29 (18%) |
| Mexico | 1 (1%) |
| Netherlands | 9 (6%) |
| New Zealand | 1 (1%) |
| Norway | 1 (1%) |
| Peru | 1 (1%) |
| Poland | 6 (4%) |
| Singapore | 2 (1%) |
| South Korea | 3 (2%) |
| Spain | 7 (4%) |
| Taiwan | 2 (1%) |
| Turkey | 2 (1%) |
| United Kingdom | 8 (5%) |
| United States | 32 (20%) |
| Vietnam | 1 (1%) |

# Supplementary Table S2. Frailty assessment tools and investigating studies

| **Abbreviated Comprehensive Geriatric Assessment (aCGA)** | | | **2 studies** |
| --- | --- | --- | --- |
|  | Kenig 2015b | Screening for frailty among older patients with cancer that qualify for abdominal surgery |  |
|  | Kenig 2020 | Usefulness of eight screening tools for predicting frailty and postoperative short- and long-term outcomes among older patients with cancer who qualify for abdominal surgery |  |
| **Balducci Frailty Criteria** | |  | **3 Studies** |
|  | Kenig 2015a | Six screening instruments for frailty in older patients qualified for emergency abdominal surgery |  |
|  | Kenig 2015b | Screening for frailty among older patients with cancer that qualify for abdominal surgery |  |
|  | Kenig 2020 | Usefulness of eight screening tools for predicting frailty and postoperative short- and long-term outcomes among older patients with cancer who qualify for abdominal surgery |  |
| **Clinical Frailty Scale (CFS)** | | | **41 studies** |
|  | Arteaga 2021 | Impact of frailty in surgical emergencies. A comparison of four frailty scales |  |
|  | Artiles-Armas 2019 | The Comprehensive Complication Index is Related to Frailty in Elderly Surgical Patients |  |
|  | Artiles-Armas 2021 | Impact of frailty on 5-year survival in patients older than 70 years undergoing colorectal surgery for cancer |  |
|  | Carter 2020 | Association between preadmission frailty and care level at discharge in older adults undergoing emergency laparotomy |  |
|  | Cooper 2021 | Polypharmacy and frailty in older adults evaluated at a multidisciplinary geriatric-thoracic clinic prior to surgery |  |
|  | Czajka 2023 | Is Frailty a Good Predictor of Postoperative Complications in Elective Abdominal Surgery?-A Single-Center, Prospective, Observational Study |  |
|  | Darvall 2020 | Accuracy of the Clinical Frailty Scale for perioperative frailty screening: a prospective observational study |  |
|  | Eamer 2018 | Analysis of postdischarge costs following emergent general surgery in elderly patients |  |
|  | Fagard 2028 | Frailty screening in older patients undergoing elective colorectal surgery: Comparative study of seven screening instruments |  |
|  | Gilan 2025 | Frailty as a predictor of postoperative morbidity and mortality in older patients undergoing emergency laparotomy: a prospective cohort study in Yemen |  |
|  | Goh 2024 | Assessing the impact of frailty in elderly patients undergoing emergency laparotomies in Singapore |  |
|  | Hu 2025 | Frailty and Intrinsic Capacity as Predictors of Recovery in Older Patients Surgically Treated for Cancer: A Longitudinal Study |  |
|  | Isand 2025 | Impact of preoperative frailty on outcomes in patients subjected to emergency laparotomy: a prospective study |  |
|  | Li 2018 | Impact of frailty on outcomes afer discharge in older surgical patients: A prospective cohort study |  |
|  | Mak 2024 | Patients' frailty and co-morbidities do not affect short-term mortality following emergency colorectal cancer surgery |  |
|  | Mima 2020 | Frailty is an independent risk factor for recurrence and mortality following curative resection of stage I-III colorectal cancer |  |
|  | Morito 2023 | Frailty Assessed by the Clinical Frailty Scale is Associated with Prognosis After Esophagectomy |  |
|  | Ng 2025 | Changes in frailty status and discharge destination post emergency laparotomy |  |
|  | Niemelainen 2021 | The Clinical Frailty Scale is a useful tool for predicting postoperative complications following elective colon cancer surgery at the age of 80 years and above: A prospective, multicentre observational study |  |
|  | Niemelainen 2022 | One-year functional outcomes of patients aged 80 years or more undergoing colonic cancer surgery: prospective, multicentre observational study |  |
|  | Niemelainen 2025 | Clinical Frailty Scale predicts overall survival after colon cancer surgery in people aged 80 years and older: A prospective multicentre observational study |  |
|  | Normann 2025 | Frailty Is an Independent Marker of Post-Operative Mortality Following Colorectal Cancer Resection Surgery in Older Adults |  |
|  | O'Mahony 2021 | Cardiopulmonary Exercise Testing Versus Frailty, Measured by the Clinical Frailty Score, in Predicting Morbidity in Patients Undergoing Major Abdominal Cancer Surgery |  |
|  | Okabe 2019a | Frailty Predicts Severe Postoperative Complication after Elective Hepatic Resection |  |
|  | Okabe 2019b | Frailty predicts severe postoperative complications after elective colorectal surgery |  |
|  | Okabe 2021 | Frailty Predicts Severe Postoperative Complications After Elective Minimally Invasive Surgery in Patients with Colorectal Cancer |  |
|  | Parini 2024 | Comparison of frailty indexes as predictors of clinical outcomes after major thoracic surgery |  |
|  | Parmar 2021 | Frailty in Older Patients Undergoing Emergency Laparotomy: Results From the UK Observational Emergency Laparotomy and Frailty (ELF) Study |  |
|  | Price 2025 | Estimating the effect of frailty on longer-term survival following emergency laparotomy: an observational study using National Emergency Laparotomy Audit data |  |
|  | Rosa 2023 | Frailty assessment as independent prognostic factor for patients >=65 years undergoing urgent cholecystectomy for acute cholecystitis |  |
|  | SanchezArteaga 2022 | Long-term influence of frailty in elderly patients after surgical emergencies |  |
|  | Stamatakos 2022 | Outcomes of radical cystectomy in pT4 bladder cancer frail patients: Alpha high-volume single center study |  |
|  | Sun 2020 | Clinical Frailty Scale in Predicting Postoperative Outcomes in Older Patients Undergoing Curative Surgery for Urologic Malignancies: A Prospective Observational Cohort Study |  |
|  | Tanaka 2019 | Impact of Frailty on Postoperative Outcomes for Laparoscopic Gastrectomy in Patients Older than 80 Years |  |
|  | Vilches-Moraga 2020 | Emergency laparotomy in the older patient: factors predictive of 12-month mortality-Salford-POPS-GS. An observational study |  |
|  | Xing 2023 | Preoperative prediction of adverse outcome after elective gastrointestinal surgery in older patients: three leading frailty instruments and the American Society of Anesthesiologists physical status |  |
|  | Yamada 2021a | Significance of Frailty in Prognosis After Hepatectomy for Elderly Patients with Hepatocellular Carcinoma |  |
|  | Yamada 2021b | Significance of frailty in prognosis after surgery in patients with pancreatic ductal adenocarcinoma |  |
|  | Yin 2021 | Comparison of three frailty measures for 90-day outcomes of elderly patients undergoing elective abdominal surgery |  |
|  | Yin 2022 | Which Frailty Evaluation Method Can Better Improve the Predictive Ability of the SASA for Postoperative Complications of Patients Undergoing Elective Abdominal Surgery? |  |
|  | Youssef 2022 | Frailty is Associated With Poor Outcomes Following Emergency Laparotomy: What's Next? |  |
| **Edmonton Frail Scale (EFS)** | | | **15 studies** |
|  | Celik 2024 | Investigation of the Effect of Frailty Levels of Elderly Patients on their Recovery Status after General Surgery |  |
|  | Chang 2024 | Improving Surgical Care and Outcomes in Older Cancer Patients Through Implementation of a Presurgical Toolkit (OPTI-Surg) - Final Results of a Phase III Cluster Randomized Trial (Alliance A231601CD) |  |
|  | Czajka 2023 | Is Frailty a Good Predictor of Postoperative Complications in Elective Abdominal Surgery?-A Single-Center, Prospective, Observational Study |  |
|  | Darvall 2020 | Accuracy of the Clinical Frailty Scale for perioperative frailty screening: a prospective observational study |  |
|  | Ehrlich 2022 | Early Outcomes following Implementation of a Multispecialty Geriatric Surgery Pathway |  |
|  | Fagard 2027 | Frailty screening in older patients undergoing elective colorectal surgery: Comparative study of seven screening instruments |  |
|  | He 2020 | Assessment of predictive validity and feasibility of Edmonton Frail Scale in identifying postoperative complications among elderly patients: a prospective observational study |  |
|  | Moro 2017 | Frailty and elderly in urology: Is there an impact on post-operative complications? |  |
|  | Nishijima 2021 | Preoperative frailty assessment with the Robinson Frailty Score, Edmonton Frail Scale, and G8 and adverse postoperative outcomes in older surgical patients with cancer |  |
|  | Owodunni 2021 | Preoperative Frailty Assessment, Operative Severity Score, and Early Postoperative Loss of Independence in Surgical Patients Age 65 Years or Older |  |
|  | Owodunni 2024 | Validation of the Self-Reported Edmonton Frail Scale - Acute Care in Patients >= 65 Years Undergoing Surgery |  |
|  | Parini 2024 | Comparison of frailty indexes as predictors of clinical outcomes after major thoracic surgery |  |
|  | Richards 2021 | Pre-operative frailty is predictive of adverse post-operative outcomes in colorectal cancer patients |  |
|  | SengulAycicek 2021 | Impact of frailty and ultrasonography-based sarcopenia on the development of postoperative complications in gastrointestinal cancer patients |  |
|  | You 2028 | Application value of different frailty assessment tools in older patients undergoing major abdominal surgery |  |
| **Emergency General Surgery Frailty Index (EGSFI)** | | | **2 studies** |
|  | Khan 2019 | Failure to Rescue After Emergency General Surgery in Geriatric Patients: Does Frailty Matter? |  |
|  | OroujiJokar 2016 | Emergency general surgery specific frailty index: A validation study |  |
| **Essential Frailty Toolset (EFT)** | | | **1 study** |
|  | Zhu 2020 & 2021  (two reports using same study population) | Frailty as a predictor of increased one-year mortality after elective non-cardiac surgery: A prospective cohort study |  |
| **FRAIL scale** | |  | **15 studies** |
|  | Arteaga 2021 | Impact of frailty in surgical emergencies. A comparison of four frailty scales |  |
|  | Chen 2022 | Frailty is an independent risk factor for postoperative pulmonary complications in elderly patients undergoing video-assisted thoracoscopic pulmonary resections |  |
|  | Fagard 2029 | Frailty screening in older patients undergoing elective colorectal surgery: Comparative study of seven screening instruments |  |
|  | Mahanna-Gabrielli 2020 | Frailty Is Associated With Postoperative Delirium But Not With Postoperative Cognitive Decline in Older Noncardiac Surgery Patients |  |
|  | SanchezAcedo 2020 | Prospective Study of Factors Associated With Postoperative Delirium After Urgent Abdominal Surgery |  |
|  | SanchezArteaga 2022 | Long-term influence of frailty in elderly patients after surgical emergencies |  |
|  | Sokas 2020 | Association Between Patient-Reported Frailty and Non-Home Discharge Among Older Adults Undergoing Surgery |  |
|  | Ushigome 2025 | Frailty Recovery Following Minimally Invasive Surgery: An Emerging Perspective on Surgical Benefits in Elderly Colorectal Cancer Patients |  |
|  | Wilson 2021 | Implementation of a Frailty Assessment and Targeted Care Interventions and Its Association with Reduced Postoperative Complications in Elderly Surgical Patients |  |
|  | Xing 2023 | Preoperative prediction of adverse outcome after elective gastrointestinal surgery in older patients: three leading frailty instruments and the American Society of Anesthesiologists physical status |  |
|  | Xu 2023 | Efficacy of a modified FRAIL scale in predicting the peri-operative outcome of hepatectomy in older adults (aged >= 75 years): a model development study |  |
|  | Yin 2021 | Comparison of three frailty measures for 90-day outcomes of elderly patients undergoing elective abdominal surgery |  |
|  | Yin 2022 | Which Frailty Evaluation Method Can Better Improve the Predictive Ability of the SASA for Postoperative Complications of Patients Undergoing Elective Abdominal Surgery? |  |
|  | You 2026 | Application value of different frailty assessment tools in older patients undergoing major abdominal surgery |  |
|  | Yu 2025 | Association of Preoperative Frailty and Post-Induction Hypotension in Elderly Patients Undergoing Major Non-Cardiac Surgery: A Prospective Observational Cohort Study |  |
| **Frailty and Emergency Surgery in the Elderly (FRAILESEL)** | | | **1 study** |
|  | Costa 2021 | The Emergency Surgery Frailty Index (EmSFI): development and internal validation of a novel simple bedside risk score for elderly patients undergoing emergency surgery |  |
| **Frailty Index (FI)** | |  | **9 studies** |
|  | Aguilar-Frasco 2021 | Frailty index as a predictive preoperative tool in the elder population undergoing major abdominal surgery: a prospective analysis of clinical utility |  |
|  | Giannotti 2019 | Frailty assessment in elective gastrointestinal oncogeriatric surgery: Predictors of one-year mortality and functional status |  |
|  | Guo 2023 | Effects of sarcopenia and frailty on postoperative recovery in elderly patients: A prospective cohort study |  |
|  | Inci 2021 | Frailty Index for prediction of surgical outcome in ovarian cancer: Results of a prospective study |  |
|  | Joseph 2016 | Emergency General Surgery in the Elderly: Too Old or Too Frail? |  |
|  | Sakurai 2023 | Risk factors of "loss of independence" in elderly patients who received gastrectomy for gastric cancer |  |
|  | Yao 2019 | Frailty in ovarian cancer identified the need for increased postoperative care requirements following cytoreductive surgery |  |
|  | Yin 2021 | Comparison of three frailty measures for 90-day outcomes of elderly patients undergoing elective abdominal surgery |  |
|  | Yin 2022 | Which Frailty Evaluation Method Can Better Improve the Predictive Ability of the SASA for Postoperative Complications of Patients Undergoing Elective Abdominal Surgery? |  |
| **Fried Frailty Phenotype (FP)** | | | **26 studies** |
|  | Andreou 2018 | A Comparison of Two Preoperative Frailty Models in Predicting Postoperative Outcomes in Geriatric General Surgical Patients |  |
|  | Beckert 2017 | Screening for Frailty in Thoracic Surgical Patients |  |
|  | Burg 2019 | Frailty as a predictor of complications after radical cystectomy: A prospective study of various preoperative assessments |  |
|  | Courtney-Brooks 2012 | Frailty: an outcome predictor for elderly gynecologic oncology patients |  |
|  | Ding 2021 | Comparing the Performance of Different Instruments for Diagnosing Frailty and Predicting Adverse Outcomes among Elderly Patients with Gastric Cancer |  |
|  | Ding 2023 | Adverse outcomes and health-ecological influencing factors of preoperative frailty among elderly patients with gastric cancer |  |
|  | Fagard 2025 | Frailty screening in older patients undergoing elective colorectal surgery: Comparative study of seven screening instruments |  |
|  | Han 2018 | Predictive value of frailty on postoperative complications in elderly patients with major abdominal surgery |  |
|  | Kenig 2015b | Screening for frailty among older patients with cancer that qualify for abdominal surgery |  |
|  | Kenig 2020 | Usefulness of eight screening tools for predicting frailty and postoperative short- and long-term outcomes among older patients with cancer who qualify for abdominal surgery |  |
|  | Kinugasa 2024 | Fried Frailty Phenotype Questionnaire scores and postoperative patient-reported outcomes of patients undergoing major abdominal cancer surgery: A secondary analysis |  |
|  | Kristjansson 2012 | A comparison of two pre-operative frailty measures in older surgical cancer patients |  |
|  | Makary 2010 | Frailty as a predictor of surgical outcomes in older patients |  |
|  | Michalik 2022 | Prevalence of frailty syndrome in urological patients undergoing major elective surgical procedure due to malignancy |  |
|  | Parini 2024 | Comparison of frailty indexes as predictors of clinical outcomes after major thoracic surgery |  |
|  | Revenig 2013 | Too frail for surgery? Initial results of a large multidisciplinary prospective study examining preoperative variables predictive of poor surgical outcomes |  |
|  | Revenig 2014 | A prospective study examining the association between preoperative frailty and postoperative complications in patients undergoing minimally invasive surgery |  |
|  | Sikder 2019 | Postoperative Recovery in Frail, Pre-frail, and Non-frail Elderly Patients Following Abdominal Surgery |  |
|  | Su 2023 | Frailty combined with nutritional risk score in predicting postoperative complications of elderly patients with gastrointestinal malignancies |  |
|  | Tan 2012 | Assessment for frailty is useful for predicting morbidity in elderly patients undergoing colorectal cancer resection whose comorbidities are already optimized |  |
|  | Terrones 2020 | Factors associated with surgical complications in elderly patients with gastrointestinal neoplasms at the Centro Medico Naval |  |
|  | Than 2021 | Frailty and Adverse Outcomes Among Older Patients Undergoing Gastroenterological Surgery in Vietnam |  |
|  | Wunderle 2021 | Prospective geriatric assessment for perioperative risk stratification in partial nephrectomy |  |
|  | Xing 2023 | Preoperative prediction of adverse outcome after elective gastrointestinal surgery in older patients: three leading frailty instruments and the American Society of Anesthesiologists physical status |  |
|  | You 2027 | Application value of different frailty assessment tools in older patients undergoing major abdominal surgery |  |
|  | Zhu 2020 & 2021 | Frailty as a predictor of increased one-year mortality after elective non-cardiac surgery: A prospective cohort study |  |
| **Geriatric 8 (G8)** | |  | **26 studies** |
|  | Bessems 2021 | Frailty screening by Geriatric-8 and 4-meter gait speed test is feasible and predicts postoperative complications in elderly colorectal cancer patients |  |
|  | Fagard 2017 | Value of geriatric screening and assessment in predicting postoperative complications in patients older than 70 years undergoing surgery for colorectal cancer |  |
|  | Fagard 2030 | Frailty screening in older patients undergoing elective colorectal surgery: Comparative study of seven screening instruments |  |
|  | Hikita 2025 | Comparison of retroperitoneal laparoscopic radical nephroureterectomy outcomes in elderly patients based on the Geriatric 8 (G8) screening tool |  |
|  | Imasato 2025 | Predictive value of G8 screening tool for ambulation failure and severe complications in the perioperative period: Results from a large single-center cohort study |  |
|  | Kaibori 2016 | Geriatric assessment as a predictor of postoperative complications in elderly patients with hepatocellular carcinoma |  |
|  | Kaibori 2021 | Perioperative Geriatric Assessment as A Predictor of Long-Term Hepatectomy Outcomes in Elderly Patients with Hepatocellular Carcinoma |  |
|  | Kenig 2015a | Six screening instruments for frailty in older patients qualified for emergency abdominal surgery |  |
|  | Kenig 2015b | Screening for frailty among older patients with cancer that qualify for abdominal surgery |  |
|  | Kenig 2020 | Usefulness of eight screening tools for predicting frailty and postoperative short- and long-term outcomes among older patients with cancer who qualify for abdominal surgery |  |
|  | Ketelaers 2023 | An evaluation of postoperative outcomes and treatment changes after frailty screening and geriatric assessment and management in a cohort of older patients with colorectal cancer |  |
|  | Michalik 2022 | Prevalence of frailty syndrome in urological patients undergoing major elective surgical procedure due to malignancy |  |
|  | Montroni 2022 & Montroni 2023 | Quality of Life in Older Adults After Major Cancer Surgery: The GOSAFE International Study |  |
|  | Niemelainen 2021 | The Clinical Frailty Scale is a useful tool for predicting postoperative complications following elective colon cancer surgery at the age of 80 years and above: A prospective, multicentre observational study |  |
|  | Niemelainen 2022 | One-year functional outcomes of patients aged 80 years or more undergoing colonic cancer surgery: prospective, multicentre observational study |  |
|  | Nishijima 2021 | Preoperative frailty assessment with the Robinson Frailty Score, Edmonton Frail Scale, and G8 and adverse postoperative outcomes in older surgical patients with cancer |  |
|  | Park 2022 | Geriatric assessment using the G8 to predict postoperative complications in patients undergoing major uro-oncologic surgery: Comparison with the Charlson Comorbidity Index. |  |
|  | Souwer 2018 | Risk stratification for surgical outcomes in older colorectal cancer patients using ISAR-HP and G8 screening tools |  |
|  | Tamura 2021 | Optimal Assessment of Frailty Predicts Postoperative Complications in Older Patients with Colorectal Cancer Surgery |  |
|  | Terrones 2020 | Factors associated with surgical complications in elderly patients with gastrointestinal neoplasms at the Centro Medico Naval |  |
|  | Tokura 2023 | Geriatric 8 and Vulnerable Elders Survey-13 predict length of hospital stay and postoperative complications in Japanese patients undergoing urological surgery |  |
|  | Traunero 2022 | Reliable Prediction of Post-Operative Complications' Rate Using the G8 Screening Tool: A Prospective Study on Elderly Patients Undergoing Surgery for Kidney Cancer |  |
|  | Yajima 2022 | Predictive value of the G8 and the IADL-modified G8 screening tools for postoperative delirium following major urological cancer surgery |  |
|  |  | Value of Geriatric Assessment Using the G8 to Predict Postoperative Urinary Tract Infections in Patients Undergoing Radical Cystectomy |  |
|  | Yajima 2023 | Rapid geriatric screening tools predict inability to manage stoma by oneself after urinary diversion: G8 and IADL-modified G8 |  |
| **Geriatric Assessment in Partial Nephrectomy (GAPN)** | | | **1 study** |
|  | Wunderle 2021 | Prospective geriatric assessment for perioperative risk stratification in partial nephrectomy |  |
| **Geriatric Status Scale (GSS)** | | | **3 Studies** |
|  | Kenig 2015a | Six screening instruments for frailty in older patients qualified for emergency abdominal surgery |  |
|  | Kenig 2015b | Screening for frailty among older patients with cancer that qualify for abdominal surgery |  |
|  | Kenig 2020 | Usefulness of eight screening tools for predicting frailty and postoperative short- and long-term outcomes among older patients with cancer who qualify for abdominal surgery |  |
| **Groningen Frailty Indicator (GFI)** | | | **9 studies** |
|  | Argillander 2022 | The predictive value of preoperative frailty screening for postoperative outcomes in older patients undergoing surgery for non-metastatic colorectal cancer. |  |
|  | Huisman 2015 | Screening for predictors of adverse outcome in onco-geriatric surgical patients: A multicenter prospective cohort study |  |
|  | Kenig 2015a | Six screening instruments for frailty in older patients qualified for emergency abdominal surgery |  |
|  | Kenig 2015b | Screening for frailty among older patients with cancer that qualify for abdominal surgery |  |
|  | Kenig 2020 | Usefulness of eight screening tools for predicting frailty and postoperative short- and long-term outcomes among older patients with cancer who qualify for abdominal surgery |  |
|  | Reisinger 2015 | Functional compromise reflected by sarcopenia, frailty, and nutritional depletion predicts adverse postoperative outcome after colorectal cancer surgery |  |
|  | Tegels 2014 | Value of geriatric frailty and nutritional status assessment in predicting postoperative mortality in gastric cancer surgery |  |
|  | Ugolini 2015 | How to select elderly colorectal cancer patients for surgery: a pilot study in an Italian academic medical center |  |
|  | Wunderle 2021 | Prospective geriatric assessment for perioperative risk stratification in partial nephrectomy |  |
| **Identification of Seniors At Risk-Hospitalized Patients (ISAR-HP)** | | | **1 study** |
|  | Souwer 2018 | Risk stratification for surgical outcomes in older colorectal cancer patients using ISAR-HP and G8 screening tools |  |
| **Japanese version of the Cardiovascular Health Study (J-CHS)** | | | **1 study** |
|  | Kameyama 2021 | Efficacy of preoperative frailty assessment in patients with gastrointestinal disease |  |
| **Kihon Checklist (KCL)** | |  | **8 studies** |
|  | Ishihara 2021 | Preoperative Risk Assessment for Delirium After Hepatic Resection in the Elderly: a Prospective Multicenter Study |  |
|  | Kawaguchi 2025 | Risk assessment for loss of independence after hepatopancreaticobiliary surgery in the elderly: a prospective multicenter study |  |
|  | Okada 2023 | Impact of frailty on long-term outcomes after liver resection for hepatocellular carcinoma in elderly patients: A prospective study |  |
|  | Okada 2024 | Impact of frailty on long-term outcomes after liver resection for hepatocellular carcinoma in elderly patients: A prospective study |  |
|  | Tamura 2021 | Optimal Assessment of Frailty Predicts Postoperative Complications in Older Patients with Colorectal Cancer Surgery |  |
|  | Tanaka 2018 | Preoperative assessment of frailty predicts age-related events after hepatic resection: a prospective multicenter study |  |
|  | Tanaka 2021 | Preoperative Risk Assessment for Loss of Independence Following Hepatic Resection in Elderly Patients: A Prospective Multicenter Study |  |
|  | Ushigome 2025 | Frailty Recovery Following Minimally Invasive Surgery: An Emerging Perspective on Surgical Benefits in Elderly Colorectal Cancer Patients |  |
| **Memorial Sloan Kettering-Frailty Index (MSK-FI)** | | | **3 studies** |
|  | Hoffmann 2023 | Preoperative frailty vs. cognitive impairment: Which one matters most for postoperative delirium among older adults with cancer? |  |
|  | Poruk 2022 | Surgical resection for intraductal papillary mucinous neoplasm in the older population |  |
|  | Shahrokni 2019 | Development and Evaluation of a New Frailty Index for Older Surgical Patients With Cancer |  |
| **Modified Frailty Index - 11 item (mFI-11)** | | | **12 studies** |
|  | Aceto 2021 | Predictive power of modified frailty index score for pulmonary complications after major abdominal surgery in the elderly: a single centre prospective cohort study |  |
|  | Chambers 2021 | Modified frailty index predicts postoperative complications in women with gynecologic cancer undergoing cytoreductive surgery and hyperthermic intraperitoneal chemotherapy |  |
|  | Czajka 2023 | Is Frailty a Good Predictor of Postoperative Complications in Elective Abdominal Surgery?-A Single-Center, Prospective, Observational Study |  |
|  | Ding 2021 | Comparing the Performance of Different Instruments for Diagnosing Frailty and Predicting Adverse Outcomes among Elderly Patients with Gastric Cancer |  |
|  | Fagard 2031 | Frailty screening in older patients undergoing elective colorectal surgery: Comparative study of seven screening instruments |  |
|  | Parini 2024 | Comparison of frailty indexes as predictors of clinical outcomes after major thoracic surgery |  |
|  | Sacco 2018 | A new frailty index as a risk predictor of morbidity and mortality: Its application in a surgery unit |  |
|  | Suzuki 2021 | Effectiveness of frailty screening and perioperative team management of colectomy patients aged 80 years or more |  |
|  | Trostchansky 2019 | Is Norton Score a useful tool for identifying high-risk patients prior to emergency surgery? |  |
|  | Wunderle 2021 | Prospective geriatric assessment for perioperative risk stratification in partial nephrectomy |  |
|  | You 2025 | Application value of different frailty assessment tools in older patients undergoing major abdominal surgery |  |
|  | Zhu 2020 & 2021 | Frailty as a predictor of increased one-year mortality after elective non-cardiac surgery: A prospective cohort study |  |
| **modified Criteria for Screening and Triaging to Appropriate aLternative care (mCriSTAL)** | | | **1 study** |
|  | EguarasCordoba 2021 | External Prospective Validation of the Modified CriSTAL Score for 30- and 90-day Mortality in Geriatric Urgent Surgical Patients |  |
| **Multidimensional Prognostic Index (MPI)** | | | **1 study** |
|  | Pata 2021 | Multidimensional Prognostic Index (MPI) score has the major impact on outcome prediction in elderly surgical patients with colorectal cancer: The FRAGIS study |  |
| **New Surgical Frailty Index (nSFI)** | | | **1 study** |
|  | Sacco 2018 | A new frailty index as a risk predictor of morbidity and mortality: Its application in a surgery unit |  |
| **Norton Score** | |  | **1 study** |
|  | Trostchansky 2019 | Is Norton Score a useful tool for identifying high-risk patients prior to emergency surgery? |  |
| **Pictorial Fit Frail Scale (PFFS)** | | | **2 studies** |
|  | Cooper 2022 | Validation of the Pictorial Fit-Frail Scale in a Thoracic Surgery clinic. |  |
|  | Cooper 2023 | Validation of the Pictorial Fit-Frail Scale in a Thoracic Surgery clinic. |  |
| **PRISMA-7 Questionnaire** | |  | **1 study** |
|  | Serra-Pla 2020 | Multidisciplinary management and optimization of frail or high surgical risk patients in colorectal cancer surgery: Prospective observational analysis |  |
| **Risk Analysis Index - Clinical (RAI-C)** | | | **3 studies** |
|  | vanderWindt 2018 | Preoperative risk analysis index for frailty predicts short-term outcomes after hepatopancreatobiliary surgery |  |
|  | Varley 2020 | Clinical Utility of the Risk Analysis Index as a Prospective Frailty Screening Tool within a Multi-practice, Multi-hospital Integrated Healthcare System |  |
|  | Varley 2021 | Clinical Utility of the Risk Analysis Index as a Prospective Frailty Screening Tool within a Multi-practice, Multi-hospital Integrated Healthcare System |  |
| **Robinson Frailty Score (RFS)** | | | **3 studies** |
|  | Fagard 2026 | Frailty screening in older patients undergoing elective colorectal surgery: Comparative study of seven screening instruments |  |
|  | Nishijima 2021 | Preoperative frailty assessment with the Robinson Frailty Score, Edmonton Frail Scale, and G8 and adverse postoperative outcomes in older surgical patients with cancer |  |
|  | Robinson 2013 | Simple frailty score predicts postoperative complications across surgical specialties |  |
| **Share-FI** | |  | **1 study** |
|  | Arteaga 2021 | Impact of frailty in surgical emergencies. A comparison of four frailty scales |  |
| **Simplified Frailty Score (novel including some Fried Criteria)** | | | **1 study** |
|  | Revenig 2015 | Report of a Simplified Frailty Score Predictive of Short-Term Postoperative Morbidity and Mortality |  |
| **Study of Osteoporotic Fractures (SOF) index** | | | **3 studies** |
|  | Choe 2017 | Association between frailty and readmission within one year after gastrectomy in older patients with gastric cancer |  |
|  | Jeong 2021 | Relationship between frailty and mortality after gastrectomy in older patients with gastric cancer |  |
|  | Jeong 2022 | Relationship between frailty and mortality after gastrectomy in older patients with gastric cancer |  |
| **Tilburg Frailty Indicator (TFI)** | | | **1 study** |
|  | Ding 2021 | Comparing the Performance of Different Instruments for Diagnosing Frailty and Predicting Adverse Outcomes among Elderly Patients with Gastric Cancer |  |
| **Triage Risk Screening Tool (TRST)** | | | **10 studies** |
|  | Arteaga 2021 | Impact of frailty in surgical emergencies. A comparison of four frailty scales |  |
|  | Fagard 2017 | Value of geriatric screening and assessment in predicting postoperative complications in patients older than 70 years undergoing surgery for colorectal cancer |  |
|  | Kenig 2015a | Six screening instruments for frailty in older patients qualified for emergency abdominal surgery |  |
|  | Kenig 2015b | Screening for frailty among older patients with cancer that qualify for abdominal surgery |  |
|  | Kenig 2020 | Usefulness of eight screening tools for predicting frailty and postoperative short- and long-term outcomes among older patients with cancer who qualify for abdominal surgery |  |
|  | Montroni 2022 & Montroni 2023  (two reports using same study population) | Quality of Life in Older Adults After Major Cancer Surgery: The GOSAFE International Study |  |
|  | SanchezArteaga 2022 | Long-term influence of frailty in elderly patients after surgical emergencies |  |
|  | Taffurelli 2023 | Frailty assessment can predict textbook outcomes in senior adults after minimally invasive colorectal cancer surgery |  |
|  | Zattoni 2019 | A Simple Screening Tool to Predict Outcomes in Older Adults Undergoing Emergency General Surgery |  |
|  | Zattoni 2021 | Prediction of functional loss in emergency surgery is possible with a simple frailty screening tool |  |
| **VMS-questionnaire (VeiligheidsManagementSysteem) (Dutch Safety Management Program)** | | | **3 Studies** |
|  | Argillander 2022 | Predictive value of selected geriatric parameters for postoperative outcomes in older patients with rectal cancer - A multicenter cohort study. |  |
|  | Souwer 2019 | The Prognostic Value of a Geriatric Risk Score for Older Patients with Colorectal Cancer |  |
|  | vanderZanden 2021 | Risk assessment for postoperative outcomes in a mixed hospitalized gynecological population by the Dutch safety management system (Veiligheidsmanagementsysteem, VMS) screening tool 'frail elderly' |  |
| **Vulnerable Elderly Surgical Pathways and Outcomes Assessment (VESPA)** | | | **1 study** |
|  | Pollock 2020 | A novel geriatric assessment tool that predicts postoperative complications in older adults with cancer |  |
| **Vulnerable Elderly Survey (VES-13)** | | | **13 studies** |
|  | Dale 2014 | Geriatric assessment improves prediction of surgical outcomes in older adults undergoing pancreaticoduodenectomy: a prospective cohort study |  |
|  | Huisman 2015 | Screening for predictors of adverse outcome in onco-geriatric surgical patients: A multicenter prospective cohort study |  |
|  | Kaibori 2016 | Geriatric assessment as a predictor of postoperative complications in elderly patients with hepatocellular carcinoma |  |
|  | Kaibori 2021 | Perioperative Geriatric Assessment as A Predictor of Long-Term Hepatectomy Outcomes in Elderly Patients with Hepatocellular Carcinoma |  |
|  | Kenig 2014 | Vulnerable Elderly Survey 13 as a screening method for frailty in Polish elderly surgical patient--prospective study |  |
|  | Kenig 2015a | Six screening instruments for frailty in older patients qualified for emergency abdominal surgery |  |
|  | Kenig 2015b | Screening for frailty among older patients with cancer that qualify for abdominal surgery |  |
|  | Kenig 2020 | Usefulness of eight screening tools for predicting frailty and postoperative short- and long-term outcomes among older patients with cancer who qualify for abdominal surgery |  |
|  | Michalik 2022 | Prevalence of frailty syndrome in urological patients undergoing major elective surgical procedure due to malignancy |  |
|  | Terrones 2020 | Factors associated with surgical complications in elderly patients with gastrointestinal neoplasms at the Centro Medico Naval |  |
|  | Tokura 2023 | Geriatric 8 and Vulnerable Elders Survey-13 predict length of hospital stay and postoperative complications in Japanese patients undergoing urological surgery |  |
|  | Ugolini 2015 | How to select elderly colorectal cancer patients for surgery: a pilot study in an Italian academic medical center |  |
|  | Vernon 2019 | Implementation of Vulnerable Elders Survey-13 Frailty Tool to Identify At-Risk Geriatric Surgical Patients |  |

**Supplementary** Figure S2. Frailty Assessment Tools by Setting and Surgical Type**.** Counts of studies and participants by setting are presented in panel A and by surgical type in panel B. Color scale indicates the number of participants, highlighting the extent of data available for each setting and tool. This visualization focuses on the 12 most extensively investigated tools; additional tools can be found in Supplementary Table S3 and Supplementary Table S4. *The “All Tools” row includes the total number of studies and participants for each category, encompassing both visualized and excluded tools not meeting criteria for visualization.

# Supplementary Table S3. Frailty Assessment Tools by Setting

| **Frailty Tool** | **Total**  No. Studies  (Participants) | **Oncology**  No. Studies  (Participants | **Benign**  No. Studies  (Participants | **Emergency**  No. Studies  (Participants |
| --- | --- | --- | --- | --- |
| All Tools* | 35 (57549) | 29 (48811) | 21 (30275) | 16 (11886) |
| CFS | 41 (9637) | 21 (4389) | 8 (1545) | 16 (4359) |
| G8 | 26 (8108) | 21 (5746) | 2 (830) | 1 (184) |
| FP | 26 (5627) | 20 (4208) | 16 (3687) | 0 (0) |
| FRAIL | 15 (4377) | 12 (2138) | 9 (3030) | 4 (1788) |
| EFS | 15 (3687) | 5 (1960) | 10 (2425) | 3 (1441) |
| VES-13 | 13 (2322) | 7 (2464) | 4 (873) | 1 (184) |
| mFI-11 | 12 (2525) | 7 (1406) | 9 (2104) | 1 (129) |
| TRST | 10 (2516) | 8 (1652) | 0 (0) | 5 (556) |
| FI | 9 (2331) | 9 (1847) | 4 (1102) | 2 (360) |
| GFI | 9 (1836) | 7 (1537) | 1 (150) | 1 (184) |
| KCL | 8 (1961) | 5 (1440) | 4 (1060) | 0 (0) |
| RAI-C | 3 (11287) | 3 (11287) | 3 (11287) | 0 (0) |
| SOF | 3 (685) | 2 (407) | 0 (0) | 0 (0) |
| RFS | 3 (373) | 3 (685) | 2 (259) | 0 (0) |
| GSS | 3 (591) | 2 (407) | 0 (0) | 1 (184) |
| Balducci | 3 (591) | 3 (3092) | 0 (0) | 1 (184) |
| MSK-FI | 3 (3092) | 3 (1282) | 1 (148) | 0 (0) |
| VMS | 3 (1282) | 2 (186) | 1 (157) | 0 (0) |
| PFFS | 2 (95) | 2 (407) | 1 (49) | 0 (0) |
| aCGA | 2 (407) | 0 (0) | 0 (0) | 0 (0) |
| EGSFI | 2 (586) | 0 (0) | 0 (0) | 2 (586) |
| Share-FI | 1 (92) | 0 (0) | 0 (0) | 1 (92) |
| J-CHS | 1 (42) | 1 (104) | 1 (42) | 0 (0) |
| FRAILESEL | 1 (1024) | 1 (139) | 0 (0) | 1 (1024) |
| GAPN | 1 (150) | 1 (311) | 1 (150) | 0 (0) |
| SFS | 1 (351) | 1 (351) | 1 (351) | 0 (0) |
| MPI | 1 (104) | 1 (476) | 0 (0) | 0 (0) |
| EFT | 1 (311) | 1 (42) | 1 (311) | 0 (0) |
| mCriSTAL | 1 (500) | 1 (150) | 0 (0) | 1 (500) |
| VESPA | 1 (476) | 0 (0) | 0 (0) | 0 (0) |
| ISAR-HP | 1 (139) | 1 (49) | 0 (0) | 0 (0) |
| PRISMA-7 | 1 (193) | 1 (193) | 0 (0) | 0 (0) |
| Norton | 1 (129) | 0 (0) | 0 (0) | 1 (129) |
| nSFI | 1 (456) | 1 (456) | 1 (456) | 0 (0) |
| TFI | 1 (259) | 0 (0) | 1 (259) | 0 (0) |

*The all tools row includes the total number of tools and participants, not the number of studies, for each property.

# Supplementary Table S4. Frailty Assessment Tools by Surgery Type

| **Frailty Tool** | **Total** | **General** | **GI** | **HPB** | **Colorectal** | **Thoracics** | **Urology** | **Gynecology** |
| --- | --- | --- | --- | --- | --- | --- | --- | --- |
| All Tools* | 35 (68142) | 24 (31977) | 24 (24089) | 20 (17705) | 25 (25502) | 12 (18129) | 15 (23384) | 13 (17616) |
| CFS | 41 (9637) | 17 (4323) | 9 (1934) | 8 (1279) | 15 (3709) | 1 (59) | 5 (782) | 2 (388) |
| FP | 26 (5627) | 9 (2490) | 14 (3391) | 8 (1860) | 10 (1866) | 2 (436) | 6 (921) | 1 (37) |
| G8 | 26 (8108) | 3 (570) | 6 (1892) | 6 (1634) | 12 (3196) | 1 (942) | 8 (2561) | 1 (114) |
| EFS | 15 (3687) | 6 (1666) | 6 (1162) | 4 (788) | 4 (507) | 0 (0) | 3 (745) | 2 (248) |
| FRAIL | 15 (4377) | 9 (2739) | 4 (1094) | 3 (885) | 3 (592) | 2 (394) | 3 (555) | 2 (388) |
| VES-13 | 13 (2322) | 2 (456) | 6 (1270) | 7 (1088) | 6 (1071) | 0 (0) | 3 (1039) | 1 (328) |
| mFI-11 | 12 (2525) | 5 (1223) | 6 (1481) | 4 (1093) | 6 (1120) | 1 (311) | 3 (558) | 2 (238) |
| TRST | 10 (2516) | 6 (828) | 4 (1533) | 3 (1349) | 6 (2144) | 1 (942) | 0 (0) | 0 (0) |
| FI | 9 (2331) | 4 (748) | 4 (664) | 2 (334) | 2 (263) | 1 (140) | 2 (388) | 5 (1207) |
| GFI | 9 (1836) | 2 (456) | 5 (1099) | 3 (735) | 7 (1506) | 0 (0) | 2 (478) | 1 (328) |
| KCL | 8 (1961) | 0 (0) | 0 (0) | 6 (1222) | 2 (739) | 0 (0) | 0 (0) | 0 (0) |
| RAI-C | 3 (11287) | 2 (11125) | 0 (0) | 1 (162) | 0 (0) | 2 (11125) | 2 (11125) | 2 (11125) |
| Balducci | 3 (591) | 2 (456) | 3 (591) | 2 (407) | 3 (591) | 0 (0) | 0 (0) | 0 (0) |
| GSS | 3 (591) | 2 (456) | 3 (591) | 2 (407) | 3 (591) | 0 (0) | 0 (0) | 0 (0) |
| MSK-FI | 3 (3092) | 0 (0) | 2 (2944) | 3 (3092) | 2 (2944) | 2 (2944) | 2 (2944) | 2 (2944) |
| RFS | 3 (373) | 1 (114) | 1 (114) | 1 (114) | 2 (259) | 0 (0) | 0 (0) | 1 (114) |
| SOF | 3 (685) | 0 (0) | 3 (685) | 0 (0) | 0 (0) | 0 (0) | 0 (0) | 0 (0) |
| VMS | 3 (1282) | 0 (0) | 0 (0) | 0 (0) | 2 (1125) | 0 (0) | 0 (0) | 1 (157) |
| aCGA | 2 (407) | 1 (272) | 2 (407) | 2 (407) | 2 (407) | 0 (0) | 0 (0) | 0 (0) |
| EGSFI | 2 (586) | 2 (586) | 0 (0) | 0 (0) | 0 (0) | 0 (0) | 0 (0) | 0 (0) |
| PFFS | 2 (95) | 0 (0) | 0 (0) | 0 (0) | 0 (0) | 1 (49) | 0 (0) | 0 (0) |
| EFT | 1 (311) | 1 (311) | 0 (0) | 0 (0) | 0 (0) | 1 (311) | 1 (311) | 0 (0) |
| FRAILESEL | 1 (1024) | 1 (1024) | 1 (1024) | 0 (0) | 1 (1024) | 0 (0) | 0 (0) | 0 (0) |
| GAPN | 1 (150) | 0 (0) | 0 (0) | 0 (0) | 0 (0) | 0 (0) | 1 (150) | 0 (0) |
| ISAR-HP | 1 (139) | 0 (0) | 0 (0) | 0 (0) | 1 (139) | 0 (0) | 0 (0) | 0 (0) |
| J-CHS | 1 (42) | 0 (0) | 1 (42) | 1 (42) | 0 (0) | 0 (0) | 0 (0) | 0 (0) |
| mCriSTAL | 1 (500) | 1 (500) | 1 (500) | 0 (0) | 0 (0) | 0 (0) | 0 (0) | 0 (0) |
| MPI | 1 (104) | 0 (0) | 0 (0) | 0 (0) | 1 (104) | 0 (0) | 0 (0) | 0 (0) |
| Norton | 1 (129) | 0 (0) | 1 (129) | 0 (0) | 1 (129) | 0 (0) | 0 (0) | 0 (0) |
| nSFI | 1 (456) | 1 (456) | 1 (456) | 1 (456) | 1 (456) | 0 (0) | 0 (0) | 0 (0) |
| PRISMA-7 | 1 (193) | 0 (0) | 0 (0) | 0 (0) | 1 (193) | 0 (0) | 0 (0) | 0 (0) |
| SFS | 1 (351) | 1 (351) | 1 (351) | 1 (351) | 1 (351) | 0 (0) | 1 (351) | 0 (0) |
| Share-FI | 1 (92) | 1 (92) | 0 (0) | 0 (0) | 0 (0) | 0 (0) | 0 (0) | 0 (0) |
| VESPA | 1 (476) | 1 (476) | 1 (476) | 0 (0) | 1 (476) | 1 (476) | 1 (476) | 0 (0) |
| TFI | 1 (259) | 1 (259) | 1 (259) | 0 (0) | 0 (0) | 0 (0) | 0 (0) | 0 (0) |

*The all tools row includes the total number of tools and participants, not the number of studies, for each property.

# Supplementary Table S5. Performance Metrics of Frailty Assessment Tools

| **Frailty Tool** | **Total**  No. Studies (Participants) | | **Construct Validity**  No. Studies (Participants) | **Concurrent Criterion**  No. Studies (Participants) | **Predictive validity**  No. Studies  (Participants) | **Reliability**  No. Studies (Participants) | **Clinical Impact**  No. Studies (Participants) | **Pragmatic Factors**  No. Studies (Participants) |
| --- | --- | --- | --- | --- | --- | --- | --- | --- |
| All Tools* | 35 (68142) | 12 (5721) | | 12 (6388) | 33 (64527) | 2 (546) | 7 (3307) | 8 (10045) |
| CFS | 41 (9637) | 5 (1593) | | 2 (412) | 41 (9637) | 2 (412) | 0 (0) | 0 (0) |
| G8 | 26 (8108) | 4 (1587) | | 4 (1118) | 25 (7973) | 0 (0) | 1 (170) | 0 (0) |
| FP | 26 (5627) | 2 (301) | | 5 (898) | 24 (5367) | 0 (0) | 1 (259) | 0 (0) |
| FRAIL | 15 (4377) | 1 (194) | | 1 (194) | 14 (3219) | 0 (0) | 1 (1158) | 1 (1158) |
| EFS | 15 (3687) | 3 (410) | | 1 (218) | 13 (2829) | 1 (134) | 2 (858) | 1 (134) |
| VES-13 | 13 (2322) | 2 (428) | | 5 (1224) | 11 (2081) | 0 (0) | 0 (0) | 1 (48) |
| mFI-11 | 12 (2525) | 0 (0) | | 0 (0) | 12 (2525) | 0 (0) | 2 (410) | 1 (151) |
| TRST | 10 (2516) | 0 (0) | | 2 (407) | 9 (2381) | 0 (0) | 0 (0) | 0 (0) |
| FI | 9 (2331) | 2 (334) | | 1 (194) | 9 (2331) | 0 (0) | 0 (0) | 1 (140) |
| GFI | 9 (1836) | 1 (328) | | 2 (407) | 8 (1701) | 0 (0) | 0 (0) | 0 (0) |
| KCL | 8 (1961) | 0 (0) | | 0 (0) | 8 (1961) | 0 (0) | 0 (0) | 0 (0) |
| RAI-C | 3 (11287) | 0 (0) | | 0 (0) | 3 (11287) | 0 (0) | 0 (0) | 1 (8172) |
| SOF | 3 (685) | 0 (0) | | 0 (0) | 3 (685) | 0 (0) | 0 (0) | 0 (0) |
| RFS | 3 (373) | 0 (0) | | 0 (0) | 3 (373) | 0 (0) | 0 (0) | 0 (0) |
| GSS | 3 (591) | 0 (0) | | 2 (407) | 2 (456) | 0 (0) | 0 (0) | 0 (0) |
| Balducci | 3 (591) | 0 (0) | | 2 (407) | 2 (456) | 0 (0) | 0 (0) | 0 (0) |
| MSK-FI | 3 (3092) | 0 (0) | | 0 (0) | 3 (3092) | 0 (0) | 0 (0) | 0 (0) |
| VMS | 3 (1282) | 0 (0) | | 0 (0) | 3 (1282) | 0 (0) | 0 (0) | 0 (0) |
| PFFS | 2 (95) | 1 (49) | | 2 (95) | 0 (0) | 0 (0) | 0 (0) | 1 (49) |
| aCGA | 2 (407) | 0 (0) | | 2 (407) | 1 (272) | 0 (0) | 0 (0) | 0 (0) |
| EGSFI | 2 (586) | 0 (0) | | 0 (0) | 2 (586) | 0 (0) | 0 (0) | 0 (0) |
| Share-FI | 1 (92) | 0 (0) | | 0 (0) | 1 (92) | 0 (0) | 0 (0) | 0 (0) |
| J-CHS | 1 (42) | 1 (42) | | 0 (0) | 1 (42) | 0 (0) | 0 (0) | 0 (0) |
| FRAILESEL | 1 (1024) | 0 (0) | | 0 (0) | 1 (1024) | 0 (0) | 0 (0) | 0 (0) |
| GAPN | 1 (150) | 0 (0) | | 0 (0) | 1 (150) | 0 (0) | 0 (0) | 0 (0) |
| SFS | 1 (351) | 1 (351) | | 0 (0) | 1 (351) | 0 (0) | 0 (0) | 0 (0) |
| MPI | 1 (104) | 1 (104) | | 0 (0) | 1 (104) | 0 (0) | 0 (0) | 0 (0) |
| TFI | 1 (259) | 0 (0) | | 0 (0) | 1 (259) | 0 (0) | 1 (259) | 0 (0) |
| EFT | 1 (311) | 0 (0) | | 0 (0) | 1 (311) | 0 (0) | 0 (0) | 0 (0) |
| VESPA | 1 (476) | 0 (0) | | 0 (0) | 1 (476) | 0 (0) | 0 (0) | 0 (0) |
| ISAR-HP | 1 (139) | 0 (0) | | 0 (0) | 1 (139) | 0 (0) | 0 (0) | 0 (0) |
| PRISMA-7 | 1 (193) | 0 (0) | | 0 (0) | 0 (0) | 0 (0) | 1 (193) | 1 (193) |
| Norton | 1 (129) | 0 (0) | | 0 (0) | 1 (129) | 0 (0) | 0 (0) | 0 (0) |
| nSFI | 1 (456) | 0 (0) | | 0 (0) | 1 (456) | 0 (0) | 0 (0) | 0 (0) |
| mCriSTAL | 1 (500) | 0 (0) | | 0 (0) | 1 (500) | 0 (0) | 0 (0) | 0 (0) |

*The all tools row includes the total number of tools and participants, not the number of studies, for each property.

# Supplementary Table S6. Type of Investigation of Measurement Properties

| **Frailty Tool** | **aCGA** | **Balducci** | **CFS** | **EFS** | **EFT** | **EGSFI** | **FI** | **FP** | **FRAIL** | **FRAILESEL** | **G8** | **GAPN** | **GFI** | **GSS** | **ISAR-HP** | **J-CHS** | **KCL** |
| --- | --- | --- | --- | --- | --- | --- | --- | --- | --- | --- | --- | --- | --- | --- | --- | --- | --- |
| Total No. Studies | 2 | 3 | 41 | 15 | 1 | 2 | 9 | 26 | 15 | 1 | 26 | 1 | 9 | 3 | 1 | 1 | 8 |
| **Predictive validity** | 1 | 2 | 41 | 13 | 1 | 2 | 9 | 24 | 14 | 1 | 25 | 1 | 8 | 2 | 1 | 1 | 8 |
| Exploratory | 1 | 2 | 34 | 9 | 1 | 2 | 6 | 20 | 11 | 0 | 22 | 0 | 8 | 2 | 1 | 1 | 8 |
| Confirmatory | 0 | 0 | 7 | 3 | 0 | 0 | 2 | 3 | 3 | 0 | 3 | 0 | 0 | 0 | 0 | 0 | 0 |
| Meta-Analysis | 0 | 0 | 3 | 0 | 0 | 0 | 0 | 0 | 0 | 0 | 3 | 0 | 0 | 0 | 0 | 0 | 0 |
| Incremental Value | 0 | 0 | 1 | 1 | 0 | 0 | 2 | 1 | 2 | 0 | 0 | 0 | 0 | 0 | 0 | 0 | 0 |
| Development/Internal Validation | 0 | 0 | 0 | 0 | 0 | 0 | 0 | 0 | 0 | 1 | 0 | 1 | 0 | 0 | 0 | 0 | 0 |
| External Validation | 0 | 0 | 0 | 0 | 0 | 0 | 0 | 0 | 0 | 0 | 0 | 0 | 0 | 0 | 0 | 0 | 0 |
| Only Exploratory Assessment of Tool Across Studies | 1 | 1 | 0 | 0 | 1 | 1 | 0 | 0 | 0 | 0 | 0 | 0 | 1 | 1 | 1 | 1 | 1 |
| **Concurrent Criterion** | 2 | 2 | 2 | 1 | 0 | 0 | 1 | 5 | 1 | 0 | 4 | 0 | 2 | 2 | 0 | 0 | 0 |
| Screening | 2 | 2 | 2 | 1 | 0 | 0 | 1 | 5 | 1 | 0 | 4 | 0 | 2 | 2 | 0 | 0 | 0 |
| Diagnosis | 0 | 0 | 0 | 0 | 0 | 0 | 0 | 0 | 0 | 0 | 0 | 0 | 0 | 0 | 0 | 0 | 0 |
| Agreement/Concordance (Exploratory) | 0 | 0 | 2 | 1 | 0 | 0 | 1 | 2 | 1 | 0 | 1 | 0 | 0 | 0 | 0 | 0 | 0 |
| Indirect Comparison | 0 | 0 | 1 | 1 | 0 | 0 | 0 | 0 | 0 | 0 | 0 | 0 | 0 | 0 | 0 | 0 | 0 |
| Direct Comparison | 2 | 2 | 0 | 1 | 0 | 0 | 0 | 3 | 0 | 0 | 3 | 0 | 2 | 2 | 0 | 0 | 0 |
| Only Exploratory Assessment of Tool Across Studies | 0 | 0 | 0 | 0 | 1 | 1 | 1 | 0 | 1 | 1 | 0 | 1 | 0 | 0 | 1 | 1 | 1 |
| **Clinical Impact** | 0 | 0 | 0 | 2 | 0 | 0 | 0 | 1 | 1 | 0 | 1 | 0 | 0 | 0 | 0 | 0 | 0 |
| Guide Clinical Decision | 0 | 0 | 0 | 1 | 0 | 0 | 0 | 1 | 1 | 0 | 0 | 0 | 0 | 0 | 0 | 0 | 0 |
| Estimate Treatment Effect | 0 | 0 | 0 | 0 | 0 | 0 | 0 | 0 | 0 | 0 | 0 | 0 | 0 | 0 | 0 | 0 | 0 |
| **Pragmatic Factors** | 0 | 0 | 0 | 1 | 0 | 0 | 1 | 0 | 1 | 0 | 0 | 0 | 0 | 0 | 0 | 0 | 0 |
| Acceptability | 0 | 0 | 0 | 1 | 0 | 0 | 0 | 0 | 0 | 0 | 0 | 0 | 0 | 0 | 0 | 0 | 0 |
| Ease | 0 | 0 | 0 | 1 | 0 | 0 | 1 | 0 | 1 | 0 | 0 | 0 | 0 | 0 | 0 | 0 | 0 |
| Compatibility | 0 | 0 | 0 | 0 | 0 | 0 | 0 | 0 | 0 | 0 | 0 | 0 | 0 | 0 | 0 | 0 | 0 |
| Usefulness | 0 | 0 | 0 | 0 | 0 | 0 | 0 | 0 | 1 | 0 | 0 | 0 | 0 | 0 | 0 | 0 | 0 |
| Only Ease Assessed for Tool Across Studies | 0 | 0 | 0 | 0 | 0 | 0 | 1 | 0 | 0 | 0 | 0 | 0 | 0 | 0 | 0 | 0 | 0 |

Table continues on next page…

Table continued…

| **Frailty Tool** | **mCriSTAL** | **mFI-11** | **MPI** | **MSK-FI** | **Norton** | **nSFI** | **PFFS** | **PRISMA-7** | **RAI-C** | **RFS** | **SFS** | **Share-FI** | **SOF** | **TFI** | **TRST** | **VES-13** | **VESPA** | **VMS** |
| --- | --- | --- | --- | --- | --- | --- | --- | --- | --- | --- | --- | --- | --- | --- | --- | --- | --- | --- |
| Total No. Studies | 1 | 12 | 1 | 3 | 1 | 1 | 2 | 1 | 3 | 3 | 1 | 1 | 3 | 1 | 10 | 13 | 1 | 3 |
| **Predictive validity** | 1 | 12 | 1 | 3 | 1 | 1 | 0 | 0 | 3 | 3 | 1 | 1 | 3 | 1 | 9 | 11 | 1 | 3 |
| Exploratory | 0 | 10 | 0 | 1 | 1 | 0 | 0 | 0 | 0 | 1 | 0 | 1 | 3 | 1 | 9 | 9 | 0 | 2 |
| Confirmatory | 0 | 1 | 1 | 1 | 0 | 0 | 0 | 0 | 1 | 0 | 0 | 0 | 0 | 0 | 0 | 2 | 1 | 1 |
| Meta-Analysis | 0 | 0 | 0 | 0 | 0 | 0 | 0 | 0 | 0 | 0 | 0 | 0 | 0 | 0 | 1 | 0 | 0 | 0 |
| Incremental Value | 0 | 0 | 0 | 0 | 0 | 0 | 0 | 0 | 0 | 1 | 0 | 0 | 0 | 0 | 0 | 0 | 0 | 0 |
| Development/Internal Validation | 0 | 0 | 0 | 1 | 0 | 1 | 0 | 0 | 0 | 1 | 1 | 0 | 0 | 0 | 0 | 0 | 0 | 0 |
| External Validation | 1 | 0 | 0 | 0 | 0 | 0 | 0 | 0 | 3 | 0 | 0 | 0 | 0 | 0 | 0 | 0 | 0 | 0 |
| Only Exploratory Assessment of Tool Across Studies | 0 | 0 | 0 | 0 | 1 | 0 | 1 | 1 | 0 | 0 | 0 | 1 | 1 | 1 | 1 | 0 | 0 | 0 |
| **Concurrent Criterion** | 0 | 0 | 0 | 0 | 0 | 0 | 2 | 0 | 0 | 0 | 0 | 0 | 0 | 0 | 2 | 5 | 0 | 0 |
| Screening | 0 | 0 | 0 | 0 | 0 | 0 | 2 | 0 | 0 | 0 | 0 | 0 | 0 | 0 | 2 | 5 | 0 | 0 |
| Diagnosis | 0 | 0 | 0 | 0 | 0 | 0 | 0 | 0 | 0 | 0 | 0 | 0 | 0 | 0 | 0 | 0 | 0 | 0 |
| Agreement/Concordance (Exploratory) | 0 | 0 | 0 | 0 | 0 | 0 | 2 | 0 | 0 | 0 | 0 | 0 | 0 | 0 | 0 | 1 | 0 | 0 |
| Indirect Comparison | 0 | 0 | 0 | 0 | 0 | 0 | 0 | 0 | 0 | 0 | 0 | 0 | 0 | 0 | 0 | 0 | 0 | 0 |
| Direct Comparison | 0 | 0 | 0 | 0 | 0 | 0 | 0 | 0 | 0 | 0 | 0 | 0 | 0 | 0 | 2 | 4 | 0 | 0 |
| Only Exploratory Assessment of Tool Across Studies | 1 | 1 | 1 | 1 | 1 | 1 | 1 | 1 | 1 | 1 | 1 | 1 | 1 | 1 | 0 | 0 | 1 | 1 |
| **Clinical Impact** | 0 | 2 | 0 | 0 | 0 | 0 | 0 | 1 | 0 | 0 | 0 | 0 | 0 | 1 | 0 | 0 | 0 | 0 |
| Guide Clinical Decision | 0 | 2 | 0 | 0 | 0 | 0 | 0 | 1 | 0 | 0 | 0 | 0 | 0 | 1 | 0 | 0 | 0 | 0 |
| Differential Treatment Effect | 0 | 0 | 0 | 0 | 0 | 0 | 0 | 0 | 0 | 0 | 0 | 0 | 0 | 0 | 0 | 0 | 0 |  |
| **Pragmatic Factors** | 0 | 1 | 0 | 0 | 0 | 0 | 1 | 1 | 1 | 0 | 0 | 0 | 0 | 0 | 0 | 1 | 0 | 0 |
| Acceptability | 0 | 0 | 0 | 0 | 0 | 0 | 0 | 0 | 0 | 0 | 0 | 0 | 0 | 0 | 0 | 1 | 0 | 0 |
| Ease | 0 | 0 | 0 | 0 | 0 | 0 | 1 | 0 | 1 | 0 | 0 | 0 | 0 | 0 | 0 | 1 | 0 | 0 |
| Compatibility | 0 | 0 | 0 | 0 | 0 | 0 | 0 | 0 | 0 | 0 | 0 | 0 | 0 | 0 | 0 | 1 | 0 | 0 |
| Usefulness | 0 | 1 | 0 | 0 | 0 | 0 | 0 | 1 | 0 | 0 | 0 | 0 | 0 | 0 | 0 | 1 | 0 | 0 |
| Only Ease Assessed for Tool Across Studies | 0 | 0 | 0 | 0 | 0 | 0 | 1 | 0 | 1 | 0 | 0 | 0 | 0 | 0 | 0 | 0 | 0 | 0 |

# Supplementary Table S7. Number of Studies Reporting Outcomes for Predictive validity

| **Frailty Tool** | **Total No. Studies** | **Death** | **Major Complication** | **Failure to Rescue** | **Any Complication** | **ICU Admission** | **Length of Stay** | **Readmission** | **Discharge Not Home** |
| --- | --- | --- | --- | --- | --- | --- | --- | --- | --- |
| All Tools* | 35 | 26 | 16 | 5 | 29 | 9 | 17 | 18 | 13 |
| CFS | 41 | 11 | 8 | 0 | 16 | 3 | 11 | 4 | 5 |
| FP | 26 | 3 | 4 | 1 | 21 | 1 | 6 | 4 | 2 |
| G8 | 26 | 5 | 2 | 0 | 17 | 0 | 5 | 2 | 1 |
| EFS | 15 | 4 | 1 | 0 | 10 | 2 | 9 | 1 | 3 |
| FRAIL | 15 | 3 | 3 | 0 | 6 | 0 | 3 | 1 | 1 |
| VES-13 | 13 | 2 | 3 | 0 | 7 | 1 | 2 | 1 | 2 |
| mFI-11 | 12 | 3 | 2 | 1 | 9 | 1 | 3 | 1 | 0 |
| TRST | 10 | 5 | 1 | 0 | 5 | 0 | 1 | 1 | 0 |
| FI | 9 | 4 | 2 | 0 | 5 | 2 | 5 | 1 | 0 |
| GFI | 9 | 5 | 3 | 1 | 5 | 0 | 1 | 1 | 1 |
| KCL | 8 | 1 | 0 | 0 | 2 | 0 | 2 | 0 | 1 |
| Balducci | 3 | 2 | 0 | 0 | 2 | 0 | 0 | 0 | 0 |
| GSS | 3 | 2 | 0 | 0 | 2 | 0 | 0 | 0 | 0 |
| MSK-FI | 3 | 0 | 1 | 0 | 2 | 1 | 2 | 1 | 0 |
| RAI-C | 3 | 2 | 0 | 0 | 1 | 2 | 3 | 3 | 1 |
| RFS | 3 | 1 | 1 | 0 | 3 | 0 | 2 | 1 | 1 |
| SOF | 3 | 1 | 0 | 0 | 0 | 0 | 0 | 1 | 0 |
| VMS | 3 | 1 | 1 | 0 | 3 | 0 | 1 | 1 | 2 |
| aCGA | 2 | 1 | 0 | 0 | 1 | 0 | 0 | 0 | 0 |
| EGSFI | 2 | 1 | 0 | 1 | 1 | 0 | 0 | 1 | 1 |
| PFFS | 2 | 0 | 0 | 0 | 0 | 0 | 0 | 0 | 0 |
| EFT | 1 | 1 | 0 | 0 | 0 | 1 | 0 | 0 | 0 |
| FRAILESEL | 1 | 1 | 0 | 0 | 1 | 0 | 0 | 0 | 0 |
| GAPN | 1 | 0 | 1 | 1 | 1 | 0 | 0 | 1 | 0 |
| ISAR-HP | 1 | 1 | 0 | 0 | 1 | 0 | 1 | 1 | 0 |
| J-CHS | 1 | 0 | 0 | 0 | 1 | 0 | 0 | 0 | 0 |
| mCriSTAL | 1 | 1 | 0 | 0 | 0 | 0 | 0 | 0 | 0 |
| MPI | 1 | 0 | 1 | 0 | 1 | 0 | 0 | 0 | 0 |
| Norton | 1 | 1 | 0 | 0 | 0 | 0 | 0 | 0 | 0 |
| nSFI | 1 | 1 | 0 | 0 | 1 | 0 | 0 | 0 | 0 |
| PRISMA-7 | 1 | 0 | 0 | 0 | 0 | 0 | 0 | 0 | 0 |
| SFS | 1 | 0 | 0 | 0 | 1 | 0 | 0 | 0 | 0 |
| Share-FI | 1 | 1 | 1 | 0 | 1 | 0 | 0 | 0 | 0 |
| VESPA | 1 | 0 | 0 | 0 | 1 | 0 | 1 | 0 | 1 |
| TFI | 1 | 0 | 0 | 0 | 1 | 0 | 0 | 0 | 0 |

Table continues on next page…

Table continued…

| **Frailty Tool** | **Total No. Studies** | **Overall Survival**  **(long term)** | **Disease-Free Survival** | **Cost** | **Any Geriatric Outcome** | **Delirium** | **Function** | **Mood** | **Cognition** |
| --- | --- | --- | --- | --- | --- | --- | --- | --- | --- |
| All Tools* | 35 | 11 | 4 | 1 | 12 | 8 | 7 | 1 | 3 |
| CFS | 30 | 6 | 3 | 1 | 1 | 0 | 3 | 0 | 0 |
| G8 | 22 | 3 | 0 | 0 | 1 | 0 | 1 | 1 | 1 |
| FP | 21 | 4 | 1 | 0 | 3 | 5 | 1 | 0 | 1 |
| VES-13 | 13 | 0 | 0 | 0 | 1 | 1 | 2 | 0 | 1 |
| FRAIL | 10 | 1 | 0 | 0 | 2 | 2 | 0 | 0 | 0 |
| TRST | 10 | 3 | 1 | 0 | 1 | 2 | 0 | 0 | 0 |
| EFS | 9 | 0 | 0 | 0 | 0 | 0 | 0 | 0 | 0 |
| GFI | 9 | 0 | 0 | 0 | 1 | 0 | 1 | 0 | 0 |
| mFI-11 | 9 | 1 | 0 | 0 | 1 | 0 | 2 | 0 | 0 |
| FI | 8 | 1 | 0 | 0 | 1 | 1 | 0 | 0 | 0 |
| KCL | 5 | 3 | 1 | 0 | 3 | 1 | 2 | 0 | 0 |
| RAI-C | 3 | 0 | 0 | 0 | 0 | 0 | 0 | 0 | 0 |
| MSK-FI | 3 | 0 | 0 | 0 | 0 | 0 | 0 | 0 | 0 |
| VMS | 3 | 0 | 0 | 0 | 0 | 1 | 0 | 0 | 0 |
| SOF | 3 | 1 | 0 | 0 | 0 | 0 | 0 | 0 | 0 |
| Balducci | 3 | 0 | 0 | 0 | 0 | 0 | 0 | 0 | 0 |
| GSS | 3 | 1 | 0 | 0 | 0 | 0 | 0 | 0 | 0 |
| EGSFI | 2 | 2 | 0 | 0 | 1 | 1 | 0 | 0 | 0 |
| aCGA | 2 | 0 | 0 | 0 | 0 | 0 | 0 | 0 | 0 |
| RFS | 2 | 0 | 0 | 0 | 0 | 0 | 0 | 0 | 0 |
| FRAILESEL | 1 | 0 | 0 | 0 | 0 | 0 | 0 | 0 | 0 |
| mCriSTAL | 1 | 0 | 0 | 0 | 0 | 0 | 0 | 0 | 0 |
| VESPA | 1 | 0 | 0 | 0 | 0 | 0 | 0 | 0 | 0 |
| nSFI | 1 | 0 | 0 | 0 | 0 | 0 | 0 | 0 | 0 |
| SFS | 1 | 0 | 0 | 0 | 0 | 0 | 0 | 0 | 0 |
| EFT | 1 | 0 | 0 | 0 | 0 | 0 | 0 | 0 | 0 |
| TFI | 1 | 0 | 0 | 0 | 0 | 0 | 0 | 0 | 0 |
| PRISMA-7 | 1 | 0 | 0 | 0 | 0 | 0 | 0 | 0 | 0 |
| GAPN | 1 | 0 | 0 | 0 | 0 | 0 | 0 | 0 | 0 |
| ISAR-HP | 1 | 0 | 0 | 0 | 0 | 0 | 0 | 0 | 0 |
| Norton | 1 | 0 | 0 | 0 | 0 | 0 | 0 | 0 | 0 |
| MPI | 1 | 0 | 0 | 0 | 0 | 0 | 0 | 0 | 0 |
| Share-FI | 1 | 0 | 0 | 0 | 0 | 0 | 0 | 0 | 0 |
| PFFS | 1 | 0 | 0 | 0 | 1 | 0 | 0 | 0 | 0 |
| J-CHS | 1 | 0 | 0 | 0 | 0 | 0 | 0 | 0 | 0 |

*The all tools row includes the total number of tools, not the number of studies, for each property.

# Supplementary Table S8. Comparisons Between Frailty Assessment Tools

| **Frailty Tool** | **Total No. Studies** | **Any Comparison** | **Compare Concurrent Criterion** | **Compare Predictive validity** | **Direct Comparison** | **Compared to Reference Standard** |
| --- | --- | --- | --- | --- | --- | --- |
| All Tools | 35 | 26 | 14 | 22 | 14 | 0 |
| CFS | 41 | 1 | 4 | 9 | 2 | 0 |
| FP | 26 | 1 | 5 | 7 | 3 | 0 |
| G8 | 26 | 1 | 3 | 17 | 4 | 0 |
| EFS | 15 | 1 | 4 | 3 | 1 | 0 |
| FRAIL | 15 | 1 | 4 | 5 | 2 | 0 |
| VES-13 | 13 | 1 | 2 | 9 | 2 | 0 |
| mFI-11 | 12 | 1 | 3 | 6 | 1 | 0 |
| TRST | 10 | 1 | 2 | 6 | 2 | 0 |
| FI | 9 | 1 | 1 | 2 | 2 | 0 |
| GFI | 9 | 1 | 2 | 5 | 2 | 0 |
| KCL | 8 | 1 | 1 | 1 | 0 | 0 |
| Balducci | 3 | 1 | 2 | 2 | 2 | 0 |
| GSS | 3 | 1 | 2 | 2 | 2 | 0 |
| MSK-FI | 3 | 1 | 0 | 1 | 0 | 0 |
| RAI-C | 3 | 0 | 0 | 0 | 0 | 0 |
| RFS | 3 | 1 | 1 | 1 | 1 | 0 |
| SOF | 3 | 0 | 0 | 0 | 0 | 0 |
| VMS | 3 | 0 | 0 | 0 | 0 | 0 |
| aCGA | 2 | 1 | 2 | 1 | 2 | 0 |
| EGSFI | 2 | 1 | 0 | 0 | 0 | 0 |
| PFFS | 2 | 0 | 0 | 0 | 0 | 0 |
| EFT | 1 | 1 | 0 | 1 | 0 | 0 |
| FRAILESEL | 1 | 0 | 0 | 0 | 0 | 0 |
| GAPN | 1 | 1 | 0 | 1 | 0 | 0 |
| ISAR-HP | 1 | 1 | 0 | 1 | 0 | 0 |
| J-CHS | 1 | 1 | 0 | 0 | 0 | 0 |
| mCriSTAL | 1 | 1 | 0 | 0 | 0 | 0 |
| MPI | 1 | 0 | 0 | 0 | 0 | 0 |
| Norton | 1 | 1 | 0 | 1 | 0 | 0 |
| nSFI | 1 | 1 | 0 | 1 | 0 | 0 |
| PRISMA-7 | 1 | 1 | 0 | 0 | 0 | 0 |
| SFS | 1 | 0 | 0 | 0 | 0 | 0 |
| Share-FI | 1 | 1 | 0 | 1 | 0 | 0 |
| VESPA | 1 | 0 | 0 | 0 | 0 | 0 |
| TFI | 1 | 0 | 0 | 1 | 1 | 0 |

# Supplementary Table S9. Brief Description of Tool Characteristics

| **Frailty Tool** | **Brief Description of Tool Characteristics^a^** |
| --- | --- |
| Clinical Frailty Scale (CFS) | 9-point ordinal scale, assigned by clinician as a global judgement after unstructured prompting questions. Ordinal scale of frailty severity. No special equipment needed. Basic training required. Websites with supporting materials and training available. |
| Geriatric 8 (G8) | 8-item questionnaire; multiple choice; across multiple domains. Threshold cut-off. No special training or equipment. Can be self-administered. A modified version of G8 that also includes instrumental activities of daily living has been studied |
| Frailty Phenotype (FP) | 5-items including physical performance measurement and self-report questions. Based on phenotype model of frailty. Threshold cut-off. Requires training and special equipment (dynamometer, measured walking distance). |
| Vulnerable Elder Scale  (VES-13) | 4-item questionnaire; Likert and Yes/No; focusing largely on functional ability, designed to be self-administered. Threshold cut-off. No special training or equipment. |
| FRAIL  (FRAIL Scale) | 5-item questionnaire; Yes/No, scale; multiple domains. Threshold cut-off. No special training or equipment. |
| Triage Risk Screening Tool  (TRST) | 5-item questionnaire; Yes/No. Threshold cut-off. No special training or equipment. Cannot be self-administered |
| Edmonton Frailty Scale (EFS) | 11-item questionnaire; Yes/No, Likert, and cognitive and physical performance tests; nine domains. Ordinal scale of frailty severity. Requires training and special equipment (clock draw, measured walking distance); but self-report version developed awaiting further testing. Websites with supporting materials and training available. |
| Groningen Frailty Index (GFI) | 15-item questionnaire; Yes/No; multiple geriatric domains. Threshold cut-off. No special training or equipment. Can be self-administered. |
| Modified Frailty Index-11  (mFI-11) | 11-item questionnaire; Yes/No; 9 comorbidities, one cognition, one functional status. A 5-item version exists, but not included in this review. Items generated by mapping Frailty Index variables onto those collected in the NSQIP database. Not based on a conceptual model of frailty. Threshold cut-off. No special training or equipment. |
| Frailty Index  (FI) | Multi-item scale (versions range from 30 to 54 items) to identify age- and health-related deficits meeting specific criteria across multiple domains including questionnaires and performance tests. Based on cumulative deficits model of frailty. Creates and index of frailty severity. Training and special equipment needed (depends on version used). |
| Kihon Checklist  (KCL) | 25-item questionnaire; Yes/No; multiple domains. Threshold cut-off. No special training or equipment. Can be self-administered. |
| Risk Analysis Index – Clinical  (RAI-C) | 14-item questionnaire; Yes/No, multiple choice; several domains. Threshold cut-off. No special equipment. Cannot be self-administered. |

^a^ This table focuses on the 12 most extensively investigated tools; full details and tools can be found in Supplementary Table S10.

# Supplementary Table S10. Frailty assessment tool characteristics

| **Total No. Studies** | **Tool type** | **Geriatric Domains** | **Scale** | **Scoring Method** | **Language Versions** | **Tool Development** | **Access to Tool** | **License Needed** | **Conceptual Framework** | **Training Required** | **Administration Time** | **Mode of Administration** | **Special Equipment** |
| --- | --- | --- | --- | --- | --- | --- | --- | --- | --- | --- | --- | --- | --- |
| **CFS**  9-point ordinal scale, assigned by clinician as a global judgement after unstructured prompting questions. Websites with supporting materials and training available.  Sources  [https://www.dal.ca/sites/gmr/our-tools/clinical-frailty-scale.html Rockwood K, Song X, MacKnight C, Bergman H, Hogan DB, McDowell I, Mitnitski A. A global clinical measure of fitness and frailty in elderly people. CMAJ. 2005;173(5):489-495. Rockwood K, Theou O. Using the Clinical Frailty Scale in allocating scarce health care resources. Can Geriatr J. 2020;23:254-259.](https://www.dal.ca/sites/gmr/our-tools/clinical-frailty-scale.htmlRockwood%20K,%20Song%20X,%20MacKnight%20C,%20Bergman%20H,%20Hogan%20DB,%20McDowell%20I,%20Mitnitski%20A.%20A%20global%20clinical%20measure%20of%20fitness%20and%20frailty%20in%20elderly%20people.%20CMAJ.%202005;173(5):489-495.Rockwood%20K,%20Theou%20O.%20Using%20the%20Clinical%20Frailty%20Scale%20in%20allocating%20scarce%20health%20care%20resources.%20Can%20Geriatr%20J.%202020;23:254-259.) | | | | | | | | | | | | | |
| 30 | Judgement | Functional independence; Falls; Comorbidity | Ordinal | Ordinal; Threshold | Multiple | Non-Surgical | Publication; Website | No | Cumulative deficits model (Rockwood Frailty Index) | Yes | NR | Clinician | No |
| **G8**  8-item questionnaire; multiple choice; across multiple domains    Resources  Bellera CA, Rainfray M, Mathoulin-Pélissier S, Mertens C, Delva F, Fonck M, et al. Screening older cancer patients: first evaluation of the G-8 geriatric screening tool. Ann Oncol 2012;23:2166–72. | | | | | | | | | | | | | |
| 22 | Questionnaire | Falls; Nutrition; Polypharmacy; Cognition; Mood | Continuous | Sum; Threshold | English; Korean | Non-Surgical | Publication; Website | No | Multidimensional geriatric domains | No | NR | Either | No |
| **FP**  5-items including physical performance measurement and self-report questions  Sources  Fried LP, Tangen CM, Walston Jet al. Frailty in older adults: evidencefor a phenotype: cardiovascular health study collaborative researchgroup.J Gerontol A Biol Sci Med Sci2001;56: M146–M156. | | | | | | | | | | | | | |
| 21 | Mixed; Questionnaire; Performance | Physical performance; Nutrition | Categorical | Sum; Threshold | English | Non-Surgical | Publication | No | Phenotype model (Fried) | No | NR | Clinician | Yes (dynamometer, measured walking distance) |
| **VES-13**  4-item questionnaire; Likert and Yes/No; focusing largely on functional ability, designed to be self-administered  Sources  Vernon 2019 Saliba s, Elliott m, Rubenstein la et al.: The Vulnerable Elders Survey (VES-13): ATool for Identifying Vulnerable Elders in the Community. jaGs 2001; 49: 1691-99 | | | | | | | | | | | | | |
| 13 | Questionnaire | Functional independence; Physical performance | Continuous | Sum; Threshold | English | Non-Surgical | Publication; Website | No | Multidimensional geriatric domains | No | NR | Patient | No |

Table continues on next page…

| **Total No. Studies** | **Tool type** | **Geriatric Domains** | **Scale** | **Scoring Method** | **Language Versions** | **Tool Development** | **Access to Tool** | **License Needed** | **Conceptual Framework** | **Training Required** | **Administration Time** | **Mode of Administration** | **Special Equipment** |
| --- | --- | --- | --- | --- | --- | --- | --- | --- | --- | --- | --- | --- | --- |
| **FRAIL**  5-item questionnaire; Yes/No, scale; multiple domains  Sources  Wilson 2021 Abellan van Kan G, Rolland YM, Morley JE, Vellas B. Frailty: toward a clinical definition. J Am Med Dir Assoc. 2008 | | | | | | | | | | | | | |
| 10 | Questionnaire | Mobility; Comorbidities; Nutrition | Continuous | Threshold | English | Non-Surgical | Publication | No | Phenotype model (Fried) | No | NR | Either | No |
| **TRST**  5-item questionnaire. Yes/No. Minor modifications from initial version which was developed for emergency room.  Sources  Meldon SW, Mion LC, Palmer RM, Drew BL, Connor JT, Lewicki LJ, Bass DM, Emerman CL. A brief risk-stratification tool to predict repeat emergency department visits and hospitalizations in older patients discharged from the emergency department. Acad Emerg Med. 2003 Mar;10(3):224-32. | | | | | | | | | | | | | |
| 10 | Questionnaire | Functional independence; Falls; Polypharmacy/Medications; Social Support; Cognition | Continuous | Sum; Threshold | English | Non-Surgical | Publication | No | Multidimensional geriatric domains | No | NR | Clinician | No |
| **EFS**  11-item questionnaire; Yes/No, Likert, and cognitive and physical performance tests; nine domains. Multiple versions for different settings available. Websites with supporting materials and training available.  Sources  [https://edmontonfrailscale.org/  He 2020 Rolfson DB, Majumdar SR, Tsuyuki RT, Tahir A, Rockwood K. Validity and reliability of the Edmonton Frail Scale.Age  Ageing2006;35: 526–9](https://edmontonfrailscale.org/%20He%202020Rolfson%20DB,%20Majumdar%20SR,%20Tsuyuki%20RT,%20Tahir%20A,%20Rockwood%20K.%20Validity%20and%20reliability%20of%20the%20Edmonton%20Frail%20Scale.Age %20Ageing2006;35:%20526–9) | | | | | | | | | | | | | |
| 9 | Mixed; Questionnaire; Performance | Functional independence; Physical performance; Nutrition; Polypharmacy/Medications; Social Support; Cognition; Mood; Other: Continence | Continuous | Ordinal; Threshold | Multiple | Non-Surgical | Publication; Website | Yes (some licenses free) | Multidimensional geriatric domains | Yes | 3.75 (SD 0.95) minutes | Clinician | Yes (clock draw, measured walking distance); some versions include no performance testing |

Table continues on next page…

| **Total No. Studies** | **Tool type** | **Geriatric Domains** | **Scale** | **Scoring Method** | **Language Versions** | **Tool Development** | **Access to Tool** | **License Needed** | **Conceptual Framework** | **Training Required** | **Administration Time** | **Mode of Administration** | **Special Equipment** |
| --- | --- | --- | --- | --- | --- | --- | --- | --- | --- | --- | --- | --- | --- |
| **GFI**  15-item questionnaire; Yes/No; focusing on multiple geriatric domains.  Sources  Steverink  N,  Slaets  JPJ,  Schuurmans  H,  et  al.  Measuring  frailty:  developing and testing the GFI  (Groningen  Frailty Indicator).Gerontologist. 2001;41:236. Metzelthin SF, Daniëls R, van Rossum E, de Witte L, van den Heuvel WJ, Kempen GI. The psychometric properties of three self-report screening instruments for identifying frail older people in the community. BMC public health. 2010 Dec;10:1-8. | | | | | | | | | | | | | |
| 9 | Questionnaire | Functional independence; Comorbidities; Nutrition; Polypharmacy/Medications; Social Support; Cognition; Mood; Other: Vision and Hearing | Continuous | Sum; Threshold | English | Non-Surgical | Publication; Website | No | Multidimensional geriatric domains | No | NR | Either | No |
| **mFI-11**  11-item questionnaire; Yes/No; 9 comorbidities, 1 cognition, 1 functional status. A 5-item version exists, but not included in this review. Items generated by mapping Frailty Index variables onto those collected in the NSQIP database  Sources  Suzuki 2021 Velanovich V, Antoine H, Swartz A, Peters D, Rubinfeld I. Accumulating deficits model of frailty and postoperative mortality and morbidity: its application to a national database. J Surg Res. 2013 Jul;183(1):104-10 | | | | | | | | | | | | | |
| 9 | Questionnaire | Functional independence; Comorbidities; Cognition | Continuous | Threshold | English | Surgical | Publication | No | None | No | NR | Clinician | No |
| **FI**  Multi-item scale (versions range from 30 to 54 items) to identify age- and health-related deficits meeting specific criteria across multiple domains including questionnaires and performance tests.  Sources  Aguilar-Frasco 2021 Rockwood K, Mitnitski A, MacKnight C. Some mathematical models of frailty and their clinical implications. Rockwood K, Mitnitski A: Frailty in relation to the accumulation of deficits. J Gerontol Biol Sci Med Sci 2007, 62A:722-727. Searle SD, Mitnitski A, Gahbauer EA, Gill TM, Rockwood K. A standard procedure for creating a frailty index. BMC Geriatr. 2008 Sep 30;8:24 | | | | | | | | | | | | | |
| 8 | Mixed; Questionnaire; Performance | Functional independence; Comorbidities; Nutrition; Polypharmacy/Medications; Social Support; Cognition; Mood; Other: Vision and Hearing | Continuous | Index | English | Non-Surgical | Publication | No | Cumulative deficits model (Rockwood Frailty Index) | No | NR | Clinician | Yes (depends on version used) |
| **KCL**  25-item questionnaire; Yes/No; multiple domains  Sources  Tanaka 2021 Satake S, Senda K, Hong YJ, et al. Validity of the Kihon Checklist for assessing frailty status. Geriatr Gerontol Int. 2016;16:709–715 | | | | | | | | | | | | | |
| 5 | Questionnaire | Functional independence; Falls; Nutrition; Social Support; Cognition; Mood | Continuous | Sum; Threshold | English, Japanese | Non-Surgical | Publication | No | Multidimensional geriatric domains | No | NR | Either | No |

Table continues on next page…

| **Total No. Studies** | **Tool type** | **Geriatric Domains** | **Scale** | **Scoring Method** | **Language Versions** | **Tool Development** | **Access to Tool** | **License Needed** | **Conceptual Framework** | **Training Required** | **Administration Time** | **Mode of Administration** | **Special Equipment** |
| --- | --- | --- | --- | --- | --- | --- | --- | --- | --- | --- | --- | --- | --- |
| **RAI-C**  14-item questionnaire; Yes/No, multiple choice; several domains  Sources  Varley 2020 Hall DE, Arya S, Schmid KK, Blaser C, Carlson MA, Bailey TL, Purviance G, Bockman T, Lynch TG, Johanning J. Development and Initial Validation of the Risk Analysis Index for Measuring Frailty in Surgical Populations. JAMA Surg. 2017 Feb 1;152(2):175-182. | | | | | | | | | | | | | |
| 3 | Questionnaire | Functional independence; Comorbidities; Nutrition; Cognition | Continuous | Sum; Threshold | English | Surgical | Publication | No | Cumulative deficits model (Rockwood Frailty Index) | No | NR | Clinician | No |
| **MSK-FI**  11-item quesitonnaire; Yes/No; 9 comorbidities, 1 cognition, 1 functional status.  Sources  Shahrokni 2019 | | | | | | | | | | | | | |
| 3 | Questionnaire | Functional independence; Comorbidities; Cognition | Continuous | Sum | English | Surgical | Publication | No | None | No | NR | Clinician | No |
| **VMS**  10-item questionnaire; Yes/No; across four domains  Sources  Argillander 2022 | | | | | | | | | | | | | |
| 3 | Questionnaire | Functional independence; Falls; Nutrition; Cognition | Ordinal | Threshold | Dutch, English | Non-Surgical | Publication; Website (Dutch) | No | Multidimensional geriatric domains | No | NR | Either | No |
| **SOF**  3-items including physical performance measurement and self-report question  Sources  Ensrud KE, Ewing SK, Taylor BC, Fink HA, Cawthon PM, Stone KL, et al. Comparison of 2 frailty indexes for prediction of falls, disability, fractures, and death in older women. Arch Intern Med. 2008;168:382–9. EnsrudKE,EwingSK,CawthonPM,FinkHA,TaylorBC,CauleyJA,etal.Acomparison of frailty indexes for the prediction of falls, disability, fractures, and mortality in older men. J Am Geriatr Soc. 2009;57:492–8. | | | | | | | | | | | | | |
| 3 | Mixed | Physical performance (a physical test); Nutrition; Other: Energy | Ordinal | Ordinal; Threshold | English | Non-Surgical | Publication | No | Phenotype model (Fried) | No | NR | Clinician | No |

Table continues on next page…

| **Total No. Studies** | **Tool type** | **Geriatric Domains** | **Scale** | **Scoring Method** | **Language Versions** | **Tool Development** | **Access to Tool** | **License Needed** | **Conceptual Framework** | **Training Required** | **Administration Time** | **Mode of Administration** | **Special Equipment** |
| --- | --- | --- | --- | --- | --- | --- | --- | --- | --- | --- | --- | --- | --- |
| **Balducci**  4-item criteria including age and geriatric domains  Sources  Balducci L, Beghe C. The application of the principles of geriatrics to the management of the older person with cancer. Crit Rev Oncol Hematol. 2000 Sep;35(3):147-54. | | | | | | | | | | | | | |
| 3 | Questionnaire | Functional independence; Comorbidities; | Categorical | Ordinal; Threshold | English | Non-Surgical | Publication | No | Multidimensional geriatric domains | No | NR | Clinician | No |
| **GSS**  Sum of two or more deficits across a group of geriatric screening tests  Sources  Kenning 2025b | | | | | | | | | | | | | |
| 3 | Mixed; Questionnaire; Performance | Functional independence; Comorbidities; Nutrition; Polypharmacy/Medications; Cognition; Mood | Ordinal | Threshold | English | Non-Surgical | Publication | No | Multidimensional geriatric domains | Yes | NR | Clinician | Yes (clock draw, measured walking distance) |
| **EGSFI**  15-item questionnaire; Yes/No, multiple choice; Multiple domains derived from the Frailty Index  Sources  Orouji Jokar 2016 | | | | | | | | | | | | | |
| 2 | Questionnaire | Functional independence; Comorbidities; Nutrition; Mood | Continuous | Index | English | Surgical | Publication | No | Cumulative deficits model (Rockwood Frailty Index) | No | NR | Clinician | No |
| **aCGA**  15-item questionnaire; Yes/No, multiple choice, cogntiver performance test; multiple domains  Sources  Overcash JA, Beckstead J, Extermann M, Cobb S. The abbreviated comprehensive geriatric assessment (aCGA): a retrospective analysis. Crit Rev Oncol Hematol. 2005 May;54(2):129-36. Overcash JA, Beckstead J, Moody L, Extermann M, Cobb S. The abbreviated comprehensive geriatric assessment (aCGA) for use in the older cancer patient as a prescreen: scoring and interpretation. Crit Rev Oncol Hematol. 2006 Sep;59(3):205-10. | | | | | | | | | | | | | |
| 2 | Mixed; Questionnaire; Performance | Functional independence; Cognition; Mood | Continuous | Threshold | English | Non-Surgical | Publication | No | Multidimensional geriatric domains | No | NR | Clinician | No |
| **RFS**  Assessment across 7 geriatric domains  Sources  Robinson 2013 Robinson TN, Wallace JI, Wu DS, Wiktor A, Pointer LF, Pfister SM, et al. Accumulated frailty characteristics predict postoperative discharge institu- tionalization in the geriatric patient. J Am Coll Surg 2011;213(1):37e42. | | | | | | | | | | | | | |
| 2 | Mixed; Questionnaire; Performance | Functional independence; Physical performance; Falls; Comorbidities; Nutrition; Cognition | Ordinal | Threshold | English | Surgical | Publication | No | Multidimensional geriatric domains | No | NR | Clinician | Yes (clock draw, measured walking distance) |

Table continues on next page…

| **Total No. Studies** | **Tool type** | **Geriatric Domains** | **Scale** | **Scoring Method** | **Language Versions** | **Tool Development** | **Access to Tool** | **License Needed** | **Conceptual Framework** | **Training Required** | **Administration Time** | **Mode of Administration** | **Special Equipment** |
| --- | --- | --- | --- | --- | --- | --- | --- | --- | --- | --- | --- | --- | --- |
| **FRAILESEL**  14-item tool; comorbidities, questionairres, performance tests  Sources  Costa 2021 | | | | | | | | | | | | | |
| 1 | Mixed; Questionnaire; Performance | Functional independence; Physical performance (a physical test); Falls; Comorbidity; Cognition | Continuous | Ordinal | English | Surgical | Publication | No | None | No | NR | Clinician | Yes (measured walking distance) |
| **mCriSTAL**  26-item questionnaire; multiple choice and Yes/No; clinical assessment  Sources  Eguaras Cordoba 2021 | | | | | | | | | | | | | |
| 1 | Mixed; Questionnaire; Laboratory values; Clinical assessment | Falls; Comorbidity; Nutrition; Cognition | Continuous | Sum; Threshold | English | Non-Surgical | Publication |  | None | Yes | NR | Clinician | Yes (labaroty values, ECG, clinical assessment) |
| **VESPA**  6-item questionnaire  Sources  Pollock 2022 | | | | | | | | | | | | | |
| 1 | Questionnaire | Functional independence; Comorbidities | Continuous | Sum; Threshold | English | Surgical | Publication | No | None | No | NR | Clinician | No |
| **nSFI**  11-item quesitonnaire; Yes/No  Sources  Sacco 2018 | | | | | | | | | | | | | |
| 1 | Questionnaire | Functional independence; Comorbidities; Nutrition; Polypharmacy/Medications; Mood | Continuous | Ordinal; Threshold | English | Surgical | Publication | No | None | No | NR | Clinician | No |

Table continues on next page…

| **Total No. Studies** | **Tool type** | **Geriatric Domains** | **Scale** | **Scoring Method** | **Language Versions** | **Tool Development** | **Access to Tool** | **License Needed** | **Conceptual Framework** | **Training Required** | **Administration Time** | **Mode of Administration** | **Special Equipment** |
| --- | --- | --- | --- | --- | --- | --- | --- | --- | --- | --- | --- | --- | --- |
| **SFS**  4-item; performance, yes/no, clinical assessment, lab value  Sources  Revenig 2015 | | | | | | | | | | | | | |
| 1 | Mixed; Performance; Questionnaire; Laboratory values; Clinical assessment | Physical performance (a physical test); Nutrition | Ordinal | Ordinal | English | Surgical | Publication | No | None | No | NR | Clinician | Yes (dynamometer, lab values, clinical assessment) |
| **EFT**  4-item tool; physical and cognitive test and lab values  Sources  Afilalo J, Lauck S, Kim DH, Lefèvre T, et al. Frailty in Older Adults Undergoing Aortic Valve Replacement: The FRAILTY-AVR Study. J Am Coll Cardiol. 2017 Aug 8;70(6):689-700. doi: 10.1016/j.jacc.2017.06.024. Epub 2017 Jul 7. PMID: 28693934. | | | | | | | | | | | | | |
| 1 | Mixed; Performance; Laboratory | Physical performance (a physical test); Nutrition | Continuous | Sum; Threshold | English | Surgical | Publication | No | Multidimensional geriatric domains | Yes | NR | Clinician | Yes (Cognitive test with Mini Mental Status Examination, Laboratory values) |
| **TFI**  15-item questionnaire; Yes/No; multiple domains  Sources  Gobbens RJJ, van Assen MALM, Luijkx KG, Wijnen-Sponselee MTh, Schols JMGA. The Tilburg Frailty Indicator: psychometric properties. J Am Med Dir Assoc 2010; 11(5):344-355. | | | | | | | | | | | | | |
| 1 | Questionnaire | Falls; Nutrition; Social Support; Cognition; Mood; Other | Ordinal | Threshold | Multiple | Non-Surgical | Publication (paper cites tool/report of tool) | Multidimensional geriatric domains | No | NR | Either | No | Questionnaire |
| **PRISMA-7**  7-item questionnaire; yes/no  Sources  Raiche M, Hebert R, Dubois M. PRISMA-7: a case-finding tool to identify older adults with moderate to severe disabilities. Arch Gerontol Geriatr. 2008;47:9–18. | | | | | | | | | | | | | |
| 1 | Questionnaire | Fuctional independence; Falls; Social support | Continuous | Threshold | English | Non-Surgical | Publication | No | None | No | NR | Clinician | No |
| **GAPN**  7-item combination of screening tools, performance tests, questionnaire  Sources  Wunderle 2021 | | | | | | | | | | | | | |
| 1 | Mixed; Questionnaire; Performance | Functional independence, Physical performance, Comorbidity, Cognition | Continuous | Sum; Threshold | English | Surgical | Publication | No | None | Yes | NR | Clinician | Yes (Cognitive test with Mini Mental Status Examination, dynamometer) |

Table continues on next page…

| **Total No. Studies** | **Tool type** | **Geriatric Domains** | **Scale** | **Scoring Method** | **Language Versions** | **Tool Development** | **Access to Tool** | **License Needed** | **Conceptual Framework** | **Training Required** | **Administration Time** | **Mode of Administration** | **Special Equipment** |
| --- | --- | --- | --- | --- | --- | --- | --- | --- | --- | --- | --- | --- | --- |
| **ISAR-HP**  4-item questionnaire; Yes/No  Sources  Hoogerduijn JG, Buurman BM, Korevaar JC, Grobbee DE, de Rooij SE, Schuurmans MJ. The prediction of functional decline in older hospitalised patients. Age Ageing. 2012 May;41(3):381-7. | | | | | | | | | | | | | |
| 1 | Questionnaire | Functional independence; Falls; Other = education | Continuous | Sum; Threshold | English | Non-Surgical | Publication | No | Multidimensional geriatric domains | No | NR | Either | No |
| **Norton**  5-item questionnaire; multiple choice  Sources  Trostchansky 2019 | | | | | | | | | | | | | |
| 1 | Questionnaire | Falls; Cognition; Other = incontinence | Continuous | Sum; Threshold | English | Non-Surgical | Website | No | None | No | NR | Clinician | No |
| **MPI**  8-item tool; combines several screening tools  Sources  Pilotto A, Ferrucci L, Franceschi M, et al. Development and valida- tion of a Multidimensional Prognostic Index for one‐year mortality from comprehensive geriatric assessment in hospitalized older patients. Rejuvenation Res. 2008;11:151–161. | | | | | | | | | | | | | |
| 1 | Questionnaire | Functional independence; Comorbidities; Nutrition; Polypharmacy/Medications; Social Support; Cognition; Other: pressure sore | Continuous | Index; Threshold | English | Non-Surgical | Publication | No | Multidimensional geriatric domains | Yes | NR | Clinician | Yes (other screening tools) |
| **Share-FI**  5-items including physical performance measurement and self-report questions  Sources  Romero-Ortuno R, Walsh CD, Lawlor BA, Kenny RA. A frailty instrument for primary care: findings from the Survey of Health, Ageing and Retirement in Europe (SHARE). BMC Geriatr. 2010 Aug 24;10:57. | | | | | | | | | | | | | |
| 1 | Mixed; Questionnaire; Performance | Physical performance; Nutrition | Continuous | Algorithm; Threshold | English | Non-Surgical | Publication | No | Phenotype model (Fried) | No | NR | Clinician | Yes (dynamometer) |

Table continues on next page…

| **Total No. Studies** | **Tool type** | **Geriatric Domains** | **Scale** | **Scoring Method** | **Language Versions** | **Tool Development** | **Access to Tool** | **License Needed** | **Conceptual Framework** | **Training Required** | **Administration Time** | **Mode of Administration** | **Special Equipment** |
| --- | --- | --- | --- | --- | --- | --- | --- | --- | --- | --- | --- | --- | --- |
| **PFFS**  14-item too; visual analog scales  Sources  [https://www.dal.ca/sites/gmr/our-tools/pictorial-fit-frail-scale.html Cooper 2022 Theou O, Andrew M, Ahip SS, et al. The Pictorial Fit-Frail Scale: Developing a Visual Scale to Assess Frailty. Can Geriatr J 2019;22(2):64–74](https://www.dal.ca/sites/gmr/our-tools/pictorial-fit-frail-scale.htmlCooper%202022Theou%20O,%20Andrew%20M,%20Ahip%20SS,%20et%20al.%20The%20Pictorial%20Fit-Frail%20Scale:%20Developing%20a%20Visual%20Scale%20to%20Assess%20Frailty.%20Can%20Geriatr%20J%202019;22(2):64–74) | | | | | | | | | | | | | |
| 1 | Questionnaire | Functional independence; Falls; Nutrition; Polypharmacy/Medications; Social Support; Cognition; Mood; Other: tiredness, vision, hearing, aggression, pain, bladder control | Continuous | Index; Sum | English | Non-Surgical | Publication; Website | Yes (for some uses) | Multidimensional geriatric domains | No | 3:31 (SD 2:09) minutes | Either | No |
| **J-CHS**  5-items including physical performance measurement and self-report questions  Sources  Kameyama 2021 | | | | | | | | | | | | | |
| 1 | Mixed; Questionnaire; Performance | Physical performance; Nutrition | Categorical | Sum; Threshold | Japanese | Non-Surgical | Publication | No | Phenotype model (Fried) | No | NR | Clinician | Yes (dynamometer, measured walking distance) |

# References

1.	Kirshner B, Guyatt G. A methodological framework for assessing health indices. *J Chronic Dis*. 1985;38(1):27-36.

2.	De Vet HC, Terwee CB, Mokkink LB, Knol DL. *Measurement in Medicine: A Practical Guide*. Cambridge university press; 2011.

3.	Mokkink LB, Terwee CB, Patrick DL, et al. The COSMIN study reached international consensus on taxonomy, terminology, and definitions of measurement properties for health-related patient-reported outcomes. *J Clin Epidemiol*. 2010;63(7):737-745.

4.	Mokkink LB, Terwee CB, Patrick DL, et al. The COSMIN checklist for assessing the methodological quality of studies on measurement properties of health status measurement instruments: an international Delphi study. *Qual Life Res*. 2010;19(4):539-549.

5.	Moons KGM, Altman DG, Reitsma JB, et al. Transparent Reporting of a multivariable prediction model for Individual Prognosis or Diagnosis (TRIPOD): explanation and elaboration. *Ann Intern Med*. 2015;162(1):W1-W73. doi:10.7326/M14-0698

6.	Cohen JF, Korevaar DA, Altman DG, et al. STARD 2015 guidelines for reporting diagnostic accuracy studies: explanation and elaboration. *BMJ Open*. 2016;6(11).

7.	Hemingway H, Croft P, Perel P, et al. Prognosis research strategy (PROGRESS) 1: a framework for researching clinical outcomes. *Bmj*. 2013;346.

8.	Steyerberg EW, Pencina MJ, Lingsma HF, Kattan MW, Vickers AJ, Van Calster B. Assessing the incremental value of diagnostic and prognostic markers: a review and illustration. *Eur J Clin Invest*. 2012;42(2):216-228.

9.	STRATOS initiative | The STRATOS initiative. Accessed April 25, 2024. https://www.stratos-initiative.org/

10.	Stanick CF, Halko HM, Nolen EA, et al. Pragmatic measures for implementation research: development of the Psychometric and Pragmatic Evidence Rating Scale (PAPERS). *Transl Behav Med*. 2021;11(1):11-20. doi:10.1093/tbm/ibz164

11.	Umemneku Chikere CM, Wilson K, Graziadio S, Vale L, Allen AJ. Diagnostic test evaluation methodology: A systematic review of methods employed to evaluate diagnostic tests in the absence of gold standard - An update. *PloS One*. 2019;14(10):e0223832. doi:10.1371/journal.pone.0223832

12.	Takwoingi Y, Partlett C, Riley RD, Hyde C, Deeks JJ. Methods and reporting of systematic reviews of comparative accuracy were deficient: a methodological survey and proposed guidance. *J Clin Epidemiol*. 2020;121:1-14. doi:10.1016/j.jclinepi.2019.12.007

13.	McInnes MDF, Moher D, Thombs BD, et al. Preferred Reporting Items for a Systematic Review and Meta-analysis of Diagnostic Test Accuracy Studies: The PRISMA-DTA Statement. *JAMA*. 2018;319(4):388-396. doi:10.1001/jama.2017.19163

14.	Whiting PF, Rutjes AW, Westwood ME, et al. QUADAS-2: a revised tool for the quality assessment of diagnostic accuracy studies. *Ann Intern Med*. 2011;155(8):529-536.

15.	Riley RD, Hayden JA, Steyerberg EW, et al. Prognosis Research Strategy (PROGRESS) 2: prognostic factor research. *PLoS Med*. 2013;10(2):e1001380.

16.	Steyerberg EW, Moons KG, van der Windt DA, et al. Prognosis Research Strategy (PROGRESS) 3: prognostic model research. *PLoS Med*. 2013;10(2):e1001381.

17.	Hingorani AD, van der Windt DA, Riley RD, et al. Prognosis research strategy (PROGRESS) 4: stratified medicine research. *Bmj*. 2013;346.

18.	Cohen JF, Korevaar DA, Altman DG, et al. STARD 2015 guidelines for reporting diagnostic accuracy studies: explanation and elaboration. *BMJ Open*. 2016;6(11):e012799.

19.	Takwoingi Y, Leeflang MMG, Deeks JJ. Empirical evidence of the importance of comparative studies of diagnostic test accuracy. *Ann Intern Med*. 2013;158(7):544-554. doi:10.7326/0003-4819-158-7-201304020-00006

20.	Hurria A, Levit LA, Dale W, et al. Improving the evidence base for treating older adults with cancer: American Society of Clinical Oncology statement. *J Clin Oncol*. 2015;33(32):3826-3833.

21.	Lundebjerg NE, Trucil DE, Hammond EC, Applegate WB. When It Comes to Older Adults, Language Matters: Journal of the American Geriatrics Society Adopts Modified American Medical Association Style. *J Am Geriatr Soc*. 2017;65(7):1386-1388. doi:10.1111/jgs.14941

22.	Clegg A, Young J, Iliffe S, Rikkert MO, Rockwood K. Frailty in elderly people. *The lancet*. 2013;381(9868):752-762.

23.	Robinson TN, Walston JD, Brummel NE, et al. Frailty for surgeons: review of a National Institute on Aging Conference on Frailty for Specialists. *J Am Coll Surg*. 2015;221(6):1083-1092.

24.	Anaya DA, Johanning J, Spector SA, et al. Summary of the panel session at the 38th Annual Surgical Symposium of the Association of VA Surgeons: what is the big deal about frailty? *JAMA Surg*. 2014;149(11):1191-1197.

25.	Ethun CG, Bilen MA, Jani AB, Maithel SK, Ogan K, Master VA. Frailty and cancer: implications for oncology surgery, medical oncology, and radiation oncology. *CA Cancer J Clin*. 2017;67(5):362-377.

26.	Morley JE, Vellas B, Van Kan GA, et al. Frailty consensus: a call to action. *J Am Med Dir Assoc*. 2013;14(6):392-397.

27.	Rodríguez-Mañas L, Féart C, Mann G, et al. Searching for an operational definition of frailty: a Delphi method based consensus statement. The frailty operative definition-consensus conference project. *J Gerontol Ser Biomed Sci Med Sci*. 2013;68(1):62-67.

28.	Buta BJ, Walston JD, Godino JG, et al. Frailty assessment instruments: systematic characterization of the uses and contexts of highly-cited instruments. *Ageing Res Rev*. 2016;26:53-61.

29.	Covidence - Better systematic review management. Covidence. Accessed January 31, 2021. https://www.covidence.org/

30.	Searle SD, Mitnitski A, Gahbauer EA, Gill TM, Rockwood K. A standard procedure for creating a frailty index. *BMC Geriatr*. 2008;8(1):1-10.

31.	Abbasi M, Rolfson D, Khera AS, Dabravolskaj J, Dent E, Xia L. Identification and management of frailty in the primary care setting. *CMAJ*. 2018;190(38):E1134-E1140.

32.	Mohile SG, Dale W, Somerfield MR, et al. Practical assessment and management of vulnerabilities in older patients receiving chemotherapy: ASCO guideline for geriatric oncology. *J Clin Oncol*. 2018;36(22):2326.

33.	Chesney TR, AlQurini N, Alibhai SM. Geriatric Surgical Oncology. In: *Surgical Oncology Manual*. Springer; 2020:251-273.
